# Changes in the quantity and quality of time use during the COVID-19 lockdowns in the UK: Who is the most affected?

**DOI:** 10.1371/journal.pone.0258917

**Published:** 2021-11-03

**Authors:** Ines Lee, Eileen Tipoe

**Affiliations:** 1 Faculty of Economics, University of Cambridge, Cambridge, United Kingdom; 2 School of Economics and Finance, Queen Mary University of London, London, United Kingdom; UCLA Fielding School of Public Health, UNITED STATES

## Abstract

We investigated changes in the quantity and quality of time spent on various activities in response to the COVID-19-induced national lockdowns in the UK. We examined effects both in the first national lockdown (May 2020) and the third national lockdown (March 2021). Using retrospective longitudinal time-use diary data collected from a demographically diverse sample of over 760 UK adults in both lockdowns, we found significant changes in both the quantity and quality of time spent on broad activity categories (employment, housework, leisure). Individuals spent less time on employment-related activities (in addition to a reduction in time spent commuting) and more time on housework. These effects were concentrated on individuals with young children. Individuals also spent more time doing leisure activities (e.g. hobbies) alone and conducting employment-related activities outside normal working hours, changes that were significantly correlated with decreases in overall enjoyment. Changes in quality exacerbated existing inequalities in quantity of time use, with parents of young children being disproportionately affected. These findings indicate that quality of time use is another important consideration for policy design and evaluation.

## Introduction

The COVID-19 pandemic has drastically affected our daily lives and will likely have lasting effects on lifestyles and work arrangements [1, 2]. Previous studies found that public health measures to contain the pandemic had different effects across sociodemographic groups, exacerbating inequalities in mental health, job security, and hours worked across various dimensions such as gender [3–6], ethnicity [7], age [8–10], and occupation [11, 12].

Aside from changing the total allocation of time across various activities (‘quantity’), the pandemic and associated mitigation measures may also have changed the way these activities are conducted (‘quality’). Our study contributes to this literature by examining another important yet under-studied dimension of inequality–quality of time spent on daily activities, which has been shown to affect wellbeing [13–15]. To do so, we collected detailed retrospective time-use diaries for a large demographically diverse sample of UK adults (N = 766), documenting the sequence and characteristics of activities conducted by each individual over a specified 24-hour period. We used this data to measure quantity of time spent on 4 broad activity categories: employment (excluding time spent travelling to/from work), housework (e.g. cooking), leisure (e.g. mass media consumption), and subsistence (sleeping, eating, and other personal care). We also constructed measures of quality of time use by focusing on factors that affect an individual’s experience of an activity, such as with whom the activity was done and the time at which the activity was performed.

While most studies focus on the early months of the pandemic, our data covers 3 timepoints over a 13-month period: pre-pandemic (February 2020), first national lockdown (May 2020), and third national lockdown (March 2021). Since the first and third national lockdowns were similar in all key respects (school and workplace closures, stay-at-home requirements, restrictions on movement within the UK; as detailed in Table S1 in S1 Appendix), we can examine the effects of repeated COVID-19 containment measures. The unique longitudinal nature of our data also captures within-person changes and adaptations as lockdowns and social distancing measures become part of everyday life. Within-person comparisons allow us to control for any unobserved variation across individuals that affects the outcome variables, for example differences in the way individuals report enjoyment on a Likert scale [16].

We documented significant and persistent changes in the quantity of time use: in both lockdowns, compared to the pre-pandemic timepoint, individuals spent more time on housework and less time on employment-related activities (conditional on remaining employed during either lockdown), with effects being concentrated on individuals with young children. Compared to the pre-pandemic timepoint, fewer individuals were employed during either lockdown, and females with young children were significantly less likely to be employed. We also found clear evidence that the quality of time use decreased during both lockdowns, with increases in leisure time spent alone and a larger proportion of individuals working unusual hours and conducting housework during standard working hours.

Our study shows that both quality and quantity of time use were important for self-reported enjoyment. Changes in daily routines and patterns of time use were significantly correlated with changes in overall enjoyment. Increases in leisure time were associated with increases in overall enjoyment, but these effects diminished if leisure time was spent alone. Deteriorations in work-life balance, indicated by employment activities conducted outside normal working hours, were negatively associated with overall enjoyment. To the extent that lockdowns and social distancing measures influence daily routines, the persistence of these changes could affect longer-term psychological well-being [17].

## Materials and methods

This study was approved by the Institutional Review Board at the University of Oxford (approval code ECONCIA20-21-16). Informed consent was provided by all survey participants prior to their participation and participants understood that they could withdraw from the study at any time.

We collected data in two waves. Wave 1 was conducted in May 2020, 7 weeks into the first national lockdown in the UK. We used the survey platform Prolific to recruit individuals who were over 18, had lived in the UK since December 2019, and were still in the labor market (including those unemployed and searching for work) in February 2020. Individuals provided information for the first two timepoints: pre-pandemic (defined as February 2020) and the first national lockdown. We then surveyed the same respondents 10 months later (Wave 2), 7 weeks into the third national lockdown. Section 1 in S1 Appendix contains more details about the study context and sample.

Our longitudinal sample consists of individuals who completed at least one time-use diary for each timepoint. Section 2.2 in S1 Appendix shows that our longitudinal sample does not significantly differ in sociodemographic characteristics (age, gender, ethnicity, education, and household composition) from the full sample who completed Wave 1 only.

Our sample was designed to be demographically diverse across gender, age, and ethnicity. Section 2.3 in S1 Appendix compares the composition of our sample and that of a nationally representative sample (Understanding Society) and shows some similarities in sociodemographic characteristics, though our sample is more educated and older on average. Our results are qualitatively similar when we reweighted our sample to match the composition of Understanding Society’s in-workforce sample across gender, age, ethnicity, education, and household composition (Section 8 in S1 Appendix).

For each timepoint, we asked respondents to retrospectively fill in time-use diaries for their most recent workday (if applicable) and non-workday. Time-use diaries record the chronological sequence of activities that respondents did over a 24-hour period through a series of ‘episodes’, and have been shown to give comparable data quality to objective real-time measures such as wearable cameras and accelerometers [18, 19]. The structure of our time-use diaries followed those used in the UK Time Use Survey (UKTUS), but for respondents’ ease of completion, we used pre-specified activities (42 total) categorized under 4 broad categories: leisure, employment (excluding time spent travelling to/from work), housework, and subsistence (sleeping, eating, and other personal care). Table 1 shows the activity subcategories used in our main analysis and types of activities included in each broad category. Section 3.1 in S1 Appendix specifies the detailed mapping between broad categories and pre-specified activities.

**Table 1:** Time use diaries: Mapping between activity subcategories and the broad activity categories used in our analysis.

Broad activity	Activity subcategories
Housework	Caring/Childcare
	Cooking/Groceries
	Cleaning
	Other housework (e.g. bills, household accounts, repairs)
Employment	Work tasks
	Meetings
	Searching for jobs
	Other employment-related activities (e.g. casual work)
Leisure	Social/cultural
	Arts/Hobbies
	Mass media consumption
	Physical exercise
	Volunteering
Subsistence	Sleeping
	Eating
	Personal care

See Section 3. in S1 Appendix 1 for the detailed mapping between pre-specified activities and broad categories.

For each episode within a time-use diary, respondents specified (1) the episode start and end time (with a minimum duration of 10 minutes per episode); (2) the main activity of that episode; (3) the secondary activity that the respondent was engaged in simultaneously (if any); (4) whom they did the activity with; (5) where they did the activity; (6) whether they used a device for that episode; (7) how much they enjoyed the activity (on a scale of 1 to 7).

Within a given diary day, there may be episodes with missing or mis-recorded data. We checked and cleaned each diary using a set of rules detailed in Section 3.2 in S1 Appendix, based on the UKTUS’ methodology. Most episodes did not require editing. For example, less than 0.01% of episodes had missing activities or missing start or end times. To check whether recall bias affected the quality of data, in Appendix, Section 3.3 in S1 Appendix we verified that mean pre-pandemic times spent on broad activity categories were similar to those obtained from a nationally representative survey (the 2014/15 UK Time Use Survey), as done by other COVID-19 studies on time use [20, 21].

We also collected the following sociodemographic information from each respondent: gender, year of birth, ethnicity, highest educational level, household composition, employment status, work arrangements at each timepoint, and monthly before-tax income. Section 4 in S1 Appendix provides more detail on the construction of our main variables and covariates.

Our analysis followed the procedures outlined in our pre-analysis plan (https://aspredicted.org/blind.php?x=3az7we), with extensions discussed in Section 5 in S1 Appendix. Analyses were conducted with Stata statistical software version 16.0. For inference, we used two-sided p-values and 95% confidence intervals.

## Results

### Changes in time use: Quantity

For each individual and timepoint, we calculated the total time spent on employment, housework, leisure, and subsistence as a main activity. Since a respondent completed up to 2 diary days per timepoint, we obtained a single value for each timepoint by dividing the total time spent by the total number of applicable diary days. Total time spent on housework, leisure, and subsistence were divided by 2 if a respondent completed both a workday diary and non-workday diary; total time spent on employment was not divided by 2 because there was at most one applicable diary day per timepoint.

**Fig 1:**
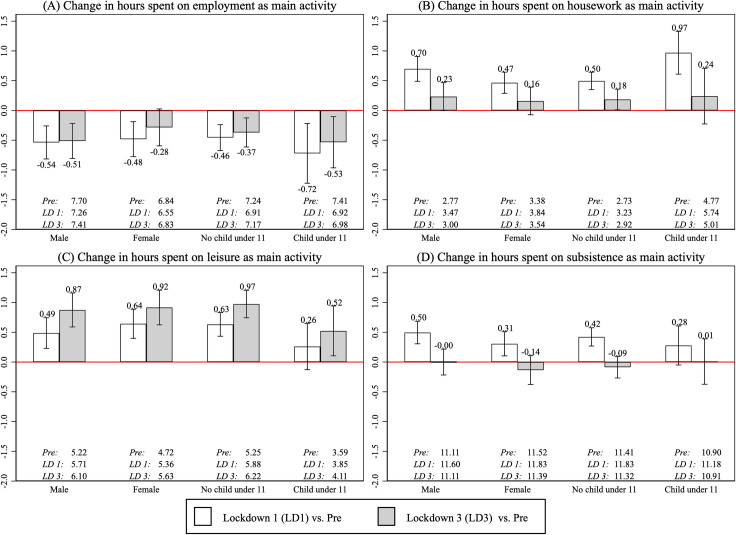
Within-person changes in time spent on 4 broad activity categories. Bars present average within-person changes in quantity of time use between the pre-pandemic timepoint (February 2020) and the first lockdown (May 2020) or the third lockdown (March 2021). Within-person changes for employment activities were calculated using the subset of individuals who remained employed in both periods of interest. Error bars represent 95% confidence intervals, and average levels for each subgroup are reported underneath the bars. Note that the conditional means were calculated separately (either by gender or household composition), so the four subgroups shown are not mutually exclusive.

Fig 1 shows average within-person differences in time spent per day on broad activity categories, comparing the pre-pandemic timepoint with the first and third lockdowns. We calculated average within-person differences separately by gender (female vs male) and household composition (living with at least one young child under 11 vs not living with a child under 11). In our main measure of time use, we categorized time spent according to the main activity. Section 6.1 in S1 Appendix presents additional results when time spent was categorized according to both the main and secondary activity, which are qualitatively similar to our main results.

Fig 1A shows that among people who were employed both pre-pandemic and during the lockdown in question, time spent on employment-related activities decreased by 17–43 minutes on average compared to before the pandemic. Although the direction of change was the same across population subgroups, the magnitude varies. When evaluated in both absolute and percentage terms, on average people living with at least one young child (aged 11 or under) saw a larger decrease in time spent on employment activities (0.72 x 60 = 43 minutes, 95% CI = [–74, –13]) during the first lockdown and a 32-minute decrease (95% CI = [–57, –5]) between pre-pandemic and the third lockdown. In comparison, people not living with young children saw an average decrease of 28 minutes (95% CI = [–40, –14]) during the first lockdown and 22 minutes (95% CI = [–37, –8]) between pre-pandemic and the third lockdown.

Aside from changes in the intensive margin, we also found substantial increases in the extensive margin (unemployment). Section 6.2 in S1 Appendix shows that before the pandemic, 86% of our sample was employed, but only 63% and 74% were employed during the first and third lockdown respectively. Our analysis in Table S16 in S1 Appendix also indicates heterogeneity in employment probabilities: controlling for pre-pandemic employment status, females with young children were significantly less likely to be employed than males without children across both lockdowns and significantly less likely to be employed than males with children during the third lockdown. Section 6.3 in S1 Appendix computes Gini coefficients to show that inequality in time spent on employment increased during both lockdowns compared to the pre-pandemic timepoint due to these changes in the extensive margin.

Fig 1B and 1D show that the first lockdown had a larger effect on time spent on housework and subsistence activities compared to the third lockdown. Among females, the average time spent on housework increased by 28 minutes (= 0.47 x 60; 95% CI = [17, 38]) during the first lockdown. Among males, the average time spent on subsistence activities increased by 30 minutes (95% CI = [18, 41]) during the first lockdown. However, these increases were largely reversed during the third lockdown for an overall mean-zero effect.

Fig 1C shows that changes in time spent on leisure activities were unequally distributed and larger during the third lockdown. Among individuals without children, average time spent on leisure activities increased by 38 minutes during the first lockdown and by 58 minutes during the third lockdown, relative to the pre-pandemic period. In contrast, individuals with young children experienced a moderate increase only during the third lockdown (31 minutes, 95% CI = [7, 57]).

### Correlation between time use during the lockdowns and sociodemographic characteristics

To further analyze how time spent varied across population subgroups, we examined the correlation between time use patterns during each lockdown and sociodemographic characteristics of respondents, controlling for pre-pandemic levels of time use. We used the following regression specification:

$${TS}_{i,LD}=\alpha +\beta^{\prime }W_{i}+\delta {TS}_{i,Pre}+\varepsilon_{i}$$ (1)

*TS_i,LD_* is respondent *i*’s time spent on one of the four broad activity categories, either measured as time spent on that activity during the first lockdown or during the third lockdown. ***W**_i_* is a vector of respondent characteristics: a binary indicator for female, age categories (5-year intervals from 25–29 to 60 or above, measured relative to 18-24-year-olds), a binary indicator for having a tertiary degree, a binary indicator for white ethnicity, categories for monthly income (intervals of 1000 GBP, measured relative to <1000 GBP), a binary indicator for working-from-home status during the first lockdown (if the dependent variable is for the first lockdown) or third lockdown (if the dependent variable is for the third lockdown), and a binary indicator for living with a child under 11. To capture potential gender differences in parental time allocation to childcare and housework [6], we also included an interaction term between female and living with young children in this vector. With the exception of working-from-home status, all respondent characteristics were taken from the pre-pandemic timepoint.

We included pre-pandemic levels in total time spent on an activity category (*TS_i,Pre_*) to account for the fact that time use patterns are persistent over time and that respondents who engaged in below-average/above-average levels of a particular activity would be unable to decrease/increase the amount of time spent by the same degree as respondents who engaged in moderate levels of that activity.

For regressions with employment as the dependent variable, we used a Heckman selection model to account for the possibility that individual characteristics such as gender and household composition affect the likelihood of remaining in employment during either lockdown [22]. Since every respondent participated in other non-employment activities, we did not apply Heckman corrections for the regressions with time spent on housework, leisure, or subsistence as the dependent variable. To satisfy the exclusion restriction of our Heckman employment selection equation, in addition to all variables in the vector ***W**_i_*, we included a binary indicator that equals 1 if the respondent was employed pre-pandemic and zero otherwise, and a continuous variable ranging from 0 to 100 measuring the percentage of time spent working from home in the pre-pandemic period. The coefficients on the Heckman selection equation (Table S16 in S1 Appendix) show that the likelihood of remaining employed during the first and third lockdowns increased with income (*p* < 0.05) but was significantly lower for females with young children (*p* < 0.05).

**Fig 2:**
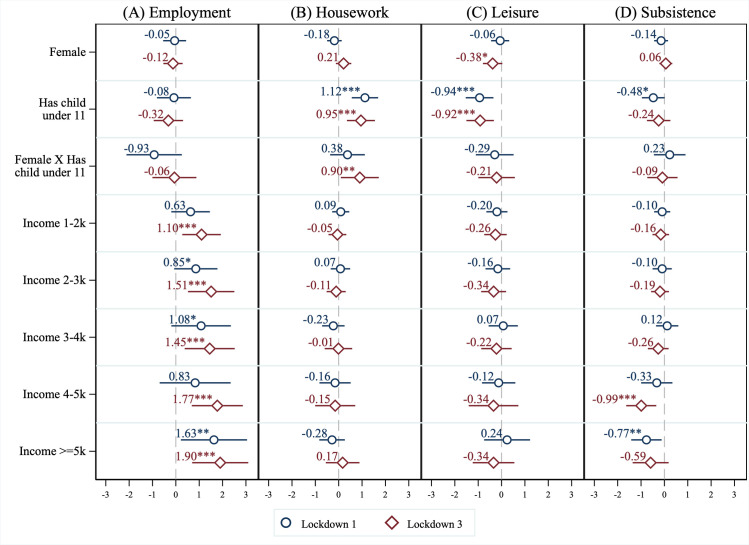
Correlates of time spent (hours per day) on broad activity categories. Lockdown 1 refers to May 2020 and Lockdown 3 refers to March 2021. In addition to the variables reported, we also controlled for age, education, working-from-home status, and pre-pandemic levels in total time spent on the given activity. Regressions with employment as the dependent variable used Heckman corrections to account for selection into employment. Point estimates are reported with 95% confidence intervals. The Heckman corrected regression (panel A) used bootstrapped standard errors (1000 replications); other regressions used robust standard errors (panels B-D). *** p < 0.01, ** p < 0.05, * p < 0.1.

The estimated coefficients from our time use regressions, shown in Fig 2, indicate variation in time use across gender, household composition, age, and income, with effects mainly concentrated on adults living with young children. Full regression tables are reported in Section 6.4 in S1 Appendix.

Fig 2A shows that conditional on remaining employed, females living with young children reduced their time spent on employment-related activities in the first lockdown by 64 minutes more per day compared to males without young children (= 60 x (-0.05–0.08–0.93); *p* < 0.05) and by 61 minutes more per day than females without young children (= 60 x (-0.08–0.93); *p* < 0.05). In contrast, high-income individuals (earning £5000 per month or more) worked almost two hours (*p* < 0.01) more per day in the third lockdown than employed individuals earning less than £1000 per month. In Table S17 in S1 Appendix, we also present results without applying the Heckman correction. The coefficients are qualitatively similar to those in our main specification, suggesting that our results for employment are not primarily driven by selection effects. Table S18 in S1 Appendix provides a breakdown of time use by subcategories in Table 1 and shows that this decrease was mainly due to a reduction in time spent on work tasks, rather than meetings and other employment activities.

Fig 2B shows that living with young children was associated with a 57-minute (*p* < 0.01) per day increase in housework during the third lockdown, and females living with young children did an extra 67 minutes (= 60 x (0.21+0.90); *p* < 0.05) of housework compared to males living with young children. We did not find evidence of differential effects by income brackets. Table S19 in S1 Appendix shows a breakdown of time use by housework subcategories in Table 1, and suggests a gendered division of housework for cooking and cleaning. Only females experienced an increase in time spent on cooking and cleaning, whereas increases in time spent on caring duties were experienced by both males and females with young children.

Fig 2C shows that living with young children was associated with a decrease in leisure time of 56-minutes per day (*p* < 0.01) during the first lockdown and a decrease of 55-minutes (*p* < 0.01) during the third lockdown. We did not find evidence of differential effects by income brackets. Table S20 in S1 Appendix breaks down leisure activities into the subcategories from Table 1. The results indicate that among females and individuals with young children, the decrease in leisure time was driven by a reduction in time spent on hobbies (consisting of active leisure activities), especially during the first lockdown. Given that the positive relationship between leisure time and mental health operates through active rather than passive leisure [23–26], the overall effect of increased lockdown leisure on wellbeing is unclear *a priori*.

Lastly, Fig 2D shows that individuals with higher incomes (earning £4000 per month or more) spent less time on subsistence activities during both lockdowns. Table S21 in S1 Appendix provides a breakdown of changes in time use by subsistence activity subcategories and indicates that this decrease was spread across all subsistence activities rather than being concentrated on a particular activity.

### Changes in time use: Quality

Aside from changing total time spent on activities, the pandemic may also have affected the way that individuals conduct certain activities, which in turn influences their enjoyment of time spent on those activities. The psychological and sociological literature considers any factor that affects episode-specific enjoyment beyond the specific activity conducted as an indicator of quality [27–29].

Using this definition, we focused on 4 measures of quality.

1. **Multitasking.** Multitasking, defined as the simultaneous performance of more than one task or type of activity [30], can enable individuals to meet the competing demands of work and home [31, 32] but has been linked to feelings of time stress [33] and lower activity-specific enjoyment [34, 35]. We considered a subset of multitasking behaviors where respondents conducted activities in different broad categories (such as housework and employment). For each respondent and timepoint, we calculated the total time spent on episodes that contain both a main and secondary activity, where the main and secondary activities belong to different broad categories (e.g. employment as main activity, housework as secondary activity).
2. **Leisure time spent alone.** Conducting leisure activities with other individuals is associated with higher instantaneous satisfaction [36–38] and better health outcomes in the long run [15, 39, 40]. For each respondent and timepoint, we calculated the total time spent on episodes where the activity category was ‘leisure’ and was conducted alone.
3. **Working atypical hours.** Conducting employment-related activities on non-workdays and outside typical working hours affects one’s ability to spend leisure time with others [41, 42] and is associated with poorer mental health [14, 43]. We defined unusual work hours as any employment-related activity conducted outside standard working hours (the time window of 8.30–17.30 on a workday), which includes employment-related activities conducted on a non-workday, and job searching activities for the unemployed. The time window was determined by taking the median start and end time of employment activities across all respondents’ pre-pandemic workday diaries.
4. **Doing housework during typical working hours.** This measure of ‘unusual’ housework hours captures the inability to clearly delineate boundaries between work and family life, which is associated with lower job satisfaction and job performance, and negative long-term health outcomes [13, 44]. We measured unusual housework hours as any housework-related activity conducted within standard working hours (8.30–17.30 on a workday).

**Fig 3:**
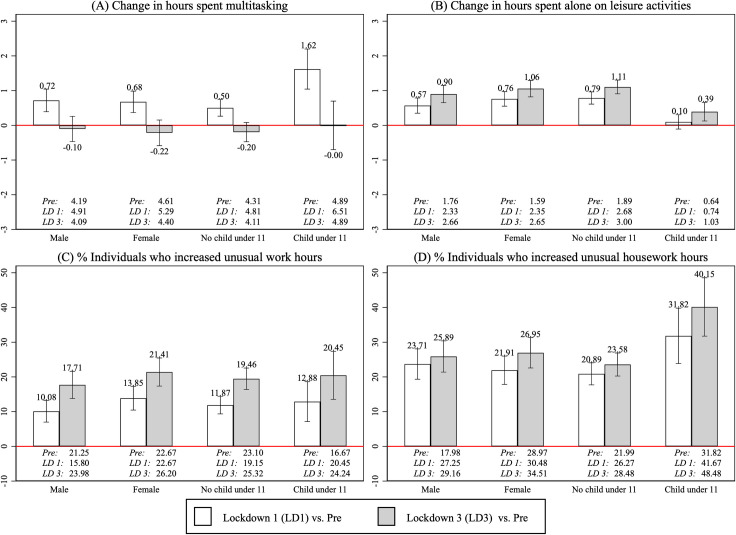
Within-person changes in quality of time use. Bars present average within-person changes in quality of time use between the pre-pandemic timepoint (February 2020) and the first lockdown (May 2020) or the third lockdown (March 2021). Unusual work includes the job searching activities of the unemployed. Error bars represent 95% confidence intervals, and average levels for each subgroup are reported underneath the bars. Note that the conditional means were calculated separately (either by gender or household composition), so the four subgroups shown are not mutually exclusive.

Fig 3 shows average within-person differences for these 4 measures. While the significant increase in hours spent multitasking only occurred during the first lockdown (Fig 3A), the increase in time spent doing leisure activities alone was larger in the third lockdown, even for individuals with young children (Fig 3B).

The pandemic also had substantial effects on patterns of time use, disrupting typical workday routines and blurring the distinction between work and family life. Compared to the pre-pandemic timepoint, Fig 3C shows that there was a significant increase in the proportion of individuals who worked unusual hours (outside 8.30–17.30 on a workday). In the third lockdown, 18% (95% CI = [14, 22]) of males saw an increase in time spent on work-related activities during unusual hours. Fig 3D shows that individuals living with young children were disproportionately more likely to do housework during unusual hours (8.30–17.30 during a workday): in the third lockdown, 40% of individuals in this group (95% CI = [32, 49]) increased time spent on housework during typical working hours compared to 24% (95% CI = [20, 27]) of individuals without young children.

### Time use and overall enjoyment

These observed changes in the quality and quantity of time use could affect individuals’ experiences of conducting their daily activities. To investigate this possibility, we examined how self-reported enjoyment varies across timepoints. For each individual and timepoint, we calculated a single measure of enjoyment by aggregating episode-specific enjoyment (measured on a 1–7 Likert scale) across all episodes and diary days, weighted by the duration of time spent on each episode.

**Fig 4:**
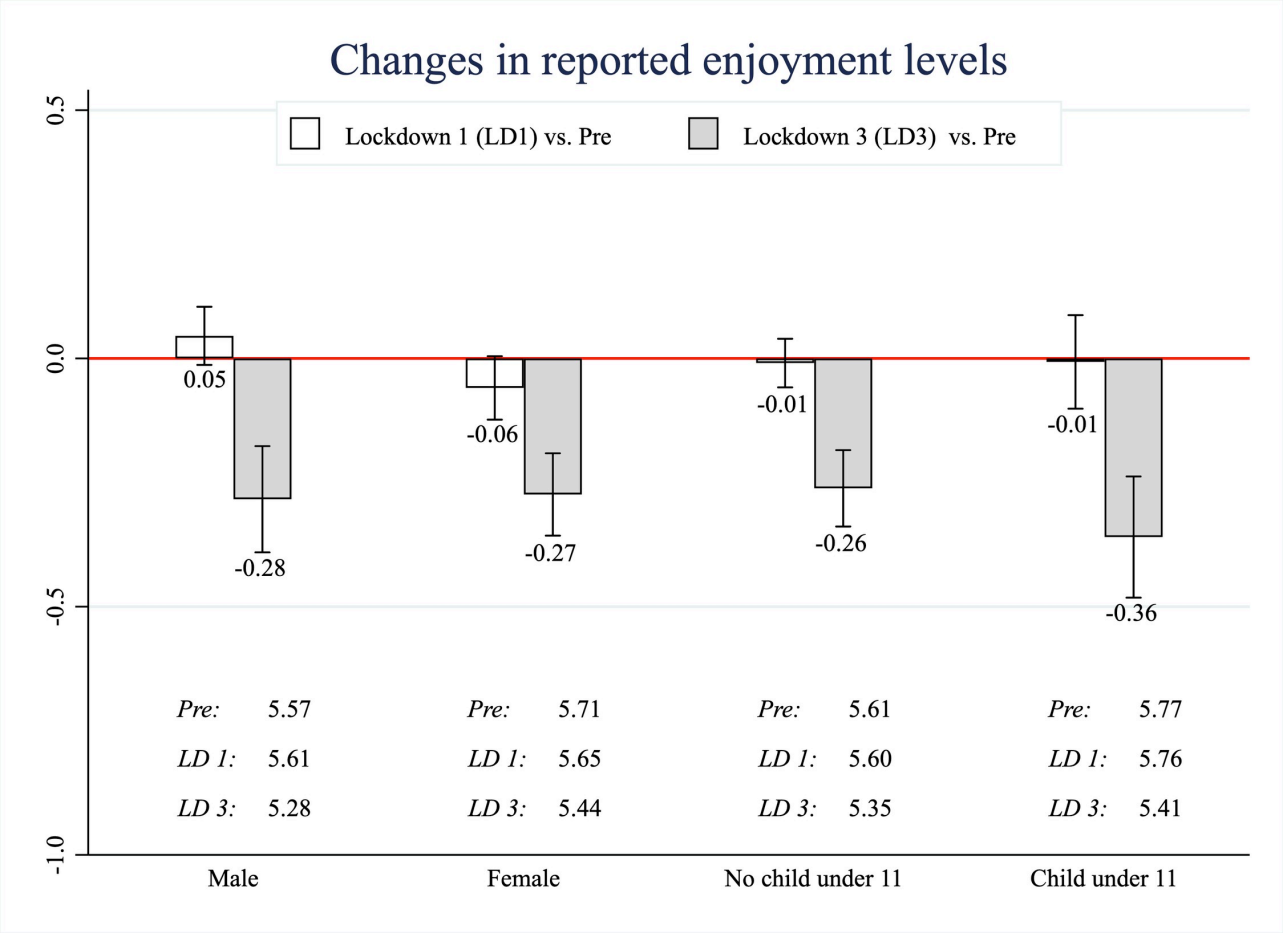
Changes in self-reported enjoyment. Bars present average within-person changes in self-reported enjoyment between the pre-pandemic timepoint (February 2020) and the first lockdown (May 2020) or the third lockdown (March 2021). Error bars represent 95% confidence intervals, and average levels for each subgroup are reported underneath the bars. 1 standard deviation corresponds to 0.76 units on the 1–7 enjoyment Likert scale in the pre-pandemic period. Note that the conditional means were calculated separately (either by gender or household composition), so the four subgroups shown are not mutually exclusive.

Fig 4 shows average within-person differences in overall enjoyment, comparing the first and third lockdown to the pre-pandemic timepoint. Calculating within-person differences of aggregate enjoyment helps mitigate issues with interpersonal comparability of levels of enjoyment [16]. Across all subgroups considered, overall self-reported enjoyment during the third lockdown was 0.26–0.36 points lower on a 1–7 scale relative to the pre-pandemic period (equivalently, 0.34–0.47 standard deviations lower, given that 1 standard-deviation corresponds to 0.76 units in the pre-pandemic period), with the largest average decrease among respondents living with young children during the third lockdown (-0.36 points, 95% CI = [-0.48, -0.24]). These findings are consistent with earlier studies on UK adults during the first lockdown, which found that the first lockdown adversely affected mental wellbeing [45–47].

To further examine the relationship between enjoyment, quality of time use, and quantity of time use, we regressed within-person changes in overall enjoyment on changes in quantity and quality measures of time use, measured in hours, controlling for sociodemographic characteristics. We used the following regression specification:

$$\Delta E_{i}=\alpha +\gamma^{\prime }W_{i}+\lambda^{\prime }{\Delta Q}_{i}+\varepsilon_{i}$$ (2)

Δ*E_i_* is within-person changes in overall enjoyment (either the first lockdown minus pre-pandemic level or third lockdown minus pre-pandemic level). ***W**_i_* is the same vector of respondent characteristics included in the time use regressions specified in Eq (1). Δ***Q**_i_* is a vector containing within-person changes in time spent (hours per day) on the four broad activity categories across two timepoints (e.g. the first lockdown minus pre-pandemic) and within-person changes (hours per day) in the four quality measures across the same two timepoints. Since the variable measuring changes in time spent on employment was missing for individuals who were unemployed at any given timepoint, we used the missing indicator method to include all respondents in the regression regardless of employment status, alleviating concerns about sample selection [48]. Specifically, we replaced the missing variable with some arbitrary fixed value and include in Eq (2) a binary indicator that equals 1 if that variable is missing and zero otherwise.

**Fig 5:**
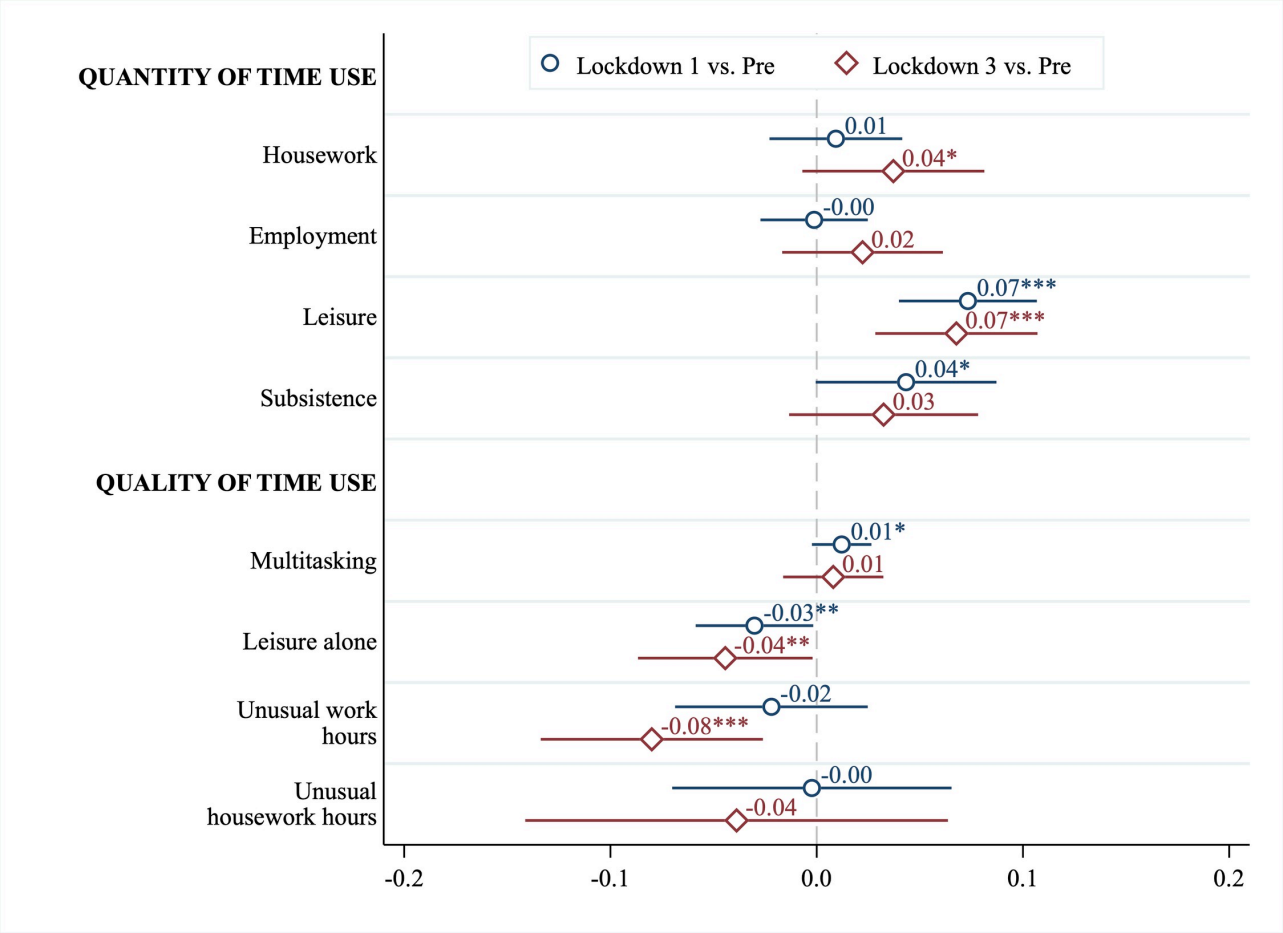
Relationship between changes in enjoyment, quantity of time use, and quality of time use. Estimates of correlations between within-person changes in overall self-reported enjoyment and characteristics of time use. Reported changes during the first and third lockdown (May 2020 and March 2021, respectively) are relative to the pre-pandemic timepoint (February 2020). Regressions include all individuals in our sample. In addition to the variables reported, we also controlled for a vector of respondent characteristics (see main text), and a binary indicator if changes in time spent on employment was missing. A coefficient of 0.1 corresponds to ~0.13 SD in pre-pandemic enjoyment levels. Point estimates are reported with 95% confidence intervals, using robust standard errors. *** p < 0.01, ** p < 0.05, * p < 0.1.

The estimated coefficients of Eq (2), shown in Fig 5, suggest that changes in characteristics of time use (quality) were significantly correlated with changes in overall enjoyment. A one-hour increase in leisure time during the first lockdown was associated with a 0.07 unit (0.09 standard deviation) increase in overall enjoyment (*p* < 0.01), but this effect was reduced by 0.03 units (0.04 standard deviations) if leisure time was spent alone. We obtained similar results for the third lockdown: compared to the pre-pandemic timepoint, a one-hour increase in leisure time spent alone was associated with a 0.04 unit (0.05 standard deviation) decrease in overall enjoyment (*p* < 0.05). Furthermore, individuals who worked an extra hour outside of typical working hours during the third lockdown experienced a 0.08 unit (0.11 standard deviation) decrease in overall enjoyment compared to the pre-pandemic period (*p* < 0.01). Full regression tables are presented in Section 6.6 in S1 Appendix.

### Robustness of results

Our main analysis relied on the longitudinal nature of our data. To account for potential bias arising from attrition between Waves 1 and 2, we used inverse probability weights to re-weight our longitudinal sample. Specifically, we ran a probit regression where the outcome variable equals 1 for respondents that participated in both waves and 0 otherwise, and the control variables are age categories, a binary indicator for female, a binary indicator for white ethnicity, a binary indicator for having a tertiary degree, and a binary indicator for living with a child under 11. Using the estimated coefficients, we then predicted the probability of appearing in both survey waves and used the inverse of these predicted probabilities as weights. Section 7 in S1 Appendix shows that we obtained qualitatively similar results when using weights to correct for potential attrition bias.

To assess the representativeness of our results, we reweighted our sample to match the composition of Understanding Society’s in-workforce sample across gender, age, ethnicity, education, and household composition (all defined as categorical variables). Section 8 in S1 Appendix contains further details about the construction of these calibration weights and shows that our results remain qualitatively similar when these weights are applied.

Lastly, in Section 9 in S1 Appendix, we argue that our results are unlikely to be confounded by sample selection based on unobservable characteristics or by differential measurement error in time use across timepoints.

## Discussion

The pandemic-induced national lockdowns caused drastic changes in the daily routines of many individuals. Aside from changing the total allocation of time across various activities (‘quantity’), these national lockdowns may also have changed the way these activities are conducted (‘quality’), which could affect individuals’ enjoyment of their time in the short run and mental health outcomes in the long run.

Our study investigated this issue using unique longitudinal data on a demographically diverse group of UK adults, comparing three timepoints: pre-pandemic (February 2020), the first national lockdown (May 2020), and the third national lockdown (March 2021). For both lockdowns, we documented significant changes in the quantity of time use: compared to the pre-pandemic timepoint, individuals who remained employed in the first or third lockdown spent less time on employment-related activities and more time on housework, with the effects being concentrated on individuals with young children. Females with young children were especially disadvantaged as they were less likely to remain in employment during either lockdown. Our comparisons of the first and third lockdowns highlight the similar nature of changes in time use and complement existing literature on the first lockdown [49].

We also found clear evidence that the quality of time use decreased during both lockdowns, with increases in leisure time spent alone and a larger proportion of individuals working unusual hours and conducting housework during working hours. These changes in quality of time use are important for self-reported enjoyment. For example, an increase in time spent on employment activities conducted outside normal working hours was negatively associated with overall enjoyment.

The observed reduction in leisure and increase in housework are likely to be reversed as the UK resumes large-scale social and cultural events and schools return to normal operations, but the effects of COVID-19 on working arrangements are likely to persist [1, 50, 51]. As new variants threaten the efficacy of vaccines, social distancing restrictions and national lockdowns may still be implemented in the future [52, 53]. Therefore, our results provide useful insights for pandemic-related policymaking.

While changes in quantity of time use and the resulting inequality across population subgroups have been well-documented in the literature, the additional adverse effects through changes in quality, particularly the timing of activities, is an important yet understudied policy concern. Given that ‘hybrid working’ (splitting time between the office and home) is likely to become part of normal working practices [51], our findings suggest that company policies aimed at promoting work-life balance for teleworkers, such as limits on email communications after working hours, could improve wellbeing and prevent long-term mental health issues. Employers should design home-working schedules that support the needs of already-disadvantaged demographic subgroups, such as households with young children. Further research is needed to assess specific initiatives that address the long-term consequences of the pandemic on quality of time use and wellbeing.

## Supporting information

S1 Appendix Online supporting materials. (PDF)

S2 Appendix Survey. (PDF)

S1 File Replication code. (ZIP)
