# Supplementary material for: Changes in the quantity and quality of time use during the COVID-19 lockdowns in the UK: Who is the most affected?
Source: PLoS One. 2021 Nov 3;16(11):e0258917. doi: 10.1371/journal.pone.0258917 (PMC8565783; doi:10.1371/journal.pone.0258917)
Supplement: S1 Appendix — (PDF) [file pone.0258917.s001.pdf]

S1 Appendix for

Changes in the quantity and quality of time-use during the COVID-19 lockdowns in the UK: Who is the most affected?

Ines Lee Eileen Tipoe

This document contains:

1 Study context 2
2 Data collection 2
3 Data description and cleaning 6
4 Main variables 12
5 Pre-analysis plan 13
6 Additional Results 14
7 Robustness to attrition 29
8 Sample representativeness 41
9 Other data quality issues 54
References 55

Data and code have been submitted as supplementary material.

Our pre-registered plan for the analysis is available at: https://aspredicted.org/blind.php?x=3az7we

1 Study context

To slow down the spread of COVID-19, the UK underwent a three-month national lockdown from 26 March to 23 June 2020 (‘first national lockdown’). The lockdown measures resulted in drastic changes to most daily routines: all schools and ‘non-essential’ shops were closed, UK residents were not allowed to leave their home except for a few specific reasons (such as buying necessary supplies), and anyone who was not classified as a ‘key worker’ (such as NHS staff) was instructed to work from home.

Similar restrictions were in place during UK’s third national lockdown, lasting from 6 January to 12 June 2021. Table S1 compares key public health measures implemented during both lockdowns (at the time of our survey), using Oxford’s COVID-19 Government Response Tracker data [1]. The key difference between the first and third lockdowns is that stay-at-home requirements were recommended during the first lockdown, but required during the third lockdown.

Note that the UK’s second national lockdown, from 5 November to 2 December 2020, was substantially less restrictive, with schools and non-essential shops remaining open, and no stay-at-home or work-from-home mandate. Using COVID-19 Government Response Tracker data [1] to put these differences in perspective, the average stringency of measures during the first and third lockdowns was 77.2 and 84.8 on a 0-100 scale respectively,[1] compared to 67.3 during the second lockdown. We therefore focused our study on the first and third national lockdowns.

**Public health measure**	**First lockdown**	**Third lockdown**
School closures	Required (all levels)	Required (all levels)
Workplace closures	Required for all except key workers	Required for all except key workers
Stay-at-home requirements	Recommended	Required (except essentials)
Restrictions on public gatherings	< 10 people	< 10 people
Restrictions on internal movement	Restricted movement	Restricted movement

Table S1. Comparison of key public health measures during the first and third lockdown, using data and classifications from the COVID-19 Government Response Tracker. Measures correspond to the weeks in which we conducted our survey.

2 Data collection

We collected data in two waves. All respondents were paid a modest incentive of 5 GBP per hour for their participation.

2.1 Wave 1

Wave 1 was conducted in 13-19 May 2020, 7 weeks into the UK’s first national lockdown. In this wave, we collected sociodemographic information and time use diaries for the first two timepoints: pre-pandemic (defined as February 2020) and the first national lockdown (26 March to 23 June 2020).

We used the survey platform Prolific to recruit individuals who were over 18, had lived in the UK since December 2019, and were still in the labor market (including unemployed and searching for work) in February 2020. Prolific is a reputable survey company used primarily by researchers for surveys and experiments. Compared to in-person data collection methods or similar platforms such as MTurk, Prolific has been shown to deliver higher or comparable data quality [2][3].

To ensure we recruited a demographically diverse sample and improve the generalizability of our results, we requested that our sample should match the composition of the UK population in gender, age, and ethnicity (see Prolific for further details of their recruitment process and criteria used: https://researcher-help.prolific.co/hc/en-gb/articles/360019236753-Representative-Samples-on-Prolific).

[1] Averages were taken from the start of the national lockdown until the end of the month in which we conduct the survey wave (26 March – 30 May 2020 for the first lockdown, 6 January – 31 March 2021 for the third lockdown).

Prolific contacted a total of 1239 individuals. 1060 of these individuals (85.6%) submitted a complete response to the Wave 1 questionnaire, 42 individuals (3.4%) submitted unusable responses (such as incomplete or missing time use diaries), and 137 individuals (11.1%) did not respond.

2.2 Wave 2

We contacted the same respondents in 1-7 March 2021 (Wave 2), 7 weeks into the UK's third national lockdown. We collected time use diaries for this timepoint as well as information about changes in respondents' employment situation.

Of the 1036 respondents from Wave 1, 762 filled in the Wave 2 questionnaire, giving a response rate of 74%. Table S2 compares our longitudinal sample to the full sample and shows that at the 5% level, the observable characteristics of Wave 1 and Wave 2 respondents were similar.

Covariate	**Percentage** (Wave 1)	**Percentage** (Wave 2)	**Difference** (Wave 1 – Wave 2)
Age 18-24	9.92	7.70	2.21
Age 25-29	11.71	10.05	1.66
Age 30-34	12.09	9.92	2.17
Age 35-39	7.55	6.92	0.64
Age 40-44	12.46	13.05	-0.59
Age 45-49	6.89	7.44	-0.55
Age 50-54	9.35	10.84	-1.49
Age 55-59	7.18	7.05	0.13
Age 60 or above	22.85	27.02	-4.17**
Female	50.76	52.35	-1.59
White	84.51	84.84	-0.32
Tertiary degree	60.72	61.62	-0.90
Has young child	18.04	16.19	1.85
Number of respondents	1059	766.	

Table S2. Comparison of Wave 1 and Wave 2 respondents. Covariates were defined as binary variables that equal 1 if the respondent satisfied the specified condition. 'White' includes mixed-race respondents. 'Has young child' equals 1 if the respondent lives with at least one child aged 11 or under. 'Tertiary degree' equals 1 if the respondent obtained any post-secondary educational qualification. *** $p < 0.01$, ** $p < 0.05$, * $p < 0.1$.

To conduct a more formal check for attrition bias, we ran a probit regression where the outcome variable equals 1 for respondents that participated in both waves, and 0 otherwise, using the baseline characteristics reported in Table S2 as covariates. Estimates are reported in Table S3. Older respondents were significantly more likely to participate in both waves, but we did not find evidence of systematic variations in participation across gender, ethnicity, education, or household composition. As a robustness check, we repeated our main analysis using inverse probability weights to account for attrition among younger respondents and obtained qualitatively similar results (Section 7).

	**Participated in both waves**		
	(1)	(2)	(3)
Age 25-29	0.09	0.08	0.09
	(0.17)	(0.17)	(0.17)
Age 30-34	0.06	0.07	0.09
	(0.17)	(0.17)	(0.17)
Age 35-39	0.34*	0.36*	0.39*
	(0.19)	(0.19)	(0.20)
Age 40-44	0.52***	0.52***	0.55***
	(0.17)	(0.17)	(0.18)
Age 45-49	0.74***	0.75***	0.76***
	(0.21)	(0.21)	(0.21)
Age 50-54	0.81***	0.82***	0.83***
	(0.19)	(0.20)	(0.20)
Age 55-59	0.58***	0.59***	0.59***
	(0.20)	(0.20)	(0.20)
Age 60 or above	0.71***	0.73***	0.73***
	(0.15)	(0.16)	(0.16)
Female		0.10	0.10
		(0.08)	(0.08)
White		-0.06	-0.06
		(0.12)	(0.12)
Tertiary degree		0.04	0.04
		(0.09)	(0.09)
Has young child			-0.07
			(0.12)
Pseudo-R^2	0.04	0.04	0.04
Observations	1059	1056	1056

Table S3. Estimates of correlations between sociodemographic characteristics and participation in both survey waves. A positive coefficient indicates the covariate was associated with an increased probability of participating in both waves. 'White' includes mixed-race respondents. 'Has young child' equals 1 if the respondent lives with at least one child aged 11 or under. 'Tertiary education degree' equals 1 if the respondent obtained any post-secondary educational qualification. Robust standard errors are reported in brackets. *** p < 0.01, ** p < 0.05, * p < 0.1.

2.3 Longitudinal sample

Our longitudinal sample consisted of individuals who completed at least one time use diary for each timepoint (N=766). Table S4 shows descriptive statistics comparing characteristics of our respondents with those participating in a nationally representative longitudinal survey (Understanding Society). As our sample only included adults who were in the workforce from January-May 2020 (including those unemployed but looking for a job), for comparability we also present characteristics of the correspondent subset from Understanding Society. Compared to Understanding Society, our sample contained similar proportions of respondents in some age brackets (aged 25-29, 30-34, 40-44, 50-54) and those who identified as white.

Since our sample overrepresented adults with a tertiary degree and older adults, there may be concerns that our results are specific to the group of individuals we recruited. As a robustness check, in Section 8, we repeated our main analysis using calibration weights so that our sample was reweighted to match the weighted Understanding Society in-workforce characteristics reported in Table S4 (gender, age, ethnicity, education, and household composition). We obtained qualitatively similar findings, suggesting that our results do not seem to be specific to our particular sample composition.

	Our data	Understanding Society (All adults)		Understanding Society (Aged 18-86, in the workforce)	
		Unweighted	Weighted	Unweighted	Weighted
Age 18-24	0.08	0.11	0.12	0.09	0.11
Age 25-29	0.10	0.08	0.08	0.11	0.11
Age 30-34	0.10	0.09	0.08	0.12	0.11
Age 35-39	0.07	0.10	0.09	0.13	0.12
Age 40-44	0.13	0.10	0.09	0.14	0.13
Age 45-49	0.08	0.09	0.09	0.13	0.13
Age 50-54	0.11	0.08	0.08	0.11	0.11
Age 55-59	0.08	0.07	0.07	0.09	0.09
Age 60 and above	0.25	0.25	0.27	0.08	0.08
Female	0.52	0.55	0.51	0.50	0.47
White	0.85	0.78	0.88	0.79	0.88
Tertiary degree	0.60	0.31	0.30	0.41	0.40
Has young child	0.17	0.28	0.24	0.32	0.30
Observations	766	50875	50881	27503	27506

Table S4. Comparison of respondents in our data and in Understanding Society, a nationally representative survey of the UK population. Proportions from Understanding Society were calculated using the first wave, which took place in 2009-2010. Weighted proportions and totals were calculated using the individual weights provided by Understanding Society. Covariates were defined as binary variables that equal 1 if the respondent satisfied the specified condition. 'White' includes mixed-race respondents. 'Has young child' equals 1 if the respondent lives with at least one child aged 11 or under. 'Tertiary degree' equals 1 if the respondent obtained any post-secondary educational qualification.

3 Data description and cleaning

3.1 Time use diaries

Our survey consisted of two parts: time use diaries and sociodemographic information.

For each timepoint (pre-pandemic, first lockdown, third lockdown), respondents retrospectively filled in time use diaries for their most recent workday (if applicable) and most recent non-workday. Diaries for the first two timepoints were completed in one sitting (Wave 1), and diaries for the third timepoint were completed in another sitting (Wave 2).

Time use diaries record the chronological sequence of activities that respondents did over a 24-hour period through a series of 'episodes'. The 24-hour period we chose was midnight to 11:59pm. The time use diary format and structure followed that of the 2014/15 UK Time Use Survey (https://www.timeuse.org/node/10833). For each episode within a time use diary, we asked respondents to fill out: (1) the episode start and end time (with a minimum duration of 10 minutes per episode); (2) the main activity of that episode; (3) the secondary activity that the respondent was engaged in simultaneously (if any); (4) whom they did the activity with; (5) where they did the activity; (6) whether they used a device for that episode; (7) how much they enjoyed the activity (on an increasing scale of 1 to 7). Figure S5 shows a screenshot of the format used to record episode-specific information.

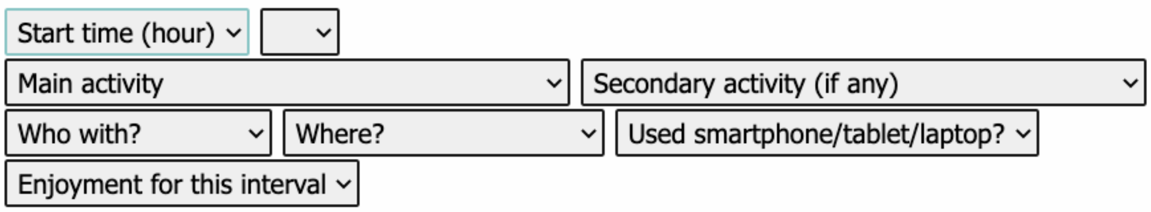

Figure S5. Screenshot of one time use 'episode'. Clicking on the arrows leads to dropdown menus with pre-specified options. For the full list of options, see Table S5.

For the main and secondary activity, respondents chose from a pre-defined list of 42 activities (derived from the 2014/15 UK Time Use Survey), each falling under one of 12 broad activity domains. For our main analyses, we further aggregated these activities into 4 categories: Housework, Employment, Leisure, and Subsistence (40 categories total, excluding travelling and studying). Table S6 shows the mapping between individual activities, activity domains, and categories.

To ensure that respondents recorded information in a comparable way, before filling in their time use diaries, respondents were given written guidelines and examples of how to enter in episode-specific information. Respondents also had to correctly complete three fictional diary episodes based on specific information provided before they could proceed to their time use diaries.

Activity category	Activity domain	Specific activities
Housework	Household/Family care	Cooking
		Groceries
		Cleaning
		Childcare
		Caring for pets
		Other caring duties
		Bills/household accounts
		Gardening
		Repairs
		Other household activities
Employment	Employment-related	Work tasks
		Meetings
		Searching for jobs
		Casual work
		Tea/Coffee break
		Other employment-related activities
Leisure	Social/cultural	Communicating with others
		Watching movie at the cinema
		Attending a social gathering
		Shopping (non-groceries)
		Having visitors/visiting a friend
		Going to a museum/other cultural site
		Other social/cultural activities
	Arts/Hobbies	Playing games (any type)
		Doing crafts
		Doing art
		Singing
		Other arts/hobbies
	Mass media consumption	Watching TV
		Social media
		Listening to music
		Reading/watching/listening to the news
		Reading
		Other mass media consumption
	Physical exercise	Walking
		Other exercise
	Volunteering	Volunteering
Subsistence	Sleeping	Sleeping
	Eating	Eating
	Personal care	Personal care

Table S6. Time use diaries: Mapping between individual activities provided in the survey (based on the UKTUS), activity domains in the UKTUS, and broad activity categories used in our analysis. Table 1 in the main manuscript shows the mapping between broad activity categories and the subcategories used in our analysis.

3.2 Time use diary cleaning

This section describes specific issues that arose in the raw diary data and how we dealt with them.

Missing activities. There were some episodes that have missing main activities. If the main activity was missing and the secondary activity was not missing, we recoded the secondary activity as the main activity. If the episode was missing a main activity but not missing the start and end times, we checked if the subsequent or preceding rows had non-empty activities and missing or equivalent start or end times. If so, we replaced the missing activities (and other information for that episode) with the activities in the subsequent or preceding row. For example, if row n began at 16.00 and ended at 17.30, had a missing main activity and missing secondary activity and if row $n + 1$ had missing times but had cooking and eating recorded as the main and secondary activities respectively, we replaced row n with activities from row $n + 1$.

Missing start and end times. There were some episodes that had missing start or end times. If the episode was missing a start time, we replaced the start time with the end time of the preceding episode, or 24.00 if the episode was the last in that diary day. If the episode was missing an end time, we replaced the end time with the start time of the subsequent episode, or 0.00 if the episode was the first in that diary day. We dealt with entries that both started and ended at 0.00 or 24.00 in a later step. If episode n had a missing end time (but had a start time), and episode $n + 1$ had a missing start time (but had an end time), we set the duration of the episode to be 10 minutes.

Incorrectly recorded AM and PM times. We provided respondents with a 24-hour clock to record the start and end times of each episode. There were episodes where respondents mixed up the AM or PM nature of the clock. For example, an episode that spanned 14.00-15.15 may be followed by an episode that spanned 3.15-4.00. In these cases, we adjusted the times to be consistent with the sequence of activities reported by the respondent.

Overlapping episodes. Within each diary, there were consecutive episodes that overlap. This could happen in the following ways:

- Two episodes had the same start and end time. If the secondary activity was blank for both episodes, we combined the episodes into one single episode where the activity from the first entry was the main activity and the activity from the second entry became the secondary activity. If the secondary activity was non-blank for only one episode, we kept the episode with the secondary activity and used the other entry to impute any missing activity characteristics. If the secondary activity was non-blank for all overlapping episodes and the main activity was the same, we adjusted the end time of the first entry and the start time of the second entry so both episodes had equal duration. If the secondary activity was non-blank for all overlapping episodes and the main activities differed across overlapping episodes, we combined the overlapping episodes into one episode, where the main activity from the second episode became the secondary activity for the combined episode.
- Two episodes had the same start times but different end times (e.g. episode n spanned 15.00-16.00 while episode $n + 1$ spanned 15.00-16.30). If the overlapping episodes had the same main and secondary activity, we kept the episode that ended later. If the overlapping episodes did not have the same main and secondary activities, we kept these overlapping episodes, and in a later step we adjusted the end time of episode n and the start time of episode $n + 1$ by equal amounts so that they did not overlap. In the example above, the times would be adjusted to be 15.00-15.45 for episode n and 15.45-16.30 for episode $n + 1$.
- Two episodes had different start times but the same end time. (e.g. episode n spanned 15.00- 16.00 while episode $n + 1$ spanned 15.30-16.00). If the episodes had the same main and secondary activity, we kept the episode that started earlier. If the episodes did not have the same main and secondary activities, we kept these overlapping episodes, and in a later step we adjusted the end time of episode n and the start time of episode $n + 1$ by equal amounts so that they did not overlap. In the example above, the times would be adjusted to be 15.00-15.45 for episode n and 15.45-16.30 for episode $n + 1$.
- Two episodes had different start and end times but the time intervals of episode n and episode $n + 1$ overlapped (e.g. episode n spanned 15.00-16.00 while episode $n + 1$ spanned 15.30-16.30). If the overlapping episodes had the same main and secondary activity, we combined both entries into one episode. If the episodes had different activities, then we adjusted the end time of episode n and the start time of episode $n + 1$ by equal amounts so that they did not overlap. In the example above, the times would be adjusted to be 15.00-15.45 for episode n and 15.45-16.30 for episode $n + 1$).

Missing interval between 2 episodes. There were some episodes that had a positive time gap between them (e.g. episode n spanned 15.00-16.00 and episode $n + 1$ spanned 16.30-18.00). If the missing time interval between the end time of episode n and the start time of episode $n + 1$ was less than 60 mins, we adjusted the end time of episode n to

equal the start time of episode $n + 1$. If the missing time interval was over 60 minutes, then we adjusted the end time of the episode n and the start time of episode $n + 1$ to meet halfway.

Same start and end time. There were some episodes that began and ended at the same time. If an episode was not the first entry in a diary day but had the same start and end time, we adjusted the start time to be 10 minutes earlier and the end time of the preceding episode to be 10 minutes earlier. If an episode was not the last entry in a diary day but had the same start and end time, we adjusted the end time to be 10 minutes later and the start time of the subsequent episode to be 10 minutes later.

Imputing starting or ending sleep episodes. Some diaries did not start at 0.00 or end at 24.00 because respondents did not include their sleep episodes (e.g. the first entry of the day started at 7.00 with the main activity 'eating'). In these cases, we added an entry where the main activity was 'sleeping'. The same adjustment was made by the UKTUS. We also imputed the characteristics of these episodes as follows: secondary activity = none, where = at home, used a device = no, with whom and episode-specific enjoyment = modal answer across all recorded sleep episodes by that respondent for that timepoint.

Table S7 reports summary statistics for the time use diaries after cleaning. Only a very small proportion of episodes needed cleaning, and the proportion of cleaned episodes were similar across timepoints.

	**Pre-pandemic**	**First lockdown**	**Third lockdown**
	(February 2020)	(May 2020)	(March 2021)
Number of episodes per diary day	12.30	11.63	14.98
	(3.78)	(3.60)	(4.67)
Episode duration (hrs)	2.13	2.25	1.77
	(2.27)	(2.38)	(2.04)
Missing activities	0.00	0.00	0.00
	(0.02)	(0.02)	(0.02)
Missing start or end time	0.00	0.00	0.00
	(0.02)	(0.01)	(0.02)
Episodes with incorrect AM/PM	0.01	0.00	0.01
	(0.08)	(0.06)	(0.07)
Overlapping episodes	0.00	0.00	0.01
	(0.06)	(0.04)	(0.08)
Missing interval between episodes	0.09	0.07	0.07
	(0.28)	(0.25)	(0.25)
Imputed sleep episodes	0.05	0.04	0.02
	(0.29)	(0.25)	(0.16)
Number of episodes	15999	13308	17878
Number of diaries	1417	1247	1318

Table S7. Summary statistics for time use diary episodes after cleaning, by timepoint. Means were rounded to two decimal places, with standard deviations in brackets.

3.3 Summary statistics of time use diaries

As outlined in our pre-analysis plan, only individuals with at least one complete time use diary in each timepoint were counted in our final sample. A complete time use diary is defined as having 3 or more entries in one day (after data cleaning).

We obtained a total of 3982 time use diaries: 1417 for the pre-pandemic timepoint, 1247 for the first national lockdown, and 1318 for the third national lockdown. Since the time use diaries were completed retrospectively, and the pre-pandemic information was based on recall from three months earlier (respondents completed information on February 2020 in a May 2020 survey), there may be concerns that recall bias would particularly affect the pre-pandemic data.

To investigate this possibility, we compared the mean pre-pandemic time spent on each activity domain (specified in Table S6) with the mean times obtained from a nationally representative survey (the 2014/15 UK Time Use Survey). This approach followed that of other COVID-19 studies on time use, which used UK data from 2014-2016 as the pre-pandemic baseline [4–6].

Table S8 shows the mean time spent (hours per day) in each broad activity category used for our main analysis. Since the UKTUS was designed to be nationally representative, to make valid comparisons we re-weighted our data using calibration weights to match the composition of the Understanding Society in-workforce sample (see Section 8 for details of the methodology). Comparing our weighted data with the UKTUS in-workforce weighted data (columns in bold), the means across all broad activity categories were similar for both workdays and non-workdays ('Non-WD'). Therefore, we could proceed with some confidence in the reliability and external validity of our pre-pandemic baseline data.

	**Our data** (unweighted)		**Our data** (weighted)		**UKTUS, in workforce** (unweighted)		**UKTUS, in workforce** (weighted)	
	Workday	Non-WD	Workday	Non-WD	Workday	Non-WD	Workday	Non-WD
Employment	7.27	0.61	**7.22**	**0.59**	7.35	0.40	**7.45**	**0.49**
Housework	1.76	4.10	**1.91**	**4.40**	1.68	3.99	**1.59**	**3.94**
Leisure	3.03	6.56	**2.98**	**6.29**	3.16	6.13	**3.13**	**6.06**
Subsistence	10.58	11.96	**10.53**	**11.98**	9.72	11.62	**9.73**	**11.59**

Table S8. Comparison of mean time spent (hours per day) on broad activity categories during workdays and non-workdays (Non-WD) in our data and the 2014/15 UK Time Use Survey (UKTUS). 'In workforce' is the subset of UKTUS respondents who were employed or seeking work. Time spent across specific activities was aggregated into broad activity categories according to the classification in Table S6. Our data was re-weighted using calibration weights to match the composition of a nationally representative sample (Understanding Society in-workforce respondents). The UKTUS data was weighted to account for non-response, using weights provided by the UKTUS.

4 Main variables

4.1 Aggregate estimates of time spent in activity categories

For each diary day, we calculated the total time spent in each activity category by adding up time spent across specific activities (main activity only) using the classification in Table S6. Since a respondent completed up to 2 diary days per timepoint, we then obtained a single value for each timepoint by dividing the total time spent by the total number of applicable diary days: total time spent on housework, leisure, and subsistence were divided by 2 if a respondent completed both a workday diary and non-workday diary; total time spent on employment was not divided by 2 because there is at most one applicable diary day per timepoint. Note that the total time spent across these 4 categories may add up to less than 24 hours because we excluded travelling and studying from our main analysis.

4.2 Quality of time use

We used episode-specific information to construct 4 indicators for the quality of time use:

- Multitasking: For each respondent and timepoint, we calculated the total time spent on episodes that contained both a main and secondary activity, where the main and secondary activities belonged to different broad categories (e.g. employment as main activity, housework as secondary activity).
- Leisure time spent alone: For each respondent and timepoint, we calculated the total time spent on episodes where the activity category was 'leisure' and the episode-specific characteristic 'with whom' was 'alone' by the total number of episodes (leisure and non-leisure episodes).
- Increase in unusual work hours: Unusual work hours was defined as any employment-related activity conducted outside standard working hours (the time window of 8.30-17.30 on a workday), which included employment-related activities conducted on a non-workday and job searching activities undertaken by unemployed respondents. The time window was determined by taking the median start and end time of employment activities across all respondents' pre-pandemic workday diaries.
- Increase in unusual housework hours: Unusual housework hours was defined as any housework-related activity conducted within standard working hours (8.30-17.30 on a workday).

4.3 Enjoyment

For each timepoint, we calculated a single measure of enjoyment by aggregating episode-specific enjoyment (measured on a 1-7 Likert scale) across all episodes and diary days, weighted by the duration of time spent on each episode. To mitigate issues with interpersonal comparability of levels of enjoyment [7], we instead calculated within-person differences in aggregate ('overall') enjoyment over the timepoints considered.

4.4 Covariates

All regressions included the following covariates, which we also obtained via our online survey:

- **Female:** A binary variable that equals 1 if the respondent identified as female.
- **Living with child under 11:** A binary variable that equals 1 if the respondent reported living with at least one child aged 11 or under.
- **Working from home:** A binary variable that equals 1 if the respondent reported a non-zero percentage of time spent working from home during the period considered. We included two work-from-home indicator variables, corresponding to the first national lockdown (26 March 2020 – 23 June 2020) and third national lockdown (6 January 2021 – 12 June 2021) respectively.
- **Education:** A binary variable that equals 1 if the respondent's highest educational attainment was a postsecondary degree, which included 2-year postsecondary qualifications.
- **Age:** A set of binary variables indicating the respondent's age in May 2020: 18-24, 25-29, 30-34, 35-39, 40-44, 45-49, 50-54, 55-59, and 60 or older.
- **White:** A binary variable that equals 1 if the respondent identified their ethnic group as white (including mixed-race respondents).
- **Income:** Self-reported monthly personal before-tax income (in GBP) from all sources of employment (main job plus any secondary jobs), excluding income from other sources such as government benefits or investments. Respondents selected from 12 pre-defined categories, specified in intervals of 500 GBP, ranging from '0 GBP' to 'more than 5000 GBP'. We construct 5 binary variables corresponding to incomes of 1000-2000 GBP, 2000-3000 GBP, 3000-4000 GBP, 4000-5000 GBP and more than 5000 GBP.

In our regression analysis, we used values of these covariates from the pre-pandemic timepoint, with the exception of 'Working from home', which we allowed to vary across timepoints. The estimated coefficients on sociodemographic characteristics should therefore be interpreted as the correlation between the outcome variable and having that characteristic in February 2020.

5 Pre-analysis plan

Before conducting our analysis, we uploaded a pre-analysis plan to AsPredicted.org (https://aspredicted.org/blind.php?x=3az7we), which describes the key variables, sample inclusion criteria, hypotheses to be tested, and analyses to be conducted. Our analysis followed the procedures outlined in our pre-analysis plan, but with the following extensions:

- We initially planned to use fragmentation (number of times the respondent did the activity in a given day, divided by the total number of activities in that day) as an indicator for the quality of time use. However, since most respondents only repeated a given activity 1-3 times per day (defined according to our broad activity categories), this measure did not have a large enough range to yield meaningful descriptions of changes in the quality of time use. We therefore excluded fragmentation from our analysis and included two additional indicators (unusual work hours and unusual housework hours) that had strong justification in the literature.
- We initially planned to include episode-specific enjoyment (measured on a 1-7 Likert scale) as a control variable in multivariate regression analysis. However, after early presentations of our work and discussions with colleagues, questions were frequently asked about the effects of changes in quality and quantity on overall enjoyment, so we extended our analysis to include regressions with enjoyment as the dependent variable.

6 Additional Results

6.1 Changes in time use using alternative measures of time spent

Our main measure of total time spent was calculated using information about the main activity that the respondent engaged in during each episode. However, respondents could also record a secondary activity for each episode, so our measure does not capture any changes due to multitasking. We therefore considered two alternative measures: time spent as a secondary activity, and time spent as either the main or secondary activity.

Figures S9-S10 show bar charts analogous to Figure 1 in our main manuscript, using the alternative measures described above. Respondents with young children spent more time on housework as a secondary activity pre-pandemic, and significantly increased the time spent on housework as a secondary activity during both lockdowns. When considering time spent on each activity as a main or secondary activity, we obtained qualitatively similar results as in our main manuscript, but with larger magnitudes. For example, during the first lockdown, respondents with young children spent an average of 2.18 more hours per day on housework as a main or secondary activity, compared to 0.97 more hours per day when counting main activities only, though both of these changes are significant at the 5% level.

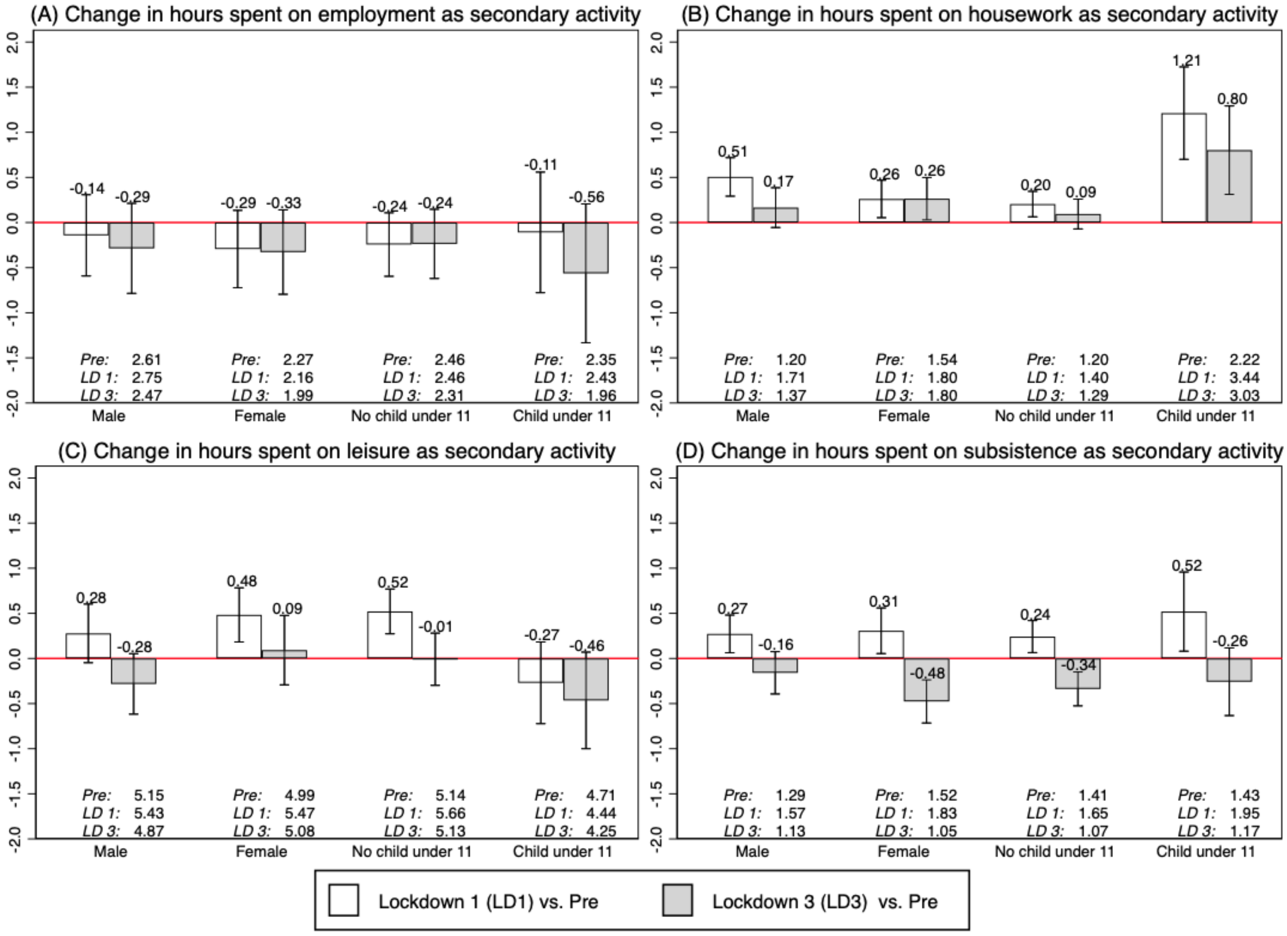

Figure S9. Average within-person changes in time spent on 4 broad activity categories, as a secondary activity only. Bars represent changes in hours per day spent on that category as a main activity, comparing the pre-pandemic timepoint (February 2020) to the first and third lockdowns (May 2020 and March 2021, respectively). Within-person changes for employment activities are calculated using the subset of individuals who remained employed in both periods of interest. Error bars represent 95% confidence intervals, and average levels for each subgroup are reported underneath the bars. Note that the conditional means were calculated separately (either by gender or household composition), so the four subgroups shown are not mutually exclusive.

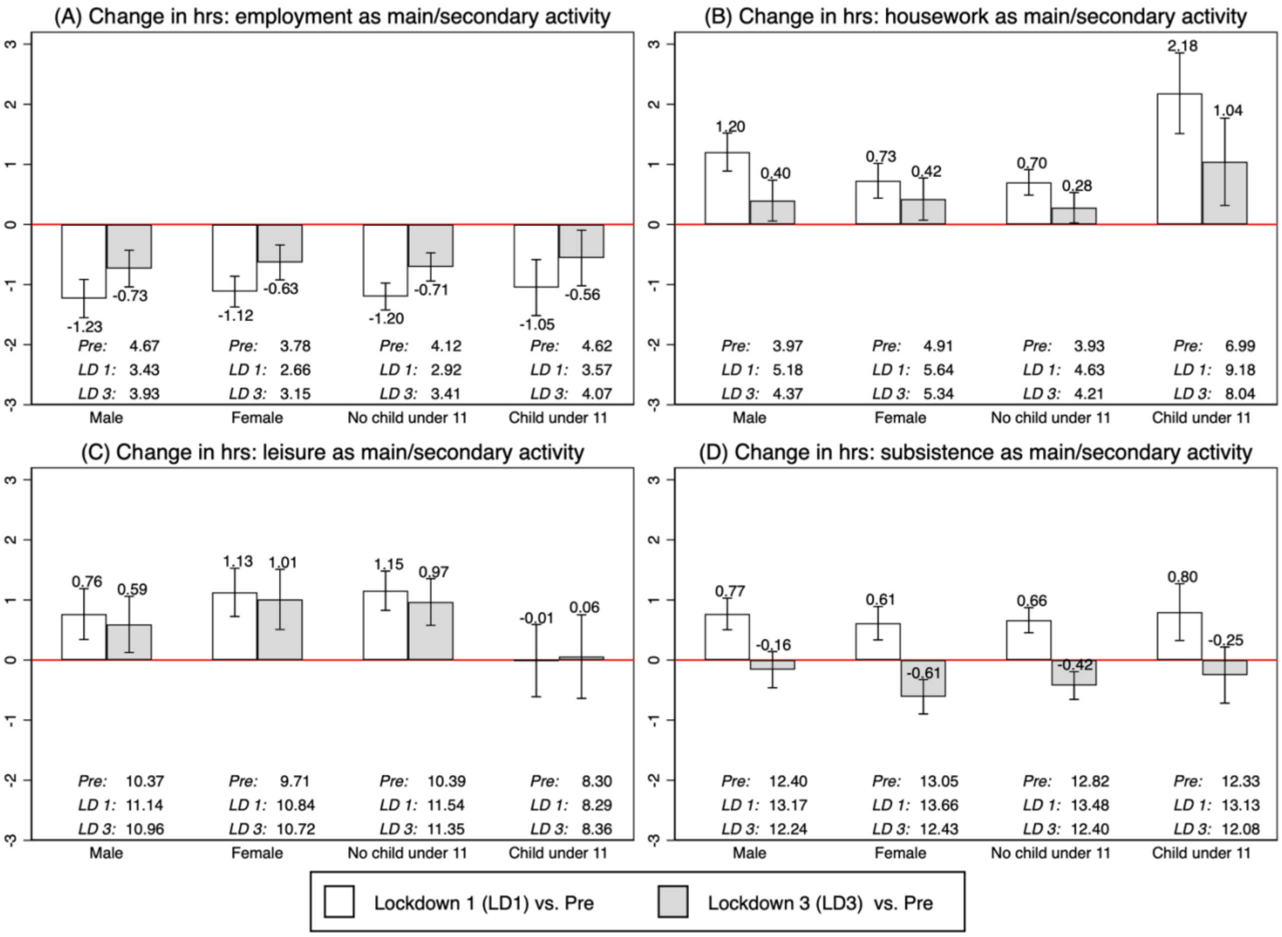

Figure S10. Average within-person changes in time spent on 4 broad activity categories, as either a main or secondary activity. Bars represent changes in hours per day spent on that category as a main activity, comparing the pre-pandemic timepoint (February 2020) to the first and third lockdowns (May 2020 and March 2021, respectively). Within-person changes for employment activities were calculated using the subset of individuals who remained employed in both periods of interest. Error bars represent 95% confidence intervals, and average levels for each subgroup are reported underneath the bars. Note that the conditional means were calculated separately (either by gender or household composition), so the four subgroups shown are not mutually exclusive.

6.2 Employment patterns

Table S11 shows the proportion of respondents (out of N=766) who were employed at each timepoint and at any two given timepoints. 86.04%, 62.97%, and 74.02% of respondents were employed during the pre-pandemic period, Lockdown 1, and Lockdown 3, respectively. 62.01% of respondents were employed in the pre-pandemic period and during Lockdown 1. 69.97% of respondents were employed in the pre-pandemic period and during Lockdown 3. 58.22% of respondents were employed during Lockdowns 1 and 3. 57.1% of respondents were employed across all three timepoints.

	**Pre**	**Lockdown 1**	**Lockdown 3**
**Pre**		62.01%	69.97%
**Lockdown 1**			58.22%
**Lockdown 3**			
	86.03%	63.97%	74.02%

Table S11. Employment probabilities for three timepoints: pre-pandemic (Pre), May 2020 (Lockdown 1), and March 2021 (Lockdown 3).

6.3 Measuring inequality in time use

As part of our pre-analysis plan, we intended to use Lorenz curves to visualize changes in the inequality of time use across respondents. Due to space constraints in the main manuscript, we present these results here (Figure S12).

Table S13 presents Gini coefficients of the distributions shown in Figure S12. Across broad activity categories, the distribution of time spent on housework as a main activity was the most unequal, and the distribution of time spent on subsistence activities was the most equal. Within timepoints, the larger Gini coefficients for time spent on secondary activities suggest variations in multitasking behavior. Across timepoints, the changes in time spent on employment are clearly seen in Figure S12: a substantial increase in inequality during the first lockdown that was partly reversed during the third lockdown.

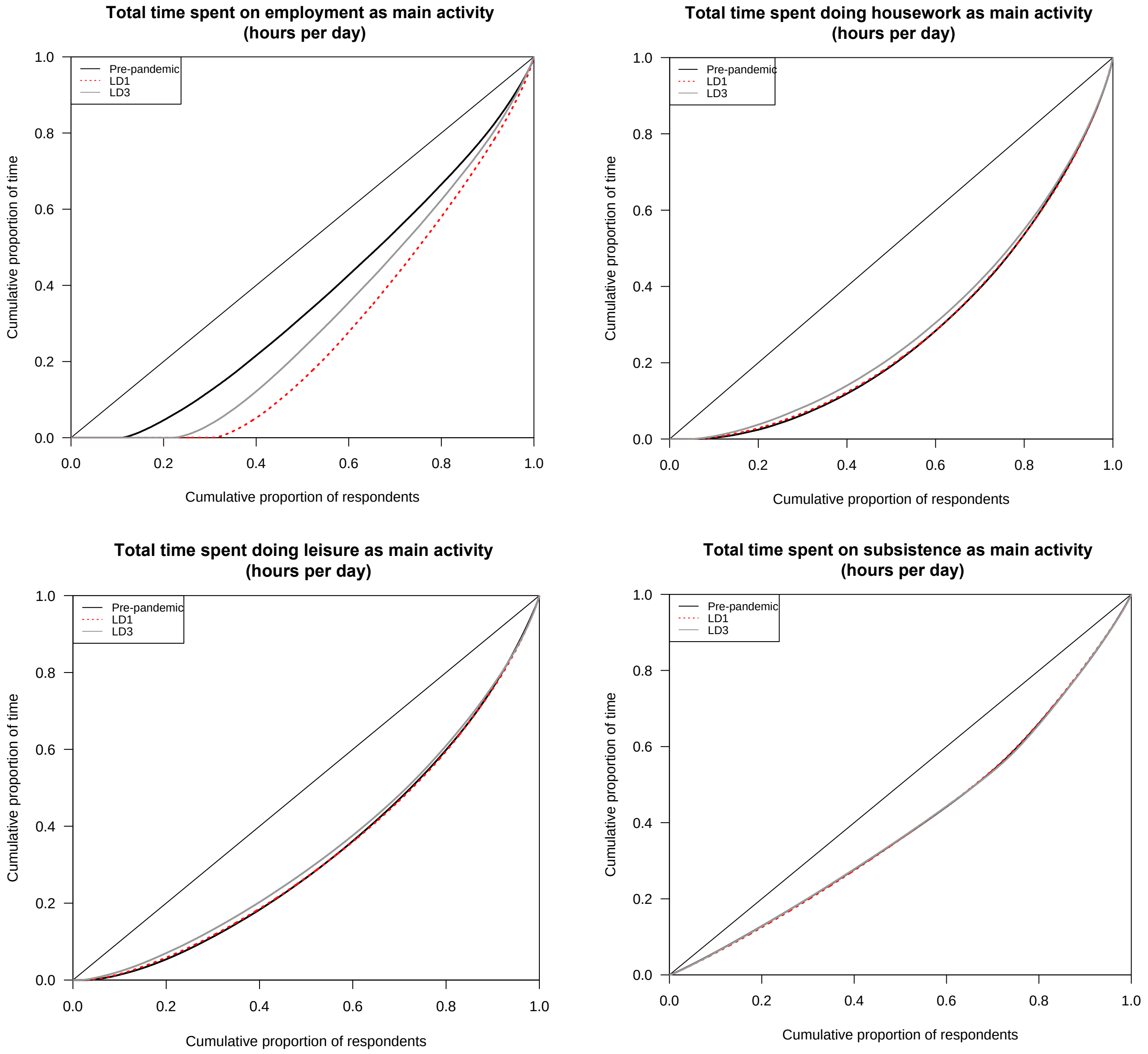

Figure S12. Lorenz curves of time spent (hours per day) on broad activity categories as a main activity, by timepoint. Curves that are further away from the 45-degree line (representing perfect equality) indicate a more unequal distribution of time spent. ‘Pre-pandemic’ refers to February 2020, ‘LD1’ refers to the first national lockdown (May 2020), and ‘LD3’ refers to the third national lockdown (March 2021). All respondents in our sample were included.

	Measure of time spent		
	Main	Secondary	Main and Secondary
**Employment**			
Pre	0.277	0.725	0.336
LD1	0.458	0.778	0.494
LD3	0.378	0.763	0.421
**Housework**			
Pre	0.441	0.695	0.460
LD1	0.438	0.709	0.459
LD3	0.413	0.692	0.436
**Leisure**			
Pre	0.341	0.402	0.311
LD1	0.341	0.442	0.328
LD3	0.317	0.449	0.313
**Subsistence**			
Pre	0.208	0.693	0.216
LD1	0.209	0.703	0.217
LD3	0.209	0.730	0.214

Table S13. Gini coefficients of time spent (hours per day) on broad activity categories, by timepoint. Gini coefficients range from 0 (perfect equality) to 1 (complete inequality). 'Pre' refers to pre-pandemic (February 2020), 'LD1' refers to the first national lockdown (May 2020), and 'LD3' refers to the third national lockdown (March 2021). All respondents in our sample were included.

6.4 Correlation between time use and sociodemographic characteristics for broad activity categories

Table S14 shows the regression estimates underlying Figure 2 in our main manuscript.

	Employment		Housework		Leisure		Subsistence	
	LD1	LD3	LD1	LD3	LD1	LD3	LD1	LD3
Female	-0.05	-0.12	-0.18	0.21	-0.06	-0.38*	-0.14	0.06
	(0.25)	(0.21)	(0.16)	(0.17)	(0.20)	(0.21)	(0.15)	(0.14)
Has young child	-0.08	-0.32	1.12***	0.95***	-0.94***	-0.92***	-0.48*	-0.24
	(0.37)	(0.32)	(0.29)	(0.30)	(0.30)	(0.30)	(0.25)	(0.26)
Female x Has young child	-0.93	-0.06	0.38	0.90**	-0.29	-0.21	0.23	-0.09
	(0.61)	(0.48)	(0.38)	(0.41)	(0.41)	(0.40)	(0.34)	(0.33)
WFH during LD1	-0.20		-0.37***		-0.71***		-0.15	
	(0.26)		(0.14)		(0.18)		(0.14)	
WFH during LD3		-0.29		-0.29**		-0.14		-0.00
		(0.18)		(0.14)		(0.17)		(0.13)
Has tertiary degree	-0.27	-0.45**	-0.05	-0.19	-0.09	0.15	0.04	0.04
	(0.23)	(0.21)	(0.15)	(0.16)	(0.19)	(0.20)	(0.14)	(0.15)
White	-0.50*	-0.51*	0.10	0.06	0.61**	0.60**	-0.10	-0.17
	(0.27)	(0.28)	(0.17)	(0.18)	(0.25)	(0.24)	(0.19)	(0.20)
Income: £1k-2k	0.63	1.10***	0.09	-0.05	-0.20	-0.26	-0.10	-0.16
	(0.42)	(0.42)	(0.19)	(0.19)	(0.23)	(0.24)	(0.18)	(0.18)
Income: £2k-3k	0.85*	1.51***	0.07	-0.11	-0.16	-0.34	-0.10	-0.19
	(0.47)	(0.51)	(0.21)	(0.21)	(0.27)	(0.27)	(0.21)	(0.19)
Income: £3k-4k	1.08*	1.45***	-0.23	-0.01	0.07	-0.22	0.12	-0.26
	(0.65)	(0.55)	(0.24)	(0.30)	(0.32)	(0.33)	(0.24)	(0.23)
Income: £4k-5k	0.83	1.77***	-0.16	-0.15	-0.12	-0.34	-0.33	-0.99***
	(0.78)	(0.56)	(0.34)	(0.44)	(0.36)	(0.54)	(0.34)	(0.33)
Income: £5k or above	1.63**	1.90***	-0.28	0.17	0.24	-0.34	-0.77**	-0.59
	(0.72)	(0.61)	(0.28)	(0.37)	(0.50)	(0.45)	(0.33)	(0.39)
Level: Male, no young child	7.61	7.61	2.41	2.41	5.52	5.52	11.23	11.23
Level: Male, young child	8.04	8.04	4.37	4.37	3.93	3.93	10.57	10.57
Level: Female, no young child	6.88	6.88	3.03	3.03	5.01	5.01	11.58	11.58
Level: Female, young child	6.65	6.65	5.19	5.19	3.22	3.22	11.25	11.25
Diff: Male, no young child	-0.58	-0.48	0.64	0.29	0.55	0.94	0.54	-0.04
Diff: Male, young child	-0.42	-0.63	0.97	-0.01	0.21	0.58	0.32	0.17
Diff: Female, no young child	-0.34	-0.26	0.37	0.09	0.71	1.00	0.32	-0.13
Diff: Female, young child	-1.16	-0.40	0.97	0.51	0.31	0.47	0.23	-0.17
Observations	720	709	763	763	763	763	763	763

Table S14. Estimates of time spent (hours per day) on broad activity categories during Lockdown 1 (LD1) and Lockdown 3 (LD3), by sociodemographic characteristics. Time spent was calculated using main activities only. Regressions with employment as the dependent variable used Heckman corrections to account for selection effects with bootstrapped standard errors (1000 replications). In addition to the variables reported, we also controlled for age, and pre-pandemic levels in total time spent on the given activity. Robust standard errors are reported in brackets. *** $p < 0.01$, ** $p < 0.05$, * $p < 0.1$.

Table S15 shows the association between time spent on travelling and studying during the two lockdowns and individual characteristics.

	Travel		Study	
	LD1	LD3	LD1	LD3
Female	0.00	-0.03	0.06	0.05
	(0.02)	(0.05)	(0.09)	(0.10)
Has young child	0.04	0.00	-0.05	-0.14
	(0.03)	(0.07)	(0.08)	(0.09)
Female x Has young child	-0.03	-0.02	0.04	0.07
	(0.05)	(0.09)	(0.18)	(0.16)
WFH during LD1	-0.09***		-0.20***	
	(0.02)		(0.07)	
WFH during LD3		-0.31***		-0.20***
		(0.04)		(0.07)
Has tertiary degree	-0.02	-0.01	0.25***	0.02
	(0.02)	(0.04)	(0.08)	(0.09)
White	-0.03	-0.02	-0.07	-0.06
	(0.03)	(0.05)	(0.15)	(0.13)
Income: £1k-2k	0.05**	0.16**	-0.21*	-0.29**
	(0.02)	(0.06)	(0.11)	(0.12)
Income: £2k-3k	0.04	0.09	-0.32**	-0.36***
	(0.02)	(0.05)	(0.12)	(0.11)
Income: £3k-4k	0.04	0.13*	-0.45***	-0.30**
	(0.03)	(0.07)	(0.11)	(0.12)
Income: £4k-5k	0.11	0.21**	-0.24*	-0.07
	(0.08)	(0.10)	(0.13)	(0.30)
Income: £5k or above	-0.01	0.13	-0.16	-0.22
	(0.03)	(0.12)	(0.20)	(0.15)
Level: Male, no young child	0.68	0.68	0.33	0.33
Level: Male, young child	0.87	0.87	0.09	0.09
Level: Female, no young child	0.64	0.64	0.48	0.48
Level: Female, young child	0.73	0.73	0.32	0.32
Diff: Male, no young child	-0.61	-0.42	0.05	-0.00
Diff: Male, young child	-0.75	-0.57	0.03	0.05
Diff: Female, no young child	-0.57	-0.43	0.13	-0.01
Diff: Female, young child	-0.63	-0.52	0.19	0.10
Observations	763	763	763	763

Table S15. Estimates of time spent (hours per day) on travel and studying during Lockdown 1 (LD1) and Lockdown 3 (LD3), by sociodemographic characteristics. Time spent was calculated using main activities only. In addition to the variables reported, we also controlled for age, and pre-pandemic levels in total time spent on the given activity. Robust standard errors are reported in brackets. *** $p < 0.01$, ** $p < 0.05$, * $p < 0.1$.

Table S16 shows the regression estimates from the Heckman selection equation corresponding to columns (1) and (2) of Table S14.

	Working during this period	
	**Lockdown 1**	**Lockdown 3**
Working during pre-pandemic period	7.73***	7.86***
	(1.85)	(2.03)
% Time working from home pre-pandemic	0.01***	0.00**
	(0.00)	(0.00)
Female	0.35***	0.04
	(0.13)	(0.15)
Has young child	0.46	1.11
	(0.34)	(2.35)
Female X Has young child	-0.87**	-1.20
	(0.41)	(2.38)
Tertiary degree	0.09	0.21
	(0.13)	(0.14)
White	-0.09	-0.33
	(0.20)	(0.24)
Income: £1k-2k	0.72***	0.60***
	(0.15)	(0.16)
Income: £2k-3k	0.77***	0.92***
	(0.18)	(0.22)
Income: £3k-4k	1.44***	1.02*
	(0.27)	(0.55)
Income: £4k-5k	1.64	0.82
	(1.67)	(5.06)
Income: £5k or above	1.57	1.34
	(17.79)	(11.06)
Observations	720	709

Table S16. Estimates of selection equation using Heckman's two step estimator. 'Working during pre-pandemic period' is a binary indicator that equals 1 if the respondent was working in the pre-pandemic period and zero otherwise. '% time working from home pre-pandemic' is a continuous variable, ranging from 0 to 100, that measures self-reported percentage of time in a typical work week (pre-pandemic) that the respondent worked from home. Time spent was calculated using the main activity only. Robust standard errors are reported in brackets. *** $p < 0.01$, ** $p < 0.05$, * $p < 0.1$.

Table S17 shows the regression estimates from regressing time spent on employment during Lockdown 1 and Lockdown 3, without correcting for selection into employment.

	Employment (not Heckman-corrected)	
	**Lockdown 1**	**Lockdown 3**
Female	0.07	-0.06
	(0.21)	(0.19)
Has young child	-0.04	-0.32
	(0.34)	(0.26)
Female X Has young child	-1.15**	-0.14
	(0.49)	(0.42)
WFH during LD1	-0.27	
	(0.24)	
WFH during LD3		-0.27
		(0.17)
Tertiary degree	-0.21	-0.42**
	(0.21)	(0.19)
White	-0.42	-0.51**
	(0.26)	(0.25)
Income: £1k-2k	0.85***	1.06***
	(0.32)	(0.30)
Income: £2k-3k	1.05***	1.41***
	(0.33)	(0.30)
Income: £3k-4k	1.34***	1.37***
	(0.37)	(0.34)
Income: £4k-5k	1.16**	1.73***
	(0.46)	(0.37)
Income: £5k or above	1.98***	1.81***
	(0.47)	(0.40)
Level: Male, no young child	7.61	7.61
Level: Male, young child	8.04	8.04
Level: Female, no young child	6.88	6.88
Level: Female, young child	6.65	6.65
Diff: Male, no young child	-0.58	-0.48
Diff: Male, young child	-0.42	-0.63
Diff: Female, no young child	-0.34	-0.26
Diff: Female, young child	-1.16	-0.40
Observations	471	534

Table S17. Estimates of time spent (hours per day) on employment activity domains by sociodemographic characteristics, without correcting for selection into employment. Regressions used the subset of individuals who remained employed in both periods of interest. Regressions also controlled for age and pre-pandemic levels in total time spent on employment activities. Time spent was calculated using the main activity only. Robust standard errors are reported in brackets. *** $p < 0.01$, ** $p < 0.05$, * $p < 0.1$.

6.5 Correlation between time use and sociodemographic characteristics for specific activity categories

We re-ran the regression specification using our main measure of time use (main activity only), where the outcome variable was change in time spent on activity subcategories. Table S18 shows the results for employment activity subcategories (estimated using the Heckman selection model). Table S19 shows the results for housework activity subcategories. Table S20 shows the results for leisure activity subcategories. Lastly, Table S21 shows the results for subsistence activity subcategories.

	**Work tasks**		**Meetings**		**Job search**		**Other employment activities**	
	LD1	LD3	LD1	LD3	LD1	LD3	LD1	LD3
Female	-0.03	-0.05	-0.07	0.12	0.01	-0.01	-0.01	-0.16*
	(0.28)	(0.23)	(0.14)	(0.13)	(0.01)	(0.01)	(0.07)	(0.09)
Has young child	0.18	0.03	-0.15	-0.19	-0.00	-0.02	-0.12*	-0.14
	(0.44)	(0.40)	(0.20)	(0.22)	(0.00)	(0.02)	(0.06)	(0.16)
Female X Has young child	-1.14*	-0.13	0.19	-0.25	-0.00	-0.01	0.08	0.30
	(0.67)	(0.54)	(0.31)	(0.30)	(0.01)	(0.03)	(0.12)	(0.21)
WFH during LD1	-0.34		0.13		-0.01		-0.03	
	(0.27)		(0.11)		(0.01)		(0.06)	
WFH during LD3		-0.51**		0.20*		-0.00		0.02
		(0.20)		(0.11)		(0.01)		(0.07)
Tertiary degree	-0.14	-0.42*	-0.20*	0.06	-0.01	0.01	0.06	-0.10
	(0.26)	(0.24)	(0.12)	(0.13)	(0.01)	(0.01)	(0.05)	(0.09)
White	-0.01	-0.22	-0.37**	-0.28*	0.00	-0.01	-0.07	-0.02
	(0.34)	(0.29)	(0.17)	(0.16)	(0.00)	(0.01)	(0.09)	(0.12)
Income: £1k-2k	0.64	1.02**	0.01	0.31	0.00	0.01	-0.08	-0.27
	(0.42)	(0.44)	(0.16)	(0.24)	(0.00)	(0.02)	(0.11)	(0.17)
Income: £2k-3k	0.48	1.40**	0.32*	0.56*	-0.00	-0.01	-0.05	-0.47**
	(0.47)	(0.57)	(0.19)	(0.31)	(0.00)	(0.03)	(0.11)	(0.22)
Income: £3k-4k	0.99	1.27**	-0.02	0.82**	-0.00	-0.01	-0.06	-0.64***
	(0.68)	(0.62)	(0.31)	(0.34)	(0.01)	(0.03)	(0.16)	(0.24)
Income: £4k-5k	0.19	1.02	0.55	1.20***	0.00	-0.02	-0.04	-0.41
	(0.77)	(0.66)	(0.41)	(0.36)	(0.01)	(0.03)	(0.19)	(0.25)
Income: £5k or above	0.40	0.62	1.14**	1.98***	0.00	-0.01	-0.13	-0.71**
	(0.83)	(0.76)	(0.48)	(0.42)	(0.01)	(0.04)	(0.17)	(0.29)
Level: Male, no young child	7.04	7.04	0.30	0.30	0.00	0.00	0.27	0.27
Level: Male, young child	7.30	7.30	0.51	0.51	0.00	0.00	0.23	0.23
Level: Female, no young child	6.18	6.18	0.39	0.39	0.02	0.02	0.30	0.30
Level: Female, young child	6.26	6.26	0.17	0.17	0.00	0.00	0.22	0.22
Diff: Male, no young child	-0.67	-0.77	0.11	0.25	0.00	0.02	-0.01	0.03
Diff: Male, young child	-0.11	-0.67	-0.07	-0.01	0.00	0.00	-0.23	0.04
Diff: Female, no young child	-0.15	-0.28	-0.17	0.11	0.01	-0.01	-0.03	-0.07
Diff: Female, young child	-1.16	-0.60	0.03	0.16	0.00	0.00	-0.04	0.04
Observations	720	706	720	706	720	706	720	706

Table S18. Estimates of time spent (hours per day) on employment activity subcategories, by sociodemographic characteristics. Estimates corrected for selection into employment using Heckman's two step estimator with bootstrapped standard errors (1000 replications). 'Other' employment activities included causal work, breaks, and any other employment activity. Time spent was calculated using the main activity only. Regressions also controlled for age and pre-pandemic levels in total time spent on the given activity. Robust standard errors are reported in brackets. *** $p < 0.01$, ** $p < 0.05$, * $p < 0.1$.

	Caring/Childcare		Cooking/Groceries		Cleaning		Other housework	
	LD1	LD3	LD1	LD3	LD1	LD3	LD1	LD3
Female	-0.02	0.02	-0.06	0.17**	0.15**	0.24***	-0.10	-0.17
	(0.06)	(0.05)	(0.10)	(0.08)	(0.06)	(0.07)	(0.12)	(0.11)
Has young child	0.94***	1.27***	-0.09	-0.06	0.01	-0.01	0.24*	-0.22*
	(0.27)	(0.26)	(0.13)	(0.13)	(0.10)	(0.11)	(0.14)	(0.12)
Female X Has young child	0.17	0.44	0.56***	0.32*	0.08	-0.20	-0.36	0.41**
	(0.33)	(0.34)	(0.19)	(0.19)	(0.18)	(0.16)	(0.23)	(0.17)
WFH during LD1	-0.11		-0.02		-0.11*		-0.23**	
	(0.08)		(0.08)		(0.06)		(0.10)	
WFH during LD3		-0.11		0.10		-0.12**		-0.18**
		(0.07)		(0.07)		(0.06)		(0.08)
Tertiary degree	-0.18**	-0.18**	-0.04	0.02	0.02	-0.13**	0.11	0.07
	(0.09)	(0.08)	(0.09)	(0.08)	(0.07)	(0.07)	(0.11)	(0.10)
White	0.02	0.04	-0.03	-0.04	-0.02	-0.05	0.10	0.09
	(0.10)	(0.13)	(0.11)	(0.11)	(0.07)	(0.09)	(0.11)	(0.08)
Income: £1k-2k	0.04	-0.12	-0.15	-0.05	-0.04	-0.05	0.19	0.16
	(0.09)	(0.09)	(0.11)	(0.09)	(0.09)	(0.08)	(0.15)	(0.12)
Income: £2k-3k	0.09	-0.18*	0.07	0.06	-0.12	-0.04	-0.00	0.03
	(0.11)	(0.11)	(0.13)	(0.11)	(0.09)	(0.09)	(0.14)	(0.11)
Income: £3k-4k	0.19	0.01	-0.15	0.04	-0.11	-0.21*	-0.28*	0.09
	(0.14)	(0.17)	(0.13)	(0.13)	(0.11)	(0.11)	(0.16)	(0.18)
Income: £4k-5k	0.26	0.03	-0.45***	-0.09	-0.23*	-0.18	0.29	0.10
	(0.19)	(0.22)	(0.15)	(0.18)	(0.13)	(0.12)	(0.27)	(0.20)
Income: £5k or above	0.21	0.09	-0.00	-0.06	-0.22*	-0.28**	-0.36**	0.37*
	(0.18)	(0.22)	(0.17)	(0.18)	(0.12)	(0.11)	(0.18)	(0.22)
Level: Male, no young child	0.14	0.14	1.17	1.17	0.33	0.33	0.77	0.77
Level: Male, young child	2.36	2.36	1.15	1.15	0.38	0.38	0.49	0.49
Level: Female, no young child	0.18	0.18	1.41	1.41	0.76	0.76	0.68	0.68
Level: Female, young child	2.01	2.01	1.49	1.49	1.01	1.01	0.68	0.68
Diff: Male, no young child	0.08	0.00	0.06	0.02	0.08	0.19	0.41	0.07
Diff: Male, young child	0.55	-0.11	-0.09	-0.03	0.07	0.16	0.44	-0.03
Diff: Female, no young child	0.01	-0.02	-0.08	0.01	0.03	0.16	0.40	-0.06
Diff: Female, young child	0.74	0.56	0.24	0.16	-0.03	-0.23	0.02	0.02
Observations	763	763	763	763	763	763	763	763

Table S19. Estimates of time spent (hours per day) on housework activity subcategories, by sociodemographic characteristics. Time spent was calculated using the main activity only. ‘Other’ housework includes caring for pets, bills, gardening, doing repairs, and any other uncategorized housework activity. Regressions also controlled for age and pre-pandemic levels in total time spent on the given activity. Robust standard errors are reported in brackets. *** $p < 0.01$, ** $p < 0.05$, * $p < 0.1$.

	Social		Exercise		Hobbies		Media		Volunteer	
	**LD1**	**LD3**	**LD1**	**LD3**	**LD1**	**LD3**	**LD1**	**LD3**	**LD1**	**LD3**
Female	0.17**	-0.09	0.00	0.02	-0.28***	-0.29**	0.13	0.12	0.11***	0.01
	(0.08)	(0.07)	(0.07)	(0.07)	(0.11)	(0.11)	(0.17)	(0.19)	(0.04)	(0.03)
Has young child	-0.01	-0.10	-0.12	-0.09	-0.51***	-0.11	-0.27	-0.57**	0.09	0.06
	(0.10)	(0.10)	(0.10)	(0.12)	(0.14)	(0.16)	(0.26)	(0.25)	(0.06)	(0.04)
Female X Has young child	-0.16	-0.10	-0.10	-0.11	0.34*	0.14	-0.34	-0.15	-0.17**	-0.07
	(0.14)	(0.13)	(0.14)	(0.15)	(0.19)	(0.18)	(0.36)	(0.35)	(0.07)	(0.05)
WFH during LD1	0.11*		-0.05		-0.05		-0.42***		0.01	
	(0.06)		(0.06)		(0.09)		(0.16)		(0.04)	
WFH during LD3		0.02		0.07		-0.16*		-0.05		-0.04**
		(0.06)		(0.07)		(0.08)		(0.15)		(0.02)
Tertiary degree	0.05	0.13*	0.04	0.11	-0.12	-0.12	0.09	0.16	0.02	0.02
	(0.06)	(0.07)	(0.06)	(0.07)	(0.10)	(0.10)	(0.17)	(0.18)	(0.03)	(0.03)
White	0.03	-0.06	0.07	0.16*	0.13	0.34***	0.58***	0.30	-0.12*	-0.12**
	(0.07)	(0.08)	(0.08)	(0.09)	(0.13)	(0.09)	(0.19)	(0.24)	(0.06)	(0.06)
Income: £1k-2k	0.00	-0.10	0.01	-0.01	-0.11	0.02	-0.11	-0.06	0.08	-0.03
	(0.08)	(0.08)	(0.08)	(0.08)	(0.13)	(0.13)	(0.20)	(0.22)	(0.05)	(0.03)
Income: £2k-3k	-0.04	-0.04	-0.13	0.00	0.01	0.02	0.02	-0.15	0.08*	-0.03
	(0.09)	(0.10)	(0.09)	(0.09)	(0.15)	(0.14)	(0.24)	(0.24)	(0.04)	(0.04)
Income: £3k-4k	-0.02	-0.15	0.05	0.07	-0.05	-0.07	0.09	0.13	0.04	-0.06**
	(0.11)	(0.12)	(0.12)	(0.13)	(0.17)	(0.16)	(0.29)	(0.28)	(0.04)	(0.03)
Income: £4k-5k	-0.21**	0.05	-0.03	0.35	-0.31*	-0.31**	0.11	-0.37	0.20	-0.01
	(0.10)	(0.22)	(0.15)	(0.31)	(0.16)	(0.16)	(0.32)	(0.42)	(0.15)	(0.07)
Income: £5k or above	-0.04	-0.39***	0.07	0.11	-0.47**	0.07	0.35	-0.19	0.04	-0.02
	(0.22)	(0.11)	(0.18)	(0.17)	(0.21)	(0.25)	(0.41)	(0.38)	(0.06)	(0.07)
Level: Male, no young child	1.03	1.03	0.68	0.68	0.61	0.61	3.14	3.14	0.05	0.05
Level: Male, young child	0.92	0.92	0.62	0.62	0.49	0.49	1.90	1.90	0.01	0.01
Level: Female, no young child	1.26	1.26	0.63	0.63	0.44	0.44	2.59	2.59	0.09	0.09
Level: Female, young child	0.83	0.83	0.48	0.48	0.18	0.18	1.69	1.69	0.04	0.04
Diff: Male, no young child	-0.74	-0.52	0.08	0.14	0.44	0.37	0.80	0.95	-0.03	-0.00
Diff: Male, young child	-0.65	-0.58	-0.02	0.10	0.01	0.18	0.82	0.83	0.06	0.04
Diff: Female, no young child	-0.77	-0.75	0.11	0.17	0.21	0.17	1.10	1.42	0.06	-0.01
Diff: Female, young child	-0.59	-0.67	-0.02	0.08	0.16	0.12	0.77	0.96	-0.01	-0.02
Observations	763	763	763	763	763	763	763	763	763	763

Table S20. Estimates of time spent (hours per day) on leisure activity subcategories, by sociodemographic characteristics. Time spent was calculated using the main activity only. Regressions also controlled for age and pre-pandemic levels in total time spent on the given activity. Robust standard errors are reported in brackets. *** $p < 0.01$, ** $p < 0.05$, * $p < 0.1$.

	Sleeping		Eating		Grooming	
	**LD1**	**LD3**	**LD1**	**LD3**	**LD1**	**LD3**
Female	-0.06	-0.03	-0.07	-0.06	0.00	0.17***
	(0.13)	(0.12)	(0.08)	(0.09)	(0.07)	(0.06)
Has young child	-0.28	0.10	0.10	-0.23	-0.30***	-0.16*
	(0.22)	(0.27)	(0.13)	(0.14)	(0.09)	(0.09)
Female X Has young child	-0.04	-0.13	-0.05	0.00	0.31**	0.06
	(0.32)	(0.32)	(0.19)	(0.18)	(0.15)	(0.14)
WFH during LD1	-0.07		-0.04		-0.08	
	(0.12)		(0.08)		(0.07)	
WFH during LD3		-0.01		0.02		-0.01
		(0.11)		(0.09)		(0.06)
Tertiary degree	-0.09	-0.28**	0.15*	0.25***	-0.02	0.07
	(0.13)	(0.13)	(0.08)	(0.09)	(0.07)	(0.07)
White	-0.00	0.13	-0.14	-0.14	0.04	-0.19*
	(0.18)	(0.17)	(0.12)	(0.14)	(0.10)	(0.10)
Income: £1k-2k	0.00	-0.01	-0.04	-0.16	-0.07	-0.01
	(0.16)	(0.15)	(0.10)	(0.11)	(0.09)	(0.09)
Income: £2k-3k	0.13	0.06	-0.11	-0.21	-0.12	-0.04
	(0.18)	(0.16)	(0.12)	(0.13)	(0.09)	(0.09)
Income: £3k-4k	0.22	0.06	-0.04	-0.21	-0.06	-0.13
	(0.19)	(0.17)	(0.15)	(0.16)	(0.14)	(0.11)
Income: £4k-5k	-0.06	-0.54	-0.03	-0.19	-0.24**	-0.27*
	(0.27)	(0.35)	(0.17)	(0.20)	(0.09)	(0.14)
Income: £5k or above	-0.46	-0.39	-0.22	-0.07	-0.10	-0.13
	(0.29)	(0.30)	(0.16)	(0.31)	(0.11)	(0.16)
Level: Male, no young child	8.93	8.93	1.51	1.51	0.79	0.79
Level: Male, young child	8.89	8.89	1.14	1.14	0.54	0.54
Level: Female, no young child	9.20	9.20	1.44	1.44	0.94	0.94
Level: Female, young child	9.54	9.54	1.12	1.12	0.60	0.60
Diff: Male, no young child	0.52	-0.22	0.02	0.15	-0.01	0.03
Diff: Male, young child	0.31	-0.13	0.19	0.18	-0.18	0.12
Diff: Female, no young child	0.36	-0.44	0.02	0.20	-0.06	0.11
Diff: Female, young child	-0.04	-0.78	0.11	0.25	0.16	0.36
Observations	763	763	763	763	763	763

Table S21. Estimates of time spent (hours per day) on subsistence activity subcategories, by sociodemographic characteristics. Time spent was calculated using the main activity only. Regressions also controlled for age and pre-pandemic levels in total time spent on the given activity. Robust standard errors are reported in brackets. *** $p < 0.01$, ** $p < 0.05$, * $p < 0.1$.

6.6 Changes in enjoyment

Table S22 shows the regression estimates underlying Figure 5 in our main manuscript.

	Change in overall enjoyment	
	**LD1 vs Pre**	**LD3 vs Pre**
**A. Quantity: Changes (hours per day) spent on:**		
Housework	0.01	0.04*
	(0.02)	(0.02)
Employment	-0.00	0.02
	(0.01)	(0.02)
Leisure	0.07***	0.07***
	(0.02)	(0.02)
Subsistence	0.04**	0.03
	(0.02)	(0.02)
**B. Quality: Changes (hours per day) spent on:**		
Multitasking across categories	0.01*	0.01
	(0.01)	(0.01)
Leisure activity done alone	-0.03**	-0.04**
	(0.01)	(0.02)
Unusual work hours	-0.02	-0.08***
	(0.02)	(0.03)
Unusual housework hours	-0.01	-0.04
	(0.03)	(0.05)
**C. Sociodemographic covariates**		
WFH during LD1	0.14**	
	(0.06)	
WFH during LD3		0.06
		(0.07)
Female	-0.13**	0.07
	(0.05)	(0.08)
Has young child	0.01	0.04
	(0.08)	(0.12)
Female X Has young child	0.09	-0.19
	(0.11)	(0.14)
Has tertiary degree	-0.03	0.14**
	(0.05)	(0.07)
White	-0.10	-0.11
	(0.07)	(0.10)
Income: £1k-2k	-0.06	-0.17*
	(0.06)	(0.09)
Income: £2k-3k	-0.15**	-0.22**
	(0.07)	(0.11)
Income: £3k-4k	0.01	-0.03
	(0.07)	(0.13)
Income: £4k-5k	-0.00	0.04
	(0.10)	(0.16)
Income: £5k or above	-0.11	0.22
	(0.16)	(0.16)
Baseline mean enjoyment	5.64	5.64
Baseline SD enjoyment	0.76	0.76
Baseline mean change in enjoyment	-0.01	-0.28
Baseline SD change in enjoyment	0.61	0.94
Observations	762	761

Table S22. Estimates of correlations between within-person changes in overall self-reported enjoyment and characteristics of time use. Sociodemographic covariates were used as controls. Reported changes during the first and third lockdown (May 2020 and March 2021, respectively) were relative to the pre-pandemic timepoint (February 2020). Robust standard errors are reported in brackets. A coefficient of 0.1 corresponds to ~0.13 SD in pre-pandemic enjoyment levels. *** $p < 0.01$, ** $p < 0.05$, * $p < 0.1$.

7 Robustness to attrition

In Section 2.2, we found evidence that older respondents were more likely to complete both survey waves. To account for potential bias arising from this differential attrition, we used inverse probability weights to re-weight our longitudinal sample. Specifically, we used the probit estimates reported in Table S3 to obtain predicted probabilities of each respondent appearing in both survey waves, conditional on sociodemographic characteristics. We then used the inverse of the predicted probability as that respondent's weight.

Tables S23-S34 compare the weighted results with the unweighted results presented in Figures 1-4 of our main manuscript. Our results were qualitatively similar.

7.1 Quantity measures: Inverse probability weights

		**Unweighted**		**Weighted**	
**Subgroup**	**Timepoint**	Mean	95% CI	Mean	95% CI
**Levels (Hours)**					
Men	Pre	7.70	[7.46, 7.94]	7.77	[7.46, 8.07]
Men	LD 1	7.26	[6.98, 7.55]	7.34	[7, 7.68]
Men	LD 3	7.41	[7.18, 7.65]	7.45	[7.14, 7.76]
Women	Pre	6.84	[6.58, 7.1]	6.84	[6.55, 7.12]
Women	LD 1	6.55	[6.25, 6.85]	6.55	[6.21, 6.88]
Women	LD 3	6.83	[6.56, 7.11]	6.98	[6.67, 7.29]
No young child	Pre	7.24	[7.04, 7.44]	7.36	[7.1, 7.62]
No young child	LD 1	6.91	[6.68, 7.15]	7.03	[6.75, 7.31]
No young child	LD 3	7.17	[6.97, 7.37]	7.33	[7.08, 7.59]
Has young child	Pre	7.41	[7.02, 7.8]	7.55	[7.16, 7.93]
Has young child	LD 1	6.92	[6.45, 7.38]	7.04	[6.5, 7.58]
Has young child	LD 3	6.98	[6.59, 7.38]	7.06	[6.6, 7.53]
**Differences (Hours)**					
Men	Pre vs LD 1	-0.54	[-0.82, -0.26]	-0.53	[-0.84, -0.22]
Men	Pre vs LD 3	-0.51	[-0.81, -0.22]	-0.55	[-0.95, -0.15]
Women	Pre vs LD 1	-0.48	[-0.78, -0.19]	-0.42	[-0.74, -0.1]
Women	Pre vs LD 3	-0.28	[-0.6, 0.03]	-0.05	[-0.39, 0.3]
No young child	Pre vs LD 1	-0.46	[-0.67, -0.24]	-0.47	[-0.7, -0.24]
No young child	Pre vs LD 3	-0.37	[-0.61, -0.13]	-0.31	[-0.66, 0.04]
Has young child	Pre vs LD 1	-0.72	[-1.23, -0.21]	-0.53	[-1.14, 0.07]
Has young child	Pre vs LD 3	-0.53	[-0.97, -0.1]	-0.51	[-0.92, -0.11]

Table S23. Weighted and unweighted mean time spent and within-person differences in time spent (hours per day) on employment-related activities, by timepoint. Inverse probability weights were used to construct weighted averages. 'Pre-pandemic' refers to February 2020, 'LD 1' refers to the first lockdown (May 2020), 'LD 3' refers to the third lockdown (March 2021). Within-person changes for employment activities are calculated using the subset of individuals who remained employed in both periods of interest. 95% confidence intervals are reported in brackets. Note that the conditional means were calculated separately (either by gender or household composition), so the four subgroups shown are not mutually exclusive.

Subgroup	Timepoint	Unweighted		Weighted	
		Mean	95% CI	Mean	95% CI
**Levels (Hours)**					
Men	Pre	2.77	[2.55, 2.99]	2.47	[2.2, 2.75]
Men	LD 1	3.47	[3.18, 3.75]	3.03	[2.68, 3.39]
Men	LD 3	3.00	[2.77, 3.23]	2.79	[2.5, 3.07]
Women	Pre	3.38	[3.14, 3.61]	2.95	[2.69, 3.21]
Women	LD 1	3.84	[3.59, 4.1]	3.38	[3.09, 3.68]
Women	LD 3	3.54	[3.31, 3.76]	3.20	[2.92, 3.48]
No young child	Pre	2.73	[2.57, 2.9]	2.19	[2.02, 2.36]
No young child	LD 1	3.23	[3.03, 3.43]	2.59	[2.38, 2.79]
No young child	LD 3	2.92	[2.75, 3.08]	2.48	[2.29, 2.67]
Has young child	Pre	4.77	[4.36, 5.18]	4.59	[4.1, 5.07]
Has young child	LD 1	5.74	[5.32, 6.16]	5.53	[4.91, 6.15]
Has young child	LD 3	5.01	[4.61, 5.42]	4.88	[4.41, 5.34]
**Differences (Hours)**					
Men	Pre vs LD 1	0.70	[0.49, 0.91]	0.56	[0.34, 0.78]
Men	Pre vs LD 3	0.23	[0, 0.47]	0.31	[0.04, 0.58]
Women	Pre vs LD 1	0.47	[0.29, 0.64]	0.44	[0.25, 0.62]
Women	Pre vs LD 3	0.16	[-0.07, 0.39]	0.25	[0, 0.51]
No young child	Pre vs LD 1	0.50	[0.35, 0.64]	0.40	[0.25, 0.54]
No young child	Pre vs LD 3	0.18	[0.01, 0.36]	0.29	[0.09, 0.48]
Has young child	Pre vs LD 1	0.97	[0.61, 1.33]	0.95	[0.5, 1.4]
Has young child	Pre vs LD 3	0.24	[-0.23, 0.71]	0.29	[-0.22, 0.8]

Table S24. Weighted and unweighted mean time spent and within-person differences in time spent (hours per day) on housework-related activities, by timepoint. Inverse probability weights were used to construct weighted averages. 'Pre-pandemic' refers to February 2020, 'LD 1' refers to the first lockdown (May 2020), 'LD 3' refers to the third lockdown (March 2021). 95% confidence intervals are reported in brackets. Note that the conditional means were calculated separately (either by gender or household composition), so the four subgroups shown are not mutually exclusive.

Subgroup	Timepoint	Unweighted		Weighted	
		Mean	95% CI	Mean	95% CI
**Levels (Hours)**					
Men	Pre	5.22	[4.97, 5.48]	5.07	[4.74, 5.4]
Men	LD 1	5.71	[5.42, 6]	5.68	[5.3, 6.06]
Men	LD 3	6.10	[5.81, 6.38]	5.73	[5.37, 6.09]
Women	Pre	4.72	[4.48, 4.96]	4.74	[4.44, 5.04]
Women	LD 1	5.36	[5.09, 5.63]	5.24	[4.94, 5.54]
Women	LD 3	5.63	[5.37, 5.9]	5.53	[5.24, 5.83]
No young child	Pre	5.25	[5.05, 5.44]	5.29	[5.04, 5.53]
No young child	LD 1	5.88	[5.67, 6.09]	5.86	[5.6, 6.13]
No young child	LD 3	6.22	[6.01, 6.44]	6.05	[5.78, 6.33]
Has young child	Pre	3.59	[3.25, 3.92]	3.51	[3.03, 3.99]
Has young child	LD 1	3.85	[3.45, 4.24]	4.04	[3.39, 4.69]
Has young child	LD 3	4.11	[3.77, 4.45]	4.04	[3.65, 4.43]
**Differences (Hours)**					
Men	Pre vs LD 1	0.49	[0.23, 0.75]	0.61	[0.23, 1]
Men	Pre vs LD 3	0.87	[0.59, 1.16]	0.66	[0.26, 1.06]
Women	Pre vs LD 1	0.64	[0.4, 0.89]	0.50	[0.21, 0.79]
Women	Pre vs LD 3	0.92	[0.62, 1.21]	0.80	[0.44, 1.15]
No young child	Pre vs LD 1	0.63	[0.43, 0.83]	0.58	[0.3, 0.85]
No young child	Pre vs LD 3	0.97	[0.74, 1.21]	0.76	[0.45, 1.08]
Has young child	Pre vs LD 1	0.26	[-0.13, 0.65]	0.53	[-0.09, 1.16]
Has young child	Pre vs LD 3	0.52	[0.1, 0.94]	0.53	[0, 1.07]

Table S25. Weighted and unweighted mean time spent and within-person differences in time spent (hours per day) on leisure activities, by timepoint. Inverse probability weights were used to construct weighted averages. ‘Pre-pandemic’ refers to February 2020, ‘LD 1’ refers to the first lockdown (May 2020), ‘LD 3’ refers to the third lockdown (March 2021). 95% confidence intervals are reported in brackets. Note that the conditional means were calculated separately (either by gender or household composition), so the four subgroups shown are not mutually exclusive.

		Unweighted		Weighted	
**Subgroup**	**Timepoint**	Mean	95% CI	Mean	95% CI
**Levels (Hours)**					
Men	Pre	11.11	[10.92, 11.3]	11.37	[11.11, 11.62]
Men	LD 1	11.60	[11.38, 11.83]	11.83	[11.47, 12.19]
Men	LD 3	11.11	[10.92, 11.29]	11.43	[11.13, 11.73]
Women	Pre	11.52	[11.31, 11.73]	11.67	[11.44, 11.9]
Women	LD 1	11.83	[11.61, 12.05]	12.12	[11.88, 12.36]
Women	LD 3	11.39	[11.19, 11.59]	11.57	[11.35, 11.79]
No young child	Pre	11.41	[11.26, 11.57]	11.59	[11.4, 11.79]
No young child	LD 1	11.83	[11.66, 12.01]	12.10	[11.87, 12.33]
No young child	LD 3	11.32	[11.17, 11.48]	11.62	[11.4, 11.84]
Has young child	Pre	10.90	[10.55, 11.25]	11.12	[10.68, 11.55]
Has young child	LD 1	11.18	[10.8, 11.55]	11.37	[10.69, 12.05]
Has young child	LD 3	10.91	[10.6, 11.21]	10.98	[10.57, 11.38]
**Differences (Hours)**					
Men	Pre vs LD 1	0.50	[0.31, 0.69]	0.46	[0.19, 0.74]
Men	Pre vs LD 3	0.00	[-0.22, 0.22]	0.06	[-0.25, 0.38]
Women	Pre vs LD 1	0.31	[0.1, 0.51]	0.45	[0.22, 0.68]
Women	Pre vs LD 3	-0.14	[-0.38, 0.11]	-0.10	[-0.36, 0.16]
No young child	Pre vs LD 1	0.42	[0.27, 0.58]	0.51	[0.31, 0.71]
No young child	Pre vs LD 3	-0.09	[-0.27, 0.09]	0.03	[-0.2, 0.25]
Has young child	Pre vs LD 1	0.28	[-0.05, 0.61]	0.25	[-0.22, 0.73]
Has young child	Pre vs LD 3	0.01	[-0.38, 0.39]	-0.14	[-0.68, 0.41]

Table S26. Weighted and unweighted mean time spent and within-person differences in time spent (hours per day) on subsistence activities, by timepoint. Inverse probability weights were used to construct weighted averages. ‘Pre-pandemic’ refers to February 2020, ‘LD 1’ refers to the first lockdown (May 2020), ‘LD 3’ refers to the third lockdown (March 2021). 95% confidence intervals are reported in brackets. Note that the conditional means were calculated separately (either by gender or household composition), so the four subgroups shown are not mutually exclusive.

7.2 Regression tables: Inverse probability weights

	Employment		Employment		Housework		Housework	
	Lockdown 1		Lockdown 3		Lockdown 1		Lockdown 3	
	Unweighted	Weighted	Unweighted	Weighted	Unweighted	Weighted	Unweighted	Weighted
Female	0.07	-0.02	-0.06	0.07	-0.18	-0.15	0.21	0.08
	(0.21)	(0.24)	(0.19)	(0.25)	(0.16)	(0.15)	(0.17)	(0.17)
Has young child	-0.04	-0.05	-0.32	-0.05	1.12***	1.01***	0.95***	0.95***
	(0.34)	(0.42)	(0.26)	(0.33)	(0.29)	(0.36)	(0.30)	(0.35)
Female x Has young child	-1.15**	-0.85	-0.14	-0.26	0.38	0.58	0.90**	1.22***
	(0.49)	(0.52)	(0.42)	(0.51)	(0.38)	(0.45)	(0.41)	(0.45)
WFH during LD1	-0.27	-0.16			-0.37***	-0.28*		
	(0.24)	(0.31)			(0.14)	(0.16)		
WFH during LD3			-0.27	-0.26			-0.29**	-0.22
			(0.17)	(0.23)			(0.14)	(0.16)
Has tertiary degree	-0.21	-0.11	-0.42**	-0.29	-0.05	-0.02	-0.19	-0.26
	(0.21)	(0.24)	(0.19)	(0.27)	(0.15)	(0.16)	(0.16)	(0.20)
White								
Income: £1k-2k	0.85***	0.73**	1.06***	0.76*	0.09	0.07	-0.05	0.17
	(0.32)	(0.35)	(0.30)	(0.40)	(0.19)	(0.19)	(0.19)	(0.20)
Income: £2k-3k	1.05***	1.01***	1.41***	1.36***	0.07	0.02	-0.11	-0.10
	(0.33)	(0.38)	(0.30)	(0.41)	(0.21)	(0.23)	(0.21)	(0.21)
Income: £3k-4k	1.34***	0.55	1.37***	0.97**	-0.23	0.09	-0.01	0.46*
	(0.37)	(0.47)	(0.34)	(0.41)	(0.24)	(0.26)	(0.30)	(0.28)
Income: £4k-5k	1.16**	0.91	1.73***	1.55***	-0.16	-0.13	-0.15	-0.12
	(0.46)	(0.59)	(0.37)	(0.45)	(0.34)	(0.36)	(0.44)	(0.50)
Income: £5k or above	1.98***	1.75***	1.81***	1.56***	-0.28	-0.28	0.17	0.21
	(0.47)	(0.46)	(0.40)	(0.46)	(0.28)	(0.28)	(0.37)	(0.37)
Observations	471	469	533	531	763	761	763	761

Table S27. Weighted and unweighted estimates of time spent (hours per day) on employment-related and housework-related activities, by sociodemographic characteristics. Time spent was calculated using main activities only. Regressions with employment activities as the dependent variable used the subset of individuals who remained employed in both periods of interest, and did not use the Heckman correction for selection. Regressions also controlled for age and pre-pandemic levels in total time spent on the given activity. Robust standard errors are reported in brackets. *** $p < 0.01$, ** $p < 0.05$, * $p < 0.1$.

	Leisure		Leisure		Subsistence		Subsistence	
	Lockdown 1		Lockdown 3		Lockdown 1		Lockdown 3	
	Unweighted	Weighted	Unweighted	Weighted	Unweighted	Weighted	Unweighted	Weighted
Female	-0.06	-0.30	-0.38*	-0.11	-0.14	-0.08	0.06	-0.10
	(0.20)	(0.26)	(0.21)	(0.26)	(0.15)	(0.19)	(0.14)	(0.18)
Has young child	-0.94***	-0.87*	-0.92***	-1.03***	-0.48*	-0.55	-0.24	-0.31
	(0.30)	(0.47)	(0.30)	(0.36)	(0.25)	(0.42)	(0.26)	(0.35)
Female x Has young child	-0.29	-0.47	-0.21	-0.60	0.23	0.49	-0.09	-0.05
	(0.41)	(0.53)	(0.40)	(0.44)	(0.34)	(0.47)	(0.33)	(0.38)
WFH during LD1	-0.71***	-0.67***			-0.15	-0.45**		
	(0.18)	(0.24)			(0.14)	(0.20)		
WFH during LD3			-0.14	0.10			-0.00	-0.19
			(0.17)	(0.24)			(0.13)	(0.17)
Has tertiary degree	-0.09	-0.23	0.15	0.27	0.04	-0.02	0.04	-0.07
	(0.19)	(0.26)	(0.20)	(0.26)	(0.14)	(0.21)	(0.15)	(0.19)
White	0.61**	0.39	0.60**	0.55**	-0.10	-0.11	-0.17	-0.16
	(0.25)	(0.33)	(0.24)	(0.27)	(0.19)	(0.21)	(0.20)	(0.25)
Income: £1k-2k	-0.20	-0.36	-0.26	-0.26	-0.10	-0.19	-0.16	-0.34
	(0.23)	(0.32)	(0.24)	(0.31)	(0.18)	(0.22)	(0.18)	(0.23)
Income: 2k-3k	-0.16	-0.13	-0.34	-0.24	-0.10	-0.08	-0.19	-0.38
	(0.27)	(0.37)	(0.27)	(0.32)	(0.21)	(0.26)	(0.19)	(0.26)
Income: £3k-4k	0.07	-0.02	-0.22	-0.33	0.12	0.22	-0.26	-0.37
	(0.32)	(0.40)	(0.33)	(0.40)	(0.24)	(0.27)	(0.23)	(0.26)
Income: £4k-5k	-0.12	-0.58	-0.34	0.44	-0.33	-0.13	-0.99***	-1.76**
	(0.36)	(0.43)	(0.54)	(0.97)	(0.34)	(0.38)	(0.33)	(0.76)
Income: £5k or above	0.24	0.13	-0.34	-0.31	-0.77**	-0.64*	-0.59	-0.70*
	(0.50)	(0.50)	(0.45)	(0.47)	(0.33)	(0.33)	(0.39)	(0.43)
Observations	763	761	763	761	763	761	763	761

Table S28. Weighted and unweighted estimates of time spent (hours per day) on leisure and subsistence activities, by sociodemographic characteristics. Time spent was calculated using main activities only. Regressions also controlled for age and pre-pandemic levels in total time spent on the given activity. Robust standard errors are reported in brackets. *** $p < 0.01$, ** $p < 0.05$, * $p < 0.1$.

7.3 Quality measures: Inverse probability weights

Subgroup	Timepoint	Unweighted Mean	Unweighted 95% CI	Weighted Mean	Weighted 95% CI
**Levels (Hours)**					
Men	Pre	4.19	[3.9, 4.48]	4.04	[3.69, 4.39]
Men	LD 1	4.91	[4.54, 5.27]	4.67	[4.24, 5.1]
Men	LD 3	4.09	[3.76, 4.41]	4.21	[3.78, 4.64]
Women	Pre	4.61	[4.32, 4.91]	4.55	[4.21, 4.9]
Women	LD 1	5.29	[4.94, 5.64]	5.08	[4.67, 5.49]
Women	LD 3	4.40	[4.08, 4.72]	4.32	[3.99, 4.66]
No young child	Pre	4.31	[4.08, 4.53]	4.14	[3.87, 4.41]
No young child	LD 1	4.81	[4.55, 5.08]	4.53	[4.24, 4.83]
No young child	LD 3	4.11	[3.87, 4.36]	4.15	[3.86, 4.44]
Has young child	Pre	4.89	[4.38, 5.41]	4.71	[4.07, 5.35]
Has young child	LD 1	6.51	[5.84, 7.19]	6.05	[5.1, 7]
Has young child	LD 3	4.89	[4.29, 5.5]	4.68	[3.87, 5.5]
**Differences (Hours)**					
Men	Pre vs LD 1	0.72	[0.39, 1.04]	0.63	[0.24, 1.01]
Men	Pre vs LD 3	-0.10	[-0.47, 0.26]	0.17	[-0.26, 0.6]
Women	Pre vs LD 1	0.68	[0.37, 0.99]	0.52	[0.16, 0.88]
Women	Pre vs LD 3	-0.22	[-0.59, 0.15]	-0.23	[-0.62, 0.16]
No young child	Pre vs LD 1	0.50	[0.26, 0.74]	0.39	[0.11, 0.67]
No young child	Pre vs LD 3	-0.20	[-0.47, 0.08]	0.01	[-0.31, 0.32]
Has young child	Pre vs LD 1	1.62	[1.04, 2.2]	1.33	[0.58, 2.09]
Has young child	Pre vs LD 3	0.00	[-0.7, 0.7]	-0.03	[-0.81, 0.75]

Table S29. Weighted and unweighted mean values and within-person differences in multitasking, by timepoint and demographic subgroup. Inverse probability weights were used to construct weighted averages. 'Pre-pandemic' refers to February 2020, 'LD 1' refers to the first lockdown (May 2020), 'LD 3' refers to the third lockdown (March 2021). 95% confidence intervals are reported in brackets. Note that the conditional means were calculated separately (either by gender or household composition), so the four subgroups shown are not mutually exclusive.

Subgroup	Timepoint	Unweighted Mean	Unweighted 95% CI	Weighted Mean	Weighted 95% CI
**Levels (Hours)**					
Men	Pre	1.76	[1.52, 2]	1.77	[1.5, 2.03]
Men	LD 1	2.33	[2.03, 2.63]	2.55	[2.16, 2.93]
Men	LD 3	2.66	[2.36, 2.97]	2.45	[2.08, 2.83]
Women	Pre	1.59	[1.4, 1.79]	1.57	[1.34, 1.8]
Women	LD 1	2.35	[2.09, 2.62]	2.35	[2.06, 2.65]
Women	LD 3	2.65	[2.38, 2.92]	2.58	[2.27, 2.9]
No young child	Pre	1.89	[1.71, 2.07]	1.95	[1.74, 2.16]
No young child	LD 1	2.68	[2.45, 2.91]	2.89	[2.6, 3.18]
No young child	LD 3	3.00	[2.77, 3.22]	2.90	[2.61, 3.19]
Has young child	Pre	0.64	[0.47, 0.82]	0.63	[0.44, 0.83]
Has young child	LD 1	0.74	[0.54, 0.94]	0.80	[0.47, 1.12]
Has young child	LD 3	1.03	[0.79, 1.27]	0.97	[0.65, 1.28]
**Differences (Hours)**					
Men	Pre vs LD 1	0.57	[0.35, 0.79]	0.78	[0.47, 1.08]
Men	Pre vs LD 3	0.90	[0.65, 1.15]	0.68	[0.3, 1.06]
Women	Pre vs LD 1	0.76	[0.55, 0.97]	0.78	[0.53, 1.03]
Women	Pre vs LD 3	1.06	[0.82, 1.29]	1.01	[0.68, 1.35]
No young child	Pre vs LD 1	0.79	[0.61, 0.96]	0.94	[0.7, 1.17]
No young child	Pre vs LD 3	1.11	[0.91, 1.3]	0.95	[0.63, 1.26]
Has young child	Pre vs LD 1	0.10	[-0.11, 0.31]	0.16	[-0.17, 0.5]
Has young child	Pre vs LD 3	0.39	[0.12, 0.66]	0.34	[-0.02, 0.69]

Table S30. Weighted and unweighted mean values and within-person differences in leisure time spent alone, by timepoint and demographic subgroup. Inverse probability weights were used to construct weighted averages. ‘Pre-pandemic’ refers to February 2020, ‘LD 1’ refers to the first lockdown (May 2020), ‘LD 3’ refers to the third lockdown (March 2021). 95% confidence intervals are reported in brackets. Note that the conditional means were calculated separately (either by gender or household composition), so the four subgroups shown are not mutually exclusive.

		Unweighted		Weighted	
**Subgroup**	**Timepoint**	Mean	95% CI	Mean	95% CI
**Levels (% of individuals)**					
Men	Pre	21.25	[17.05, 25.46]	19.62	[14.35, 24.89]
Men	LD 1	15.80	[12.05, 19.55]	13.51	[9.21, 17.82]
Men	LD 3	23.98	[19.59, 28.37]	22.84	[17.29, 28.39]
Women	Pre	22.67	[18.53, 26.81]	23.11	[18.16, 28.06]
Women	LD 1	22.67	[18.53, 26.81]	24.52	[19.35, 29.69]
Women	LD 3	26.20	[21.85, 30.54]	25.94	[20.8, 31.08]
No young child	Pre	23.10	[19.81, 26.4]	22.24	[18.09, 26.39]
No young child	LD 1	19.15	[16.07, 22.22]	18.37	[14.59, 22.15]
No young child	LD 3	25.32	[21.92, 28.72]	24.46	[20.17, 28.75]
Has young child	Pre	16.67	[10.23, 23.11]	16.67	[8.62, 24.72]
Has young child	LD 1	20.45	[13.48, 27.43]	17.58	[10.11, 25.06]
Has young child	LD 3	24.24	[16.84, 31.65]	23.00	[14.06, 31.94]
**Differences (% of individuals)**					
Men	Pre vs LD 1	10.08	[6.99, 13.18]	9.67	[5.85, 13.5]
Men	Pre vs LD 3	17.71	[13.79, 21.64]	17.96	[12.81, 23.1]
Women	Pre vs LD 1	13.85	[10.44, 17.27]	15.54	[11.1, 19.98]
Women	Pre vs LD 3	21.41	[17.36, 25.46]	20.85	[16.11, 25.58]
No young child	Pre vs LD 1	11.87	[9.34, 14.4]	12.23	[8.96, 15.51]
No young child	Pre vs LD 3	19.46	[16.37, 22.56]	18.97	[15.05, 22.9]
Has young child	Pre vs LD 1	12.88	[7.09, 18.67]	11.96	[5.48, 18.44]
Has young child	Pre vs LD 3	20.45	[13.48, 27.43]	20.04	[11.46, 28.61]

Table S31. Weighted and unweighted mean values and within-person differences in unusual work hours, by timepoint and demographic subgroup. Inverse probability weights were used to construct weighted averages. 'Pre-pandemic' refers to February 2020, 'LD 1' refers to the first lockdown (May 2020), 'LD 3' refers to the third lockdown (March 2021). 95% confidence intervals are reported in brackets. Note that the conditional means were calculated separately (either by gender or household composition), so the four subgroups shown are not mutually exclusive.

		Unweighted		Weighted	
**Subgroup**	**Timepoint**	Mean	95% CI	Mean	95% CI
**Levels (% of individuals)**					
Men	Pre	17.98	[14.04, 21.93]	13.97	[9.73, 18.21]
Men	LD 1	27.25	[22.67, 31.82]	23.17	[17.68, 28.67]
Men	LD 3	29.16	[24.48, 33.83]	28.90	[22.86, 34.94]
Women	Pre	28.97	[24.49, 33.45]	26.21	[21.21, 31.21]
Women	LD 1	30.48	[25.93, 35.03]	27.64	[22.66, 32.62]
Women	LD 3	34.51	[29.81, 39.21]	31.87	[26.54, 37.21]
No young child	Pre	21.99	[18.76, 25.23]	16.60	[13.3, 19.89]
No young child	LD 1	26.27	[22.83, 29.71]	22.44	[18.42, 26.46]
No young child	LD 3	28.48	[24.95, 32.01]	26.21	[21.89, 30.54]
Has young child	Pre	31.82	[23.77, 39.87]	29.41	[19.8, 39.02]
Has young child	LD 1	41.67	[33.15, 50.19]	35.47	[25.35, 45.59]
Has young child	LD 3	48.48	[39.85, 57.12]	45.73	[34.7, 56.75]
**Differences (% of individuals)**					
Men	Pre vs LD 1	23.71	[19.33, 28.08]	20.43	[15.13, 25.72]
Men	Pre vs LD 3	25.89	[21.38, 30.39]	27.03	[21.06, 33]
Women	Pre vs LD 1	21.91	[17.83, 26]	19.87	[15.54, 24.19]
Women	Pre vs LD 3	26.95	[22.57, 31.34]	24.53	[19.67, 29.39]
No young child	Pre vs LD 1	20.89	[17.71, 24.06]	18.43	[14.64, 22.21]
No young child	Pre vs LD 3	23.58	[20.26, 26.89]	22.74	[18.55, 26.92]
Has young child	Pre vs LD 1	31.82	[23.77, 39.87]	27.10	[17.83, 36.37]
Has young child	Pre vs LD 3	40.15	[31.68, 48.62]	38.66	[27.98, 49.34]

Table S32. Weighted and unweighted mean values and within-person differences in unusual housework hours, by timepoint and demographic subgroup. Inverse probability weights were used to construct weighted averages. 'Pre-pandemic' refers to February 2020, 'LD 1' refers to the first lockdown (May 2020), 'LD 3' refers to the third lockdown (March 2021). 95% confidence intervals are reported in brackets. Note that the conditional means were calculated separately (either by gender or household composition), so the four subgroups shown are not mutually exclusive.

7.4 Overall enjoyment: Inverse probability weights

		Unweighted		Weighted	
**Subgroup**	**Timepoint**	Mean	95% CI	Mean	95% CI
**Levels (Self-reported enjoyment)**					
Men	Pre	5.57	[5.48, 5.65]	5.63	[5.53, 5.73]
Men	LD 1	5.61	[5.52, 5.7]	5.70	[5.59, 5.82]
Men	LD 3	5.28	[5.18, 5.39]	5.36	[5.25, 5.47]
Women	Pre	5.71	[5.64, 5.78]	5.69	[5.61, 5.78]
Women	LD 1	5.65	[5.57, 5.74]	5.63	[5.53, 5.73]
Women	LD 3	5.44	[5.35, 5.52]	5.43	[5.33, 5.53]
No young child	Pre	5.61	[5.55, 5.67]	5.61	[5.53, 5.68]
No young child	LD 1	5.60	[5.53, 5.67]	5.60	[5.52, 5.69]
No young child	LD 3	5.35	[5.28, 5.43]	5.36	[5.27, 5.44]
Has young child	Pre	5.77	[5.66, 5.88]	5.86	[5.75, 5.98]
Has young child	LD 1	5.76	[5.63, 5.9]	5.94	[5.79, 6.09]
Has young child	LD 3	5.41	[5.27, 5.55]	5.51	[5.37, 5.65]
**Differences (Self-reported enjoyment)**					
Men	Pre vs LD 1	0.05	[-0.01, 0.1]	0.07	[-0.01, 0.15]
Men	Pre vs LD 3	-0.28	[-0.39, -0.18]	-0.27	[-0.39, -0.16]
Women	Pre vs LD 1	-0.06	[-0.12, 0]	-0.07	[-0.14, 0.01]
Women	Pre vs LD 3	-0.27	[-0.36, -0.19]	-0.27	[-0.36, -0.18]
No young child	Pre vs LD 1	-0.01	[-0.06, 0.04]	0.00	[-0.06, 0.06]
No young child	Pre vs LD 3	-0.26	[-0.34, -0.18]	-0.25	[-0.34, -0.16]
Has young child	Pre vs LD 1	-0.01	[-0.1, 0.09]	0.08	[-0.07, 0.22]
Has young child	Pre vs LD 3	-0.36	[-0.48, -0.24]	-0.36	[-0.49, -0.23]

Table S33. Weighted and unweighted estimates of mean levels and within-person differences in overall enjoyment, by timepoint and demographic subgroup. ‘Pre’ refers to pre-pandemic (February 2020), ‘LD 1’ refers to the first lockdown (May 2020), ‘LD 3’ refers to the third lockdown (March 2021). 95% confidence intervals are reported in brackets. Note that the conditional means were calculated separately (either by gender or household composition), so the four subgroups shown are not mutually exclusive.

	Change in overall enjoyment (LD1 vs Pre)		Change in overall enjoyment (LD3 vs Pre)	
	**Unweighted**	**Weighted**	**Unweighted**	**Weighted**
**A. Quantity: Changes (hours per day) spent on:**				
Housework	0.01	-0.00	0.04*	0.05**
	(0.02)	(0.02)	(0.02)	(0.02)
Employment	-0.00	-0.02	0.02	0.01
	(0.01)	(0.02)	(0.02)	(0.02)
Leisure	0.07***	0.07***	0.07***	0.08***
	(0.02)	(0.02)	(0.02)	(0.02)
Subsistence	0.04**	0.02	0.03	0.04*
	(0.02)	(0.03)	(0.02)	(0.02)
**B. Quality: Changes (hours per day) spent on:**				
Multitasking across categories	0.01*	0.01	0.01	0.01
	(0.01)	(0.01)	(0.01)	(0.01)
Leisure activity done alone	-0.03**	-0.02	-0.04**	-0.05**
	(0.01)	(0.02)	(0.02)	(0.02)
Unusual work hours	-0.02	-0.04	-0.08***	-0.08***
	(0.02)	(0.03)	(0.03)	(0.03)
Unusual housework hours	-0.01	-0.03	-0.04	-0.07
	(0.03)	(0.04)	(0.05)	(0.05)
**C. Sociodemographic covariates**				
WFH during LD1	0.14**	0.21***		
	(0.06)	(0.07)		
WFH during LD3			0.06	0.10
			(0.07)	(0.07)
Female	-0.13**	-0.12**	0.07	0.03
	(0.05)	(0.06)	(0.08)	(0.09)
Has young child	0.01	0.14	0.04	-0.01
	(0.08)	(0.10)	(0.12)	(0.12)
Female X Has young child	0.09	-0.04	-0.19	-0.14
	(0.11)	(0.13)	(0.14)	(0.15)
Has tertiary degree	-0.03	-0.04	0.14**	0.09
	(0.05)	(0.06)	(0.07)	(0.07)
White	-0.10	-0.10	-0.11	-0.10
	(0.07)	(0.07)	(0.10)	(0.11)
Income: £1k-2k	-0.06	-0.10	-0.17*	-0.20*
	(0.06)	(0.07)	(0.09)	(0.10)
Income: £2k-3k	-0.15**	-0.13	-0.22**	-0.29**
	(0.07)	(0.09)	(0.11)	(0.13)
Income: £3k-4k	0.01	0.03	-0.03	-0.08
	(0.07)	(0.08)	(0.13)	(0.13)
Income: £4k-5k	-0.00	-0.05	0.04	0.04
	(0.10)	(0.10)	(0.16)	(0.19)
Income: £5k or above	-0.11	-0.14	0.22	0.16
	(0.16)	(0.14)	(0.16)	(0.17)
Baseline mean enjoyment	5.64	5.64	5.64	5.64
Baseline SD enjoyment	0.76	0.76	0.76	0.76
Baseline mean change in enjoyment	-0.01	-0.01	-0.28	-0.28
Baseline SD change in enjoyment	0.61	0.61	0.94	0.94
Observations	762	760	761	759

Table S34. Weighted and unweighted estimates of correlations between within-person changes in overall self-reported enjoyment and characteristics of time use. Sociodemographic covariates were used as controls. Reported changes during the first and third lockdown (May 2020 and March 2021, respectively) were relative to the pre-pandemic timepoint (February 2020). Robust standard errors are reported in brackets. A coefficient of 0.1 corresponds to ~0.13 SD in pre-pandemic enjoyment levels. *** $p < 0.01$, ** $p < 0.05$, * $p < 0.1$.

8 Sample representativeness

In Section 2.3, we found evidence that our sample is, on average, more educated and older than a nationally representative sample of the UK workforce. To address potential concerns about sample representativeness, we used calibration weights to reweight our sample to match the composition of Understanding Society's in-workforce sample across gender, age, ethnicity, education, and household composition (all defined as categorical variables).

Specifically, let j denote a vector of K binary characteristics, where the kth element equals 1 if the respondent satisfies the condition of characteristic k. Since the characteristics are binary, there is a finite number of possible combinations of these vectors, denoted J.

Let $w_{s,j}$ denote the weight currently assigned to an individual with the characteristic vector j, which is calculated by dividing the number of individuals with these characteristics by the total sample size (the proportion p_j). We chose the calibration weights $\omega_{c,j}$ to be as 'close as possible' (in the squared-distance sense) to the original weights $w_{s,j}$, such that the re-weighted proportions equal those of the nationally representative sample $p_{RS,j}$:

$$\min_{\omega_{c,1},\ldots,\omega_{c,J}} \sum_{j=1}^{J} (\omega_{c,j} - w_{s,j})^2$$
$$\text{subject to } \omega_{c,j} p_j = p_{RS,j} \text{ for } j = 1, \ldots, J$$

Tables S35-S46 compare the weighted and unweighted results from Figures 1-4 in our main manuscript. Again, our results were qualitatively similar.

8.1 Quantity measures: Calibration weights

		Unweighted		Weighted	
**Subgroup**	**Timepoint**	Mean	95% CI	Mean	95% CI
**Levels (Hours)**					
Men	Pre	7.70	[7.46, 7.94]	7.74	[7.47, 8]
Men	LD 1	7.26	[6.98, 7.55]	7.30	[6.96, 7.64]
Men	LD 3	7.41	[7.18, 7.65]	7.44	[7.18, 7.7]
Women	Pre	6.84	[6.58, 7.1]	6.65	[6.34, 6.96]
Women	LD 1	6.55	[6.25, 6.85]	6.43	[6.07, 6.78]
Women	LD 3	6.83	[6.56, 7.11]	6.73	[6.39, 7.07]
No young child	Pre	7.24	[7.04, 7.44]	7.23	[6.99, 7.47]
No young child	LD 1	6.91	[6.68, 7.15]	6.93	[6.65, 7.21]
No young child	LD 3	7.17	[6.97, 7.37]	7.21	[6.98, 7.45]
Has young child	Pre	7.41	[7.02, 7.8]	7.22	[6.78, 7.65]
Has young child	LD 1	6.92	[6.45, 7.38]	6.85	[6.31, 7.39]
Has young child	LD 3	6.98	[6.59, 7.38]	6.89	[6.43, 7.36]
**Differences (Hours)**					
Men	Pre vs LD 1	-0.54	[-0.82, -0.26]	-0.50	[-0.79, -0.21]
Men	Pre vs LD 3	-0.51	[-0.81, -0.22]	-0.52	[-0.81, -0.23]
Women	Pre vs LD 1	-0.48	[-0.78, -0.19]	-0.38	[-0.76, 0]
Women	Pre vs LD 3	-0.28	[-0.6, 0.03]	-0.24	[-0.64, 0.17]
No young child	Pre vs LD 1	-0.46	[-0.67, -0.24]	-0.37	[-0.61, -0.14]
No young child	Pre vs LD 3	-0.37	[-0.61, -0.13]	-0.36	[-0.62, -0.09]
Has young child	Pre vs LD 1	-0.72	[-1.23, -0.21]	-0.61	[-1.15, -0.06]
Has young child	Pre vs LD 3	-0.53	[-0.97, -0.1]	-0.49	[-1.01, 0.04]

Table S35. Weighted and unweighted mean time spent and within-person differences in time spent (hours per day) on employment-related activities, by timepoint. Calibration weights were used to construct weighted

averages. ‘Pre-pandemic’ refers to February 2020, ‘LD 1’ refers to the first lockdown (May 2020), ‘LD 3’ refers to the third lockdown (March 2021). Within-person changes for employment activities were calculated using the subset of individuals who remained employed in both periods of interest. 95% confidence intervals are reported in brackets. Note that the conditional means were calculated separately (either by gender or household composition), so the four subgroups shown are not mutually exclusive.

Subgroup	Timepoint	Unweighted		Weighted	
		Mean	95% CI	Mean	95% CI
**Levels (Hours)**					
Men	Pre	2.77	[2.55, 2.99]	2.90	[2.63, 3.16]
Men	LD 1	3.47	[3.18, 3.75]	3.67	[3.32, 4.02]
Men	LD 3	3.00	[2.77, 3.23]	3.02	[2.75, 3.3]
Women	Pre	3.38	[3.14, 3.61]	3.71	[3.4, 4.02]
Women	LD 1	3.84	[3.59, 4.1]	4.20	[3.86, 4.55]
Women	LD 3	3.54	[3.31, 3.76]	3.95	[3.6, 4.3]
No young child	Pre	2.73	[2.57, 2.9]	2.76	[2.56, 2.96]
No young child	LD 1	3.23	[3.03, 3.43]	3.30	[3.04, 3.55]
No young child	LD 3	2.92	[2.75, 3.08]	2.89	[2.68, 3.09]
Has young child	Pre	4.77	[4.36, 5.18]	4.91	[4.45, 5.37]
Has young child	LD 1	5.74	[5.32, 6.16]	5.83	[5.36, 6.31]
Has young child	LD 3	5.01	[4.61, 5.42]	5.27	[4.75, 5.79]
**Differences (Hours)**					
Men	Pre vs LD 1	0.70	[0.49, 0.91]	0.77	[0.51, 1.04]
Men	Pre vs LD 3	0.23	[0, 0.47]	0.12	[-0.15, 0.4]
Women	Pre vs LD 1	0.47	[0.29, 0.64]	0.49	[0.28, 0.71]
Women	Pre vs LD 3	0.16	[-0.07, 0.39]	0.24	[-0.09, 0.57]
No young child	Pre vs LD 1	0.50	[0.35, 0.64]	0.54	[0.34, 0.73]
No young child	Pre vs LD 3	0.18	[0.01, 0.36]	0.12	[-0.09, 0.34]
Has young child	Pre vs LD 1	0.97	[0.61, 1.33]	0.92	[0.56, 1.28]
Has young child	Pre vs LD 3	0.24	[-0.23, 0.71]	0.36	[-0.22, 0.94]

Table S36. Weighted and unweighted mean time spent and within-person differences in time spent (hours per day) on housework-related activities, by timepoint. Calibration weights were used to construct weighted averages. ‘Pre-pandemic’ refers to February 2020, ‘LD 1’ refers to the first lockdown (May 2020), ‘LD 3’ refers to the third lockdown (March 2021). 95% confidence intervals are reported in brackets. Note that the conditional means were calculated separately (either by gender or household composition), so the four subgroups shown are not mutually exclusive.

Subgroup	Timepoint	Unweighted		Weighted	
		Mean	95% CI	Mean	95% CI
**Levels (Hours)**					
Men	Pre	5.22	[4.97, 5.48]	5.16	[4.89, 5.44]
Men	LD 1	5.71	[5.42, 6]	5.65	[5.3, 6]
Men	LD 3	6.10	[5.81, 6.38]	6.01	[5.69, 6.34]
Women	Pre	4.72	[4.48, 4.96]	4.42	[4.13, 4.71]
Women	LD 1	5.36	[5.09, 5.63]	5.05	[4.71, 5.39]
Women	LD 3	5.63	[5.37, 5.9]	5.37	[5.04, 5.71]
No young child	Pre	5.25	[5.05, 5.44]	5.21	[4.98, 5.43]
No young child	LD 1	5.88	[5.67, 6.09]	5.87	[5.61, 6.14]
No young child	LD 3	6.22	[6.01, 6.44]	6.26	[6, 6.53]
Has young child	Pre	3.59	[3.25, 3.92]	3.57	[3.22, 3.92]
Has young child	LD 1	3.85	[3.45, 4.24]	3.80	[3.35, 4.26]
Has young child	LD 3	4.11	[3.77, 4.45]	4.01	[3.64, 4.38]
**Differences (Hours)**					
Men	Pre vs LD 1	0.49	[0.23, 0.75]	0.49	[0.21, 0.77]
Men	Pre vs LD 3	0.87	[0.59, 1.16]	0.85	[0.54, 1.17]
Women	Pre vs LD 1	0.64	[0.4, 0.89]	0.63	[0.34, 0.92]
Women	Pre vs LD 3	0.92	[0.62, 1.21]	0.95	[0.6, 1.3]
No young child	Pre vs LD 1	0.63	[0.43, 0.83]	0.67	[0.43, 0.9]
No young child	Pre vs LD 3	0.97	[0.74, 1.21]	1.06	[0.78, 1.34]
Has young child	Pre vs LD 1	0.26	[-0.13, 0.65]	0.23	[-0.17, 0.63]
Has young child	Pre vs LD 3	0.52	[0.1, 0.94]	0.43	[0.02, 0.85]

Table S37. Weighted and unweighted mean time spent and within-person differences in time spent (hours per day) on leisure activities, by timepoint. Calibration weights were used to construct weighted averages. ‘Pre-pandemic’ refers to February 2020, ‘LD 1’ refers to the first lockdown (May 2020), ‘LD 3’ refers to the third lockdown (March 2021). 95% confidence intervals are reported in brackets. Note that the conditional means were calculated separately (either by gender or household composition), so the four subgroups shown are not mutually exclusive.

		Unweighted		Weighted	
**Subgroup**	**Timepoint**	Mean	95% CI	Mean	95% CI
**Levels (Hours)**					
Men	Pre	11.11	[10.92, 11.3]	11.11	[10.88, 11.34]
Men	LD 1	11.60	[11.38, 11.83]	11.54	[11.28, 11.8]
Men	LD 3	11.11	[10.92, 11.29]	11.14	[10.92, 11.36]
Women	Pre	11.52	[11.31, 11.73]	11.59	[11.31, 11.86]
Women	LD 1	11.83	[11.61, 12.05]	11.84	[11.55, 12.14]
Women	LD 3	11.39	[11.19, 11.59]	11.37	[11.13, 11.61]
No young child	Pre	11.41	[11.26, 11.57]	11.50	[11.31, 11.69]
No young child	LD 1	11.83	[11.66, 12.01]	11.86	[11.65, 12.08]
No young child	LD 3	11.32	[11.17, 11.48]	11.38	[11.2, 11.56]
Has young child	Pre	10.90	[10.55, 11.25]	10.91	[10.48, 11.33]
Has young child	LD 1	11.18	[10.8, 11.55]	11.18	[10.73, 11.63]
Has young child	LD 3	10.91	[10.6, 11.21]	10.87	[10.53, 11.22]
**Differences (Hours)**					
Men	Pre vs LD 1	0.50	[0.31, 0.69]	0.43	[0.21, 0.65]
Men	Pre vs LD 3	0.00	[-0.22, 0.22]	0.03	[-0.23, 0.28]
Women	Pre vs LD 1	0.31	[0.1, 0.51]	0.26	[-0.01, 0.53]
Women	Pre vs LD 3	-0.14	[-0.38, 0.11]	-0.22	[-0.54, 0.1]
No young child	Pre vs LD 1	0.42	[0.27, 0.58]	0.37	[0.18, 0.56]
No young child	Pre vs LD 3	-0.09	[-0.27, 0.09]	-0.12	[-0.34, 0.11]
Has young child	Pre vs LD 1	0.28	[-0.05, 0.61]	0.27	[-0.12, 0.67]
Has young child	Pre vs LD 3	0.01	[-0.38, 0.39]	-0.03	[-0.5, 0.44]

Table S38. Weighted and unweighted mean time spent and within-person differences in time spent (hours per day) on subsistence activities, by timepoint. Calibration weights were used to construct weighted averages. ‘Pre-pandemic’ refers to February 2020, ‘LD 1’ refers to the first lockdown (May 2020), ‘LD 3’ refers to the third lockdown (March 2021). 95% confidence intervals are reported in brackets. Note that the conditional means were calculated separately (either by gender or household composition), so the four subgroups shown are not mutually exclusive.

8.2 Regression tables: Calibration weights

	Employment		Employment		Housework		Housework	
	**Lockdown 1**		**Lockdown 3**		**Lockdown 1**		**Lockdown 3**	
	Unweighted	Weighted	Unweighted	Weighted	Unweighted	Weighted	Unweighted	Weighted
Female	0.07	0.33	0.35	0.64**	-0.18	-0.29	-0.09	0.07
	(0.21)	(0.24)	(0.28)	(0.30)	(0.16)	(0.21)	(0.20)	(0.24)
Has young child	-0.04	0.29	-0.24	0.05	1.12***	0.99***	-0.01	0.08
	(0.34)	(0.36)	(0.34)	(0.39)	(0.29)	(0.35)	(0.38)	(0.47)
Female x Has young child	-1.15**	-1.56***	-0.33	-0.96	0.38	0.58	0.87*	0.84
	(0.49)	(0.54)	(0.59)	(0.64)	(0.38)	(0.41)	(0.51)	(0.59)
WFH during LD1	-0.27	-0.21	0.35	0.35	-0.37***	-0.49***	0.09	-0.12
	(0.24)	(0.28)	(0.37)	(0.37)	(0.14)	(0.17)	(0.19)	(0.23)
WFH during LD3			-0.34	-0.54*			-0.15	-0.18
			(0.25)	(0.28)			(0.18)	(0.22)
Has tertiary degree	-0.21	-0.24	-0.28	-0.49	-0.05	0.02	-0.12	-0.39
	(0.21)	(0.26)	(0.28)	(0.30)	(0.15)	(0.19)	(0.19)	(0.26)
White	-0.42	-0.41	0.25	0.00	0.10	0.16	-0.03	0.11
	(0.26)	(0.27)	(0.34)	(0.40)	(0.17)	(0.19)	(0.23)	(0.26)
Income: £1k-2k	0.85***	1.00***	-0.46	-0.35	0.09	0.15	-0.06	-0.12
	(0.32)	(0.36)	(0.50)	(0.54)	(0.19)	(0.24)	(0.24)	(0.30)
Income: £2k-3k	1.05***	1.01**	0.07	0.31	0.07	0.08	-0.07	-0.43
	(0.33)	(0.40)	(0.48)	(0.56)	(0.21)	(0.28)	(0.26)	(0.34)
Income: £3k-4k	1.34***	1.45***	0.04	-0.51	-0.23	-0.30	0.05	0.37
	(0.37)	(0.43)	(0.54)	(0.64)	(0.24)	(0.27)	(0.38)	(0.44)
Income: £4k-5k	1.16**	1.14**	-0.09	-0.30	-0.16	0.31	-0.09	0.17
	(0.46)	(0.56)	(0.56)	(0.75)	(0.34)	(0.39)	(0.48)	(0.75)
Income: £5k or above	1.98***	1.78***	1.00	0.85	-0.28	-0.07	0.18	-0.25
	(0.47)	(0.54)	(0.65)	(0.63)	(0.28)	(0.40)	(0.42)	(0.54)
Observations	471	437	433	403	763	711	763	711

Table S39. Weighted and unweighted estimates of time spent (hours per day) on employment-related and housework-related activities, by sociodemographic characteristics. Time spent was calculated using main activities only. Regressions with employment activities as the dependent variable used the subset of individuals who remained employed in both periods of interest, and did not use the Heckman correction for selection. Regressions also controlled for age and pre-pandemic levels in total time spent on the given activity. Robust standard errors are reported in brackets. *** $p < 0.01$, ** $p < 0.05$, * $p < 0.1$.

	Leisure		Leisure		Subsistence		Subsistence	
	**Lockdown 1**		**Lockdown 3**		**Lockdown 1**		**Lockdown 3**	
	Unweighted	Weighted	Unweighted	Weighted	Unweighted	Weighted	Unweighted	Weighted
Female	-0.06	-0.06	-0.08	-0.06	-0.14	-0.11	-0.18	-0.29
	(0.20)	(0.23)	(0.25)	(0.30)	(0.15)	(0.19)	(0.18)	(0.23)
Has young child	-0.94***	-0.77**	-0.04	-0.33	-0.48*	-0.33	0.02	0.22
	(0.30)	(0.35)	(0.36)	(0.41)	(0.25)	(0.30)	(0.32)	(0.43)
Female x Has young child	-0.29	-0.12	-0.20	-0.23	0.23	-0.00	-0.18	-0.26
	(0.41)	(0.44)	(0.50)	(0.52)	(0.34)	(0.40)	(0.42)	(0.53)
WFH during LD1	-0.71***	-0.58***	-0.69***	-0.58*	-0.15	-0.30*	0.16	0.29
	(0.18)	(0.21)	(0.26)	(0.31)	(0.14)	(0.17)	(0.18)	(0.22)
WFH during LD3			0.15	0.23			-0.14	-0.10
			(0.24)	(0.28)			(0.18)	(0.21)
Has tertiary degree	-0.09	-0.17	0.25	0.47	0.04	0.07	0.12	0.17
	(0.19)	(0.24)	(0.24)	(0.29)	(0.14)	(0.18)	(0.18)	(0.23)
White	0.61**	0.63**	0.28	0.51*	-0.10	-0.17	0.03	-0.29
	(0.25)	(0.27)	(0.29)	(0.30)	(0.19)	(0.21)	(0.24)	(0.28)
Income: £1k-2k	-0.20	-0.28	-0.09	-0.42	-0.10	-0.22	0.01	0.02
	(0.23)	(0.27)	(0.30)	(0.35)	(0.18)	(0.22)	(0.23)	(0.28)
Income: £2k-3k	-0.16	0.01	-0.10	-0.10	-0.10	-0.34	-0.20	-0.30
	(0.27)	(0.33)	(0.34)	(0.39)	(0.21)	(0.28)	(0.26)	(0.31)
Income: £3k-4k	0.07	0.32	0.08	-0.13	0.12	-0.11	-0.15	-0.20
	(0.32)	(0.40)	(0.42)	(0.47)	(0.24)	(0.29)	(0.28)	(0.33)
Income: £4k-5k	-0.12	-0.69*	-0.18	-0.49	-0.33	-0.46	-1.03**	-1.21***
	(0.36)	(0.35)	(0.56)	(0.76)	(0.34)	(0.32)	(0.40)	(0.46)
Income: £5k or above	0.24	0.26	0.27	-0.02	-0.77**	-0.78	-0.90*	-0.78
	(0.50)	(0.63)	(0.58)	(0.77)	(0.33)	(0.49)	(0.52)	(0.66)
Observations	763	711	763	711	763	711	763	711

Table S40. Weighted and unweighted estimates of time spent (hours per day) on leisure and subsistence activities, by sociodemographic characteristics. Time spent was calculated using main activities only. Regressions also controlled for age and pre-pandemic levels in total time spent on the given activity. Robust standard errors are reported in brackets. *** p < 0.01, ** p < 0.05, * p < 0.1.

8.3 Quality measures: Calibration weights

Subgroup	Timepoint	Unweighted		Weighted	
		Mean	95% CI	Mean	95% CI
**Levels (Hours)**					
Men	Pre	4.19	[3.9, 4.48]	4.30	[3.94, 4.65]
Men	LD 1	4.91	[4.54, 5.27]	4.98	[4.56, 5.4]
Men	LD 3	4.09	[3.76, 4.41]	4.17	[3.79, 4.54]
Women	Pre	4.61	[4.32, 4.91]	4.70	[4.32, 5.09]
Women	LD 1	5.29	[4.94, 5.64]	5.41	[4.96, 5.86]
Women	LD 3	4.40	[4.08, 4.72]	4.44	[4.02, 4.86]
No young child	Pre	4.31	[4.08, 4.53]	4.37	[4.09, 4.66]
No young child	LD 1	4.81	[4.55, 5.08]	4.74	[4.42, 5.06]
No young child	LD 3	4.11	[3.87, 4.36]	4.03	[3.74, 4.32]
Has young child	Pre	4.89	[4.38, 5.41]	4.87	[4.28, 5.47]
Has young child	LD 1	6.51	[5.84, 7.19]	6.54	[5.82, 7.26]
Has young child	LD 3	4.89	[4.29, 5.5]	5.10	[4.41, 5.79]
**Differences (Hours)**					
Men	Pre vs LD 1	0.72	[0.39, 1.04]	0.68	[0.3, 1.06]
Men	Pre vs LD 3	-0.10	[-0.47, 0.26]	-0.13	[-0.57, 0.31]
Women	Pre vs LD 1	0.68	[0.37, 0.99]	0.71	[0.3, 1.11]
Women	Pre vs LD 3	-0.22	[-0.59, 0.15]	-0.27	[-0.78, 0.25]
No young child	Pre vs LD 1	0.50	[0.26, 0.74]	0.37	[0.07, 0.66]
No young child	Pre vs LD 3	-0.20	[-0.47, 0.08]	-0.34	[-0.7, 0.02]
Has young child	Pre vs LD 1	1.62	[1.04, 2.2]	1.67	[1.03, 2.3]
Has young child	Pre vs LD 3	0.00	[-0.7, 0.7]	0.23	[-0.59, 1.04]

Table S41. Weighted and unweighted mean values and within-person differences in multitasking, by timepoint and demographic subgroup. Calibration weights were used to construct weighted averages. ‘Pre-pandemic’ refers to February 2020, ‘LD 1’ refers to the first lockdown (May 2020), ‘LD 3’ refers to the third lockdown (March 2021). 95% confidence intervals are reported in brackets. Note that the conditional means were calculated separately (either by gender or household composition), so the four subgroups shown are not mutually exclusive.

Subgroup	Timepoint	Unweighted		Weighted	
		Mean	95% CI	Mean	95% CI
**Levels (Hours)**					
Men	Pre	1.76	[1.52, 2]	1.60	[1.34, 1.86]
Men	LD 1	2.33	[2.03, 2.63]	2.11	[1.77, 2.45]
Men	LD 3	2.66	[2.36, 2.97]	2.43	[2.08, 2.78]
Women	Pre	1.59	[1.4, 1.79]	1.38	[1.15, 1.6]
Women	LD 1	2.35	[2.09, 2.62]	1.98	[1.7, 2.26]
Women	LD 3	2.65	[2.38, 2.92]	2.28	[1.98, 2.59]
No young child	Pre	1.89	[1.71, 2.07]	1.77	[1.56, 1.97]
No young child	LD 1	2.68	[2.45, 2.91]	2.50	[2.23, 2.76]
No young child	LD 3	3.00	[2.77, 3.22]	2.83	[2.55, 3.11]
Has young child	Pre	0.64	[0.47, 0.82]	0.67	[0.44, 0.9]
Has young child	LD 1	0.74	[0.54, 0.94]	0.69	[0.46, 0.93]
Has young child	LD 3	1.03	[0.79, 1.27]	0.96	[0.72, 1.19]
**Differences (Hours)**					
Men	Pre vs LD 1	0.57	[0.35, 0.79]	0.51	[0.28, 0.73]
Men	Pre vs LD 3	0.90	[0.65, 1.15]	0.83	[0.55, 1.11]
Women	Pre vs LD 1	0.76	[0.55, 0.97]	0.60	[0.36, 0.84]
Women	Pre vs LD 3	1.06	[0.82, 1.29]	0.90	[0.62, 1.18]
No young child	Pre vs LD 1	0.79	[0.61, 0.96]	0.73	[0.54, 0.93]
No young child	Pre vs LD 3	1.11	[0.91, 1.3]	1.06	[0.82, 1.31]
Has young child	Pre vs LD 1	0.10	[-0.11, 0.31]	0.02	[-0.24, 0.28]
Has young child	Pre vs LD 3	0.39	[0.12, 0.66]	0.28	[0, 0.56]

Table S42. Weighted and unweighted mean values and within-person differences in leisure time spent alone, by timepoint and demographic subgroup. Calibration weights were used to construct weighted averages. 'Pre-pandemic' refers to February 2020, 'LD 1' refers to the first lockdown (May 2020), 'LD 3' refers to the third lockdown (March 2021). 95% confidence intervals are reported in brackets. Note that the conditional means were calculated separately (either by gender or household composition), so the four subgroups shown are not mutually exclusive.

		Unweighted		Weighted	
**Subgroup**	**Timepoint**	Mean	95% CI	Mean	95% CI
**Levels (% of individuals)**					
Men	Pre	21.25	[17.05, 25.46]	19.05	[14.41, 23.69]
Men	LD 1	15.80	[12.05, 19.55]	13.26	[9.27, 17.26]
Men	LD 3	23.98	[19.59, 28.37]	21.55	[16.66, 26.44]
Women	Pre	22.67	[18.53, 26.81]	20.87	[16.11, 25.64]
Women	LD 1	22.67	[18.53, 26.81]	23.12	[18, 28.25]
Women	LD 3	26.20	[21.85, 30.54]	24.31	[19.16, 29.47]
No young child	Pre	23.10	[19.81, 26.4]	21.84	[18.02, 25.66]
No young child	LD 1	19.15	[16.07, 22.22]	17.10	[13.69, 20.52]
No young child	LD 3	25.32	[21.92, 28.72]	22.64	[18.8, 26.48]
Has young child	Pre	16.67	[10.23, 23.11]	14.34	[7.71, 20.96]
Has young child	LD 1	20.45	[13.48, 27.43]	21.28	[13.15, 29.41]
Has young child	LD 3	24.24	[16.84, 31.65]	23.75	[15.41, 32.09]
**Differences (% of individuals)**					
Men	Pre vs LD 1	10.08	[6.99, 13.18]	8.16	[5.02, 11.3]
Men	Pre vs LD 3	17.71	[13.79, 21.64]	15.82	[11.47, 20.17]
Women	Pre vs LD 1	13.85	[10.44, 17.27]	14.06	[9.78, 18.35]
Women	Pre vs LD 3	21.41	[17.36, 25.46]	19.77	[14.96, 24.57]
No young child	Pre vs LD 1	11.87	[9.34, 14.4]	10.37	[7.64, 13.1]
No young child	Pre vs LD 3	19.46	[16.37, 22.56]	16.89	[13.46, 20.32]
Has young child	Pre vs LD 1	12.88	[7.09, 18.67]	13.23	[6.42, 20.05]
Has young child	Pre vs LD 3	20.45	[13.48, 27.43]	20.42	[12.52, 28.31]

Table S43. Weighted and unweighted mean values and within-person differences in unusual work hours, by timepoint and demographic subgroup. Calibration weights were used to construct weighted averages. 'Pre-pandemic' refers to February 2020, 'LD 1' refers to the first lockdown (May 2020), 'LD 3' refers to the third lockdown (March 2021). 95% confidence intervals are reported in brackets. Note that the conditional means were calculated separately (either by gender or household composition), so the four subgroups shown are not mutually exclusive.

		Unweighted		Weighted	
**Subgroup**	**Timepoint**	Mean	95% CI	Mean	95% CI
**Levels (% of individuals)**					
Men	Pre	17.98	[14.04, 21.93]	16.67	[12.12, 21.21]
Men	LD 1	27.25	[22.67, 31.82]	27.77	[22.29, 33.26]
Men	LD 3	29.16	[24.48, 33.83]	29.94	[24.37, 35.51]
Women	Pre	28.97	[24.49, 33.45]	29.79	[24.05, 35.53]
Women	LD 1	30.48	[25.93, 35.03]	29.16	[23.55, 34.77]
Women	LD 3	34.51	[29.81, 39.21]	33.86	[28, 39.71]
No young child	Pre	21.99	[18.76, 25.23]	19.44	[15.72, 23.15]
No young child	LD 1	26.27	[22.83, 29.71]	23.95	[19.96, 27.95]
No young child	LD 3	28.48	[24.95, 32.01]	25.59	[21.57, 29.62]
Has young child	Pre	31.82	[23.77, 39.87]	34.32	[24.87, 43.78]
Has young child	LD 1	41.67	[33.15, 50.19]	41.91	[32.2, 51.63]
Has young child	LD 3	48.48	[39.85, 57.12]	50.63	[40.79, 60.47]
**Differences (% of individuals)**					
Men	Pre vs LD 1	23.71	[19.33, 28.08]	24.30	[19.05, 29.55]
Men	Pre vs LD 3	25.89	[21.38, 30.39]	27.04	[21.64, 32.44]
Women	Pre vs LD 1	21.91	[17.83, 26]	21.35	[16.25, 26.45]
Women	Pre vs LD 3	26.95	[22.57, 31.34]	25.61	[20.12, 31.11]
No young child	Pre vs LD 1	20.89	[17.71, 24.06]	19.71	[15.97, 23.46]
No young child	Pre vs LD 3	23.58	[20.26, 26.89]	20.61	[16.9, 24.33]
Has young child	Pre vs LD 1	31.82	[23.77, 39.87]	32.15	[22.94, 41.36]
Has young child	Pre vs LD 3	40.15	[31.68, 48.62]	43.40	[33.59, 53.21]

Table S44. Weighted and unweighted mean values and within-person differences in unusual housework hours, by timepoint and demographic subgroup. Calibration weights were used to construct weighted averages. 'Pre-pandemic' refers to February 2020, 'LD 1' refers to the first lockdown (May 2020), 'LD 3' refers to the third lockdown (March 2021). 95% confidence intervals are reported in brackets. Note that the conditional means were calculated separately (either by gender or household composition), so the four subgroups shown are not mutually exclusive.

8.4 Overall enjoyment: Calibration weights

		Unweighted		Weighted	
**Subgroup**	**Timepoint**	Mean	95% CI	Mean	95% CI
**Levels (Self-reported enjoyment)**					
Men	Pre	5.57	[5.48, 5.65]	5.57	[5.49, 5.66]
Men	LD 1	5.61	[5.52, 5.7]	5.63	[5.53, 5.73]
Men	LD 3	5.28	[5.18, 5.39]	5.20	[5.07, 5.33]
Women	Pre	5.71	[5.64, 5.78]	5.72	[5.63, 5.8]
Women	LD 1	5.65	[5.57, 5.74]	5.65	[5.54, 5.75]
Women	LD 3	5.44	[5.35, 5.52]	5.43	[5.33, 5.53]
No young child	Pre	5.61	[5.55, 5.67]	5.62	[5.54, 5.69]
No young child	LD 1	5.60	[5.53, 5.67]	5.62	[5.54, 5.71]
No young child	LD 3	5.35	[5.28, 5.43]	5.30	[5.21, 5.4]
Has young child	Pre	5.77	[5.66, 5.88]	5.72	[5.61, 5.84]
Has young child	LD 1	5.76	[5.63, 5.9]	5.68	[5.53, 5.83]
Has young child	LD 3	5.41	[5.27, 5.55]	5.34	[5.18, 5.49]
**Differences (Self-reported enjoyment)**					
Men	Pre vs LD 1	0.05	[-0.01, 0.1]	0.06	[-0.01, 0.13]
Men	Pre vs LD 3	-0.28	[-0.39, -0.18]	-0.37	[-0.51, -0.24]
Women	Pre vs LD 1	-0.06	[-0.12, 0]	-0.07	[-0.15, 0.01]
Women	Pre vs LD 3	-0.27	[-0.36, -0.19]	-0.29	[-0.38, -0.19]
No young child	Pre vs LD 1	-0.01	[-0.06, 0.04]	0.01	[-0.05, 0.06]
No young child	Pre vs LD 3	-0.26	[-0.34, -0.18]	-0.31	[-0.41, -0.21]
Has young child	Pre vs LD 1	-0.01	[-0.1, 0.09]	-0.04	[-0.16, 0.08]
Has young child	Pre vs LD 3	-0.36	[-0.48, -0.24]	-0.39	[-0.53, -0.24]

Table S45. Weighted and unweighted estimates of mean levels and within-person differences in overall enjoyment, by timepoint and demographic subgroup. 'Pre' refers to pre-pandemic (February 2020), 'LD 1' refers to the first lockdown (May 2020), 'LD 3' refers to the third lockdown (March 2021). 95% confidence intervals are reported in brackets. Note that the conditional means were calculated separately (either by gender or household composition), so the four subgroups shown are not mutually exclusive.

	Change in overall enjoyment (LD1 vs Pre)		Change in overall enjoyment (LD3 vs Pre)	
	**Unweighted**	**Weighted**	**Unweighted**	**Weighted**
**A. Quantity: Changes (hours per day) spent on:**				
Housework	0.01	0.01	0.04*	0.04
	(0.02)	(0.02)	(0.02)	(0.03)
Employment	-0.00	-0.00	0.02	0.03
	(0.01)	(0.02)	(0.02)	(0.03)
Leisure	0.07***	0.08***	0.07***	0.08***
	(0.02)	(0.02)	(0.02)	(0.02)
Subsistence	0.04**	0.05**	0.03	0.03
	(0.02)	(0.02)	(0.02)	(0.03)
**B. Quality: Changes (hours per day) spent on:**				
Multitasking across categories	0.01*	0.01	0.01	0.01
	(0.01)	(0.01)	(0.01)	(0.02)
Leisure activity done alone	-0.03**	-0.04**	-0.04**	-0.04
	(0.01)	(0.02)	(0.02)	(0.02)
Unusual work hours	-0.02	-0.01	-0.08***	-0.07*
	(0.02)	(0.03)	(0.03)	(0.04)
Unusual housework hours	-0.01	-0.02	-0.04	-0.08
	(0.03)	(0.04)	(0.05)	(0.07)
**C. Sociodemographic covariates**				
WFH during LD1	0.14**	0.07		
	(0.06)	(0.06)		
WFH during LD3			0.06	0.11
			(0.07)	(0.08)
Female	-0.13**	-0.15**	0.07	0.16
	(0.05)	(0.06)	(0.08)	(0.11)
Has young child	0.01	0.01	0.04	0.13
	(0.08)	(0.10)	(0.12)	(0.14)
Female X Has young child	0.09	0.07	-0.19	-0.34*
	(0.11)	(0.13)	(0.14)	(0.18)
Has tertiary degree	-0.03	-0.02	0.14**	0.17*
	(0.05)	(0.06)	(0.07)	(0.10)
White	-0.10	-0.11	-0.11	-0.09
	(0.07)	(0.08)	(0.10)	(0.11)
Income: £1k-2k	-0.06	-0.04	-0.17*	-0.22**
	(0.06)	(0.07)	(0.09)	(0.11)
Income: £2k-3k	-0.15**	-0.11	-0.22**	-0.30**
	(0.07)	(0.08)	(0.11)	(0.14)
Income: £3k-4k	0.01	0.01	-0.03	0.00
	(0.07)	(0.10)	(0.13)	(0.16)
Income: £4k-5k	-0.00	0.10	0.04	0.04
	(0.10)	(0.12)	(0.16)	(0.21)
Income: £5k or above	-0.11	-0.11	0.22	0.18
	(0.16)	(0.20)	(0.16)	(0.21)
Baseline mean enjoyment	5.64	5.64	5.64	5.64
Baseline SD enjoyment	0.76	0.76	0.76	0.76
Baseline mean change in enjoyment	-0.01	-0.01	-0.28	-0.28
Baseline SD change in enjoyment	0.61	0.61	0.94	0.94
Observations	762	710	761	709

Table S46. Weighted and unweighted estimates of correlations between within-person changes in overall self-reported enjoyment and characteristics of time use. Sociodemographic covariates were used as controls. Reported changes during the first and third lockdown (May 2020 and March 2021, respectively) were relative to the pre-pandemic timepoint (February 2020). Robust standard errors are reported in brackets. A coefficient of 0.1 corresponds to ~0.13 SD in pre-pandemic enjoyment levels. *** $p < 0.01$, ** $p < 0.05$, * $p < 0.1$.

9 Other data quality issues

9.1 Selection on unobservables

We showed that our results are robust to accounting for attrition bias and re-weighting to match the composition of the UK workforce. However, our reweighted data may still suffer from selection in ways that correlate with outcomes of interest. For example, we conducted our survey online, so are more likely to reach individuals who spend more time online. We cannot rule out the possibility that these participants systematically differ from non-participants in their lockdown experiences and time use. Still, given the widespread use of broadband and smartphones in the UK, this issue is less of a concern than it would have been a decade ago. In fact, older respondents (aged 55 and above), who are conventionally seen as less likely to be online, are over-represented in our longitudinal sample.

9.2 Measurement error in time use diaries

There are two main sources of measurement error arising from our time use diary methodology: recall bias and individual-specific variation in recording activities. Any measurement error in time spent on various activities will create attenuation bias in our estimates, so the true size of the changes may be larger than those we document.

Firstly, the information for the pre-pandemic timepoint (February 2020) was obtained in May 2020, three months after the particular days of interest actually occurred, and so may be less accurate than the information for the other two timepoints, where respondents recalled events of one or two days ago. We tried to mitigate this issue by encouraging respondents to refer to their planners when completing the time use diaries.

To investigate potential differences in accuracy across timepoints, we compared the distribution of episode start and end minutes. Doing so enables detection of rounding (for example, to the nearest hour), which is more likely to occur if respondents could not remember the exact start or end time of an episode. Figure S47 shows that these distributions were very similar across all timepoints, suggesting that the degree of recall bias due to rounding is unlikely to vary across timepoints. Table S7 (Section 3) shows that across all timepoints, the proportions of episodes that need cleaning due to mis-recording were very small (less than 0.1%) and similar across timepoints.

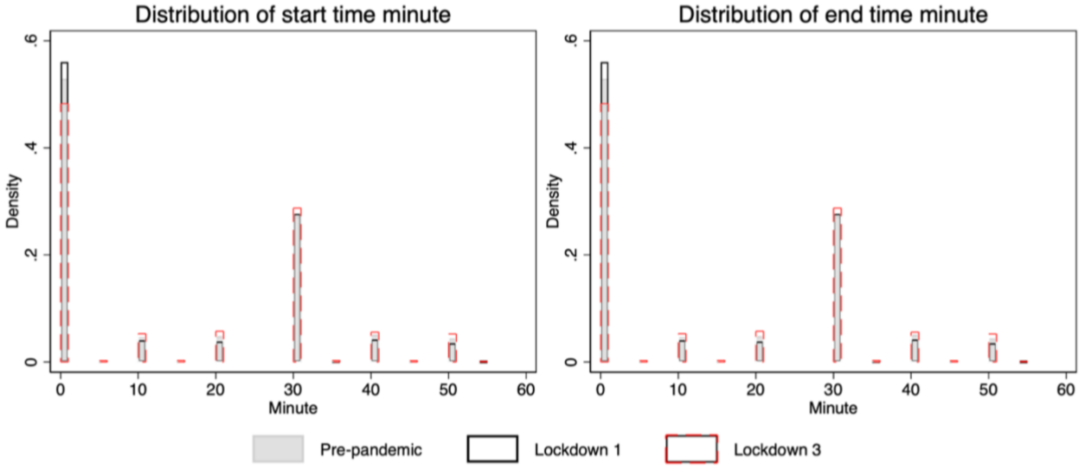

Figure S47. Histogram of the start and end minute of episodes, by timepoint. Time was measured in 10-minute intervals.

Second, since time use diaries were self-completed, there may be individual-specific variation in recording of activities. The self-completion method has been shown to obtain similar data quality compared to the external coding method used by 2014/15 UKTUS or objective real-time instruments

[8,9]. We used the following methods to improve the standardization of responses and ensure our main results are robust to such variation:

- Requiring respondents to complete a 5-10-minute tutorial on how to fill in time use diaries. We provided details instructions in PDF format which respondents could refer to when filling in their own diaries. Before filling in their own diaries, respondents had to correctly fill in three fictional time use diaries according to the instructions provided.
- Focusing on within-person differences, which will ‘difference out’ this variation (to the extent that such variation is constant across time for each individual).

Using broad activity categories. At the level of aggregation used in our main analysis, it is highly unlikely that respondents will mis-classify activities, for example, mistaking a subsistence activity for a leisure activity. There may be some differences in the way that respondents record main and secondary activities, but the supplementary analysis in Section 6.1 shows that we obtain similar results (in sign and statistical significance), albeit of a different magnitude, when considering time spent in the main and secondary activity or the secondary activity only.

References

1. Hale T, Angrist N, Goldszmidt R, Kira B, Petherick A, Phillips T, et al. A global panel database of pandemic policies (Oxford COVID-19 Government Response Tracker). Nat Hum Behav. 2021 Apr;5(4):529–38.

2. Palan S, Schitter C. Prolific.ac—A subject pool for online experiments. J Behav Exp Finance. 2018 Mar 1;17:22–7.

3. Peer E, Samat S, Brandimarte L, Acquisti A. Beyond the Turk: Alternative Platforms for Crowdsourcing Behavioral Research [Internet]. Rochester, NY: Social Science Research Network; 2016 May [cited 2021 May 22]. Report No.: ID 2594183. Available from: https://papers.ssrn.com/abstract=2594183

4. Hamermesh DS. Life satisfaction, loneliness and togetherness, with an application to Covid-19 lock-downs. Rev Econ Househ. 2020 Dec 1;18(4):983–1000.

5. Gershuny J, Sullivan O, Sevilla A, Vega-Rapun M, Foliano F, Grignon JL de, et al. A new perspective from time use research on the effects of social restrictions on COVID-19 behavioral infection risk. PLOS ONE. 2021 Feb 10;16(2):e0245551.

6. Gimenez-Nadal JI, Velilla J. Home-based work, time endowments, and subjective well-being: Gender differences in the United Kingdom [Internet]. MPRA Paper. University Library of Munich, Germany; 2020 [cited 2021 May 22]. (MPRA Paper). Report No.: 104937. Available from: https://ideas.repec.org/p/pra/mprapa/104937.html

7. Bond TN, Lang K. The Sad Truth about Happiness Scales. J Polit Econ [Internet]. 2019 Jun 10 [cited 2021 May 31]; Available from: https://www.journals.uchicago.edu/doi/abs/10.1086/701679

8. Gershuny J, Harms T, Doherty A, Thomas E, Milton K, Kelly P, et al. Testing Self-Report Time-Use Diaries against Objective Instruments in Real Time. Sociol Methodol. 2020 Aug 1;50(1):318–49.

9. Sullivan O, Gershuny J, Sevilla A, Walthery P, Vega-Rapun M. Time use diary design for our times - an overview, presenting a Click-and-Drag Diary Instrument (CaDDI) for online application. J Time Use Res. 2020 Jul 16;1–17.
