# Supplementary material for: Changes in the quantity and quality of time use during the COVID-19 lockdowns in the UK: Who is the most affected?
Source: PLoS One. 2021 Nov 3;16(11):e0258917. doi: 10.1371/journal.pone.0258917 (PMC8565783; doi:10.1371/journal.pone.0258917)
Supplement: S2 Appendix — (PDF) [file pone.0258917.s002.pdf]

S2 Appendix for

Changes in the quantity and quality of time-use during the COVID-19 lockdowns in the UK: Who is the most affected?

Ines Lee Eileen Tipoe

This document contains a copy of the survey used to collect data for this analysis. Text in red was not shown to participants. Data collection methods were approved by the Institutional Review Board at the University of Oxford (approval code ECONCIA20-21-16).

1. General Information about this Study

We are inviting you to participate in this study about how the first COVID-19 induced national lockdown (beginning in March 2020) affected your time-use patterns.

(Note: In the Wave 2 survey, this sentence was replaced with 'We are inviting you to participate in this study because you took part in our earlier survey about how the third COVID-19 induced national lockdown (beginning in 5 January 2021) affected your time-use patterns.')

You will be asked to describe the sequence and duration of activities you engage in during a typical working and non-working day during this lockdown period. You will also be asked some questions about yourself and your current work arrangements.

The entire study should take you about 30 minutes. **Please do not worry if the study takes longer than expected or if you receive a 'Time out' notice from Prolific; we will compensate you via a bonus if you need significantly more than 30 minutes to complete this questionnaire.**

Before starting, please read through the information in the consent form below (you can download and keep a copy for your reference).

Read and download consent form here: Consent form (opens in a new window)

Please note that your participation in this study is entirely voluntary. If you do decide to take part, you can still withdraw at any point during the study for any reason by closing the browser. If you choose to withdraw, your responses will not be recorded or used for the study. You must complete all of the applicable time-use diaries in order to be paid for your participation.

To agree to participate, please verify all the statements below.

You may only participate in this survey if you are **18 years of age or over**.

☐ I certify that I am 18 years of age or over. (1)

You may only participate in this survey if you have **lived in the UK for the past 3 months**.

☐ I certify that I have lived in the UK for the past 3 months. (1)

If you have read the information above and in the document, and agree to participate with the understanding that the data (including any personal data) you submit will be processed accordingly, please check the relevant box below to get started.

☐ Yes, I agree to take part. (1)

Please enter your Prolific ID here.
(This should be filled in automatically. If it is not, please copy and paste your Prolific ID into the space below.)

____

2. Demographic questions

2.1 Which gender best describes you?

- ○ Male
- ○ Female
- ○ Other

2.2 In which year were you born?

[Drop-down menu with years ranging from 1934 to 2002]

2.3 Which ethnic group/groups do you identify with? (Select all that apply.)

- ○ White British
- ○ All other White
- ○ Asian/Asian British
- ○ Black/African/Caribbean/Black British
- ○ Other ethnic group (please specify): ________________________

2.4 What is your highest educational attainment level?
(If you did not study in the UK, choose the option that you think is the closest equivalent.)

- ○ Pre-GCSE/O-level or equivalent
- ○ GCSEs/O-levels
- ○ A-levels or equivalent
- ○ Vocational degree
- ○ Undergraduate degree
- ○ Graduate degree (e.g. MPhil, MSc, PhD)

2.5 Which region of the UK do you live in?

- North East
- North West
- Yorkshire and the Humber
- East Midlands
- West Midlands
- East England
- London
- South East
- South West
- Wales
- Scotland

2.6 Do you live alone?

- Yes (go to Section 3)
- No (go to Q2.7)

2.7 Please fill in the following information for all members of your household, excluding yourself.

(If there are more than 10 people in your household, excluding yourself, please list the 10 people who are the most dependent on your income.)

	**Relationship to you**	**Age**
1	▼ Spouse/Partner, Child, Parent, Sibling, Grandchild, Grandparent, Other relative, Other non-relative	▼ 5 and below, 6-11, 12-17, 18-59, 60 and above
2	▼ Spouse/Partner, Child, Parent, Sibling, Grandchild, Grandparent, Other relative, Other non-relative	▼ 5 and below, 6-11, 12-17, 18-59, 60 and above
3	▼ Spouse/Partner, Child, Parent, Sibling, Grandchild, Grandparent, Other relative, Other non-relative	▼ 5 and below, 6-11, 12-17, 18-59, 60 and above
4	▼ Spouse/Partner, Child, Parent, Sibling, Grandchild, Grandparent, Other relative, Other non-relative	▼ 5 and below, 6-11, 12-17, 18-59, 60 and above
5	▼ Spouse/Partner, Child, Parent, Sibling, Grandchild, Grandparent, Other relative, Other non-relative	▼ 5 and below, 6-11, 12-17, 18-59, 60 and above
6	▼ Spouse/Partner, Child, Parent, Sibling, Grandchild, Grandparent, Other relative, Other non-relative	▼ 5 and below, 6-11, 12-17, 18-59, 60 and above
7	▼ Spouse/Partner, Child, Parent, Sibling, Grandchild, Grandparent, Other relative, Other non-relative	▼ 5 and below, 6-11, 12-17, 18-59, 60 and above
8	▼ Spouse/Partner, Child, Parent, Sibling, Grandchild, Grandparent, Other relative, Other non-relative	▼ 5 and below, 6-11, 12-17, 18-59, 60 and above
9	▼ Spouse/Partner, Child, Parent, Sibling, Grandchild, Grandparent, Other relative, Other non-relative	▼ 5 and below, 6-11, 12-17, 18-59, 60 and above
10	▼ Spouse/Partner, Child, Parent, Sibling, Grandchild, Grandparent, Other relative, Other non-relative	▼ 5 and below, 6-11, 12-17, 18-59, 60 and above

3. Time use diary instructions and example

In this section, you will be asked to fill in **up to four time-use diaries**. Please note that you need to complete all applicable time-use diaries to be paid for your participation.

Please download and read the following document, which contains instructions on how to fill in the time-use diaries: Time-use diary instructions.pdf (opens in a new window)

On the following page, there are a few examples of fictitious time-use diaries which you can use to check your understanding before filling in your own diaries.

In the document you downloaded, we saw some examples of how Rob would fill in the entries for a typical morning. Here is how Rob would fill in three entries for a typical afternoon.

Example 1.
Time: 1.00pm - 3.00pm
Activity: Take over homeschooling from Sarah (go through Geometry and Algebra with kids). Spend 30 undistracted minutes proofreading report from work colleague on iPad when kids are doing homework.

Enjoyment: 4/7

This is how Rob should fill in this entry.

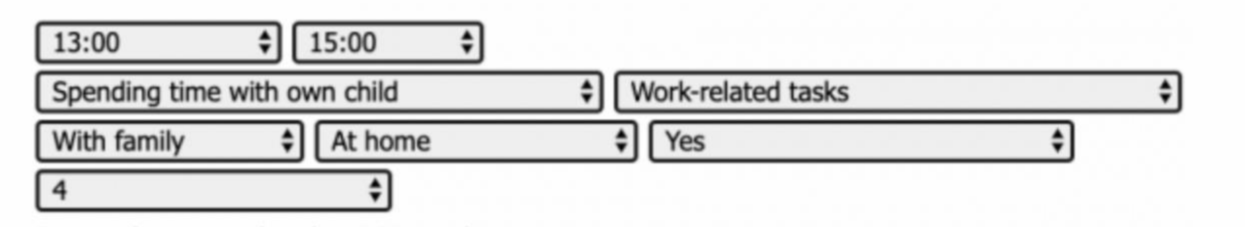

Example 2.
Time: 6.00pm - 6.30pm
Activity: Go for a run around the park. Listen to music on iPhone while running.
Enjoyment: 6/7

This is how Rob should fill in this entry.

- Since Rob was listening to music on his phone while running, we've selected the secondary activity to match this.

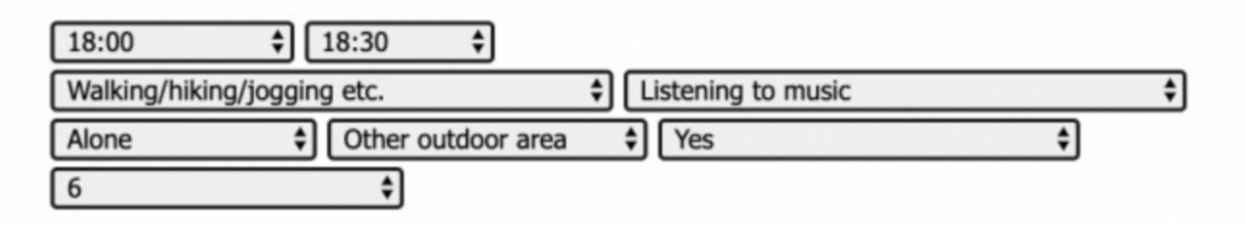

Example 3.
Time: 6.30pm - 7.00pm
Activity: Prepare dinner with Sarah. Listen to news podcast together on her phone.
Enjoyment: 7/7

This is how Rob should fill in this entry.

- Since Sarah and Rob listened to a podcast while making dinner and since there is no specific "podcast" option, we've selected "other mass media activity" for the secondary activity.

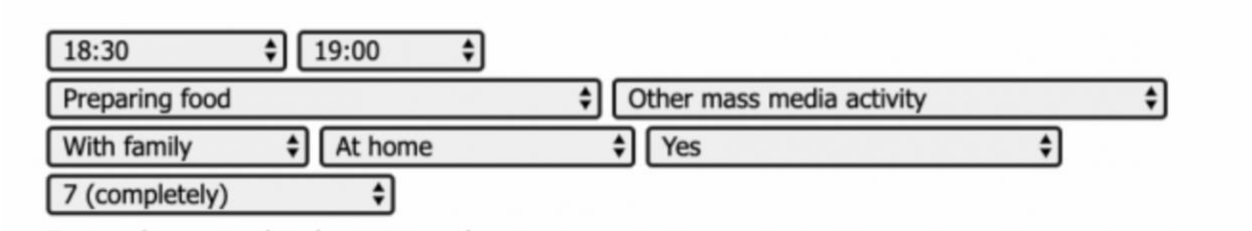

Click the 'next' arrow to fill in your own time-use diaries on the following pages.

4. My Time-use diary for a Working Day before the First Lockdown

Which of the following options best describes your situation?
(For reference: The first national lockdown was implemented on 23 March 2020).

◯ Before the first national lockdown was implemented, I have done paid work for at least one day.

◯ Before the first national lockdown was implemented, I have not done any paid work. (Go to Section 5).

(Note: In the Wave 2 survey, 'first national lockdown' was replaced with 'third national lockdown' and the reference date was changed to 6 January 2021.)

Think back to the last **working day** you had **before this lockdown**. Please fill in the following time-use entries to reflect this working day.

In order to be paid for your participation in this survey, you must complete all time-use diaries that are applicable to you. "Complete" means that your diary starts from midnight (12.01am; start time of "00:00") on that day and ends at 11.59pm (start time of "0 (next day)"). On average, people do around 10 to 20 activities per day.

It can be difficult to recall what you did that day, so we encourage you to refer back to any records you have (e.g. on your digital or physical calendar). When you are done with your entries for the day, please click the "I've finished" statement at the bottom of the page. Otherwise, you may add additional entries on the following pages.

Remember that instead of scrolling through the drop down menus, you can type a number or letter to be directed to your desired option. E.g. You can type "18" in the start time box to be directed to 18.00 hours.

Note: Each entry looks like the one below. The options for each drop-down menu are as follows:

- **Start time, End time:** 0:00 to 23:50, in 10-minute intervals
- **Main activity, Secondary activity:** Cooking, Groceries, Cleaning, Childcare, Caring for pets, Other caring duties, Bills/household accounts, Gardening, Repairs, Other household activities, Work tasks, Meetings, Searching for jobs, Casual work, Tea/Coffee break, Other employment-related activities, Communicating with others, Watching movie at the cinema, Attending a social gathering, Shopping (non-groceries), Having visitors/visiting a friend, Going to a museum/other cultural site, Other social/cultural activities, Playing games (any type), Doing crafts, Doing art, Singing, Other arts/hobbies, Watching TV, Social media, Listening to music, Reading/watching/listening to the news, Reading, Other mass media consumption, Walking, Other exercise, Volunteering, Sleeping, Eating, Personal care
- **Who with:** Alone, With family, With friends, With family and friends, Other
- **Where:** At home, At workplace, On a mode of transport, Other indoor area, Other outdoor area
- **Used smartphone/tablet/laptop:** Yes, No
- **Enjoyment for this interval:** 1 (not at all), 2, 3, 4, 5, 6, 7 (completely)

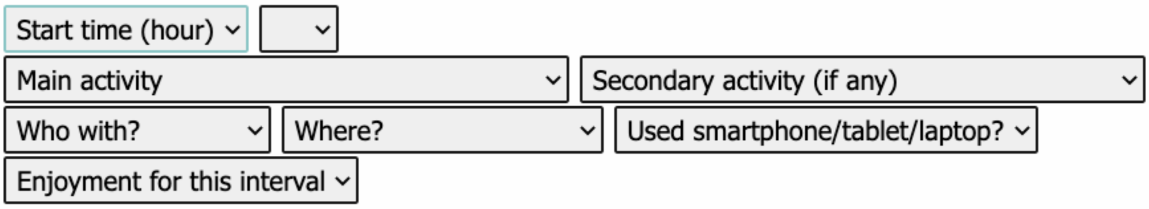

5. My Time-Use Diary for a Non-Working Day before the First Lockdown

Think back to the last **non-working day** you had **before this lockdown**. Please fill in the following time-use entries to reflect this non-working day.

In order to be paid for your participation in this survey, you must complete all time-use diaries that are applicable to you. "Complete" means that your diary starts from midnight (12.01am; start time of "00:00") on that day and ends at 11.59pm (start time of "0 (next day)"). On average, people do around 10 to 20 activities per day.

It can be difficult to recall what you did that day, so we encourage you to refer back to any records you have (e.g. on your digital or physical calendar). When you are done with your entries for the day, please click the "I've finished" statement at the bottom of the page. Otherwise, you may add additional entries on the following pages.

Remember that instead of scrolling through the drop down menus, you can type a number or letter to be directed to your desired option. E.g. You can type "18" in the start time box to be directed to 18.00 hours.

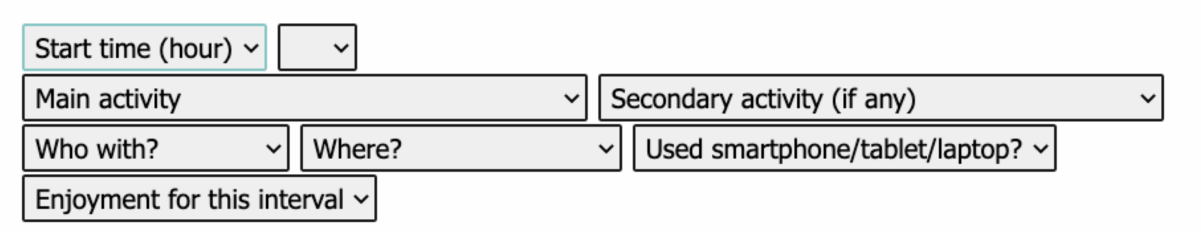

6. My Time-Use Diary for a Working Day during the First Lockdown

Which of the following options best describes your situation?
(For reference: The first national lockdown was implemented on 23 March 2020).

- ○ Since the first national lockdown was implemented, I have done paid work for at least one day.

- ○ Since the first national lockdown was implemented, I have not done any paid work. (Go to Section 7).

(Note: In the Wave 2 survey, 'first national lockdown' was replaced with 'third national lockdown' and the reference date was changed to 6 January 2021.)

Think back to the last **working day** you had **during this lockdown**. Please fill in the following time-use entries to reflect this working day.

In order to be paid for your participation in this survey, you must complete all time-use diaries that are applicable to you. "Complete" means that your diary starts from midnight (12.01am; start time of "00:00") on that day and ends at 11.59pm (start time of "0 (next day)"). On average, people do around 10 to 20 activities per day.

It can be difficult to recall what you did that day, so we encourage you to refer back to any records you have (e.g. on your digital or physical calendar). When you are done with your entries for the day, please click the "I've finished" statement at the bottom of the page. Otherwise, you may add additional entries on the following pages.

Remember that instead of scrolling through the drop down menus, you can type a number or letter to be directed to your desired option. E.g. You can type "18" in the start time box to be directed to 18.00 hours.

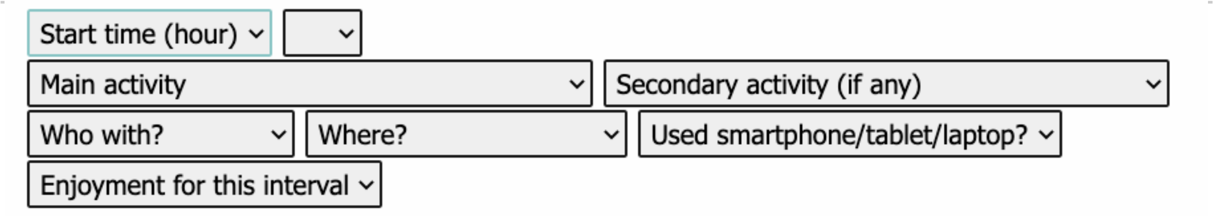

7. My Time-Use Diary for a Non-Working Day during the First Lockdown

Think back to the last **non-working day** you had **during this lockdown**. Please fill in the following time-use entries to reflect this non-working day.

In order to be paid for your participation in this survey, you must complete all time-use diaries that are applicable to you. "Complete" means that your diary starts from midnight (12.01am; start time of "00:00") on that day and ends at 11.59pm (start time of "0 (next day)"). On average, people do around 10 to 20 activities per day.

It can be difficult to recall what you did that day, so we encourage you to refer back to any records you have (e.g. on your digital or physical calendar). When you are done with your entries for the day, please click the "I've finished" statement at the bottom of the page. Otherwise, you may add additional entries on the following pages.

Remember that instead of scrolling through the drop down menus, you can type a number or letter to be directed to your desired option. E.g. You can type "18" in the start time box to be directed to 18.00 hours.

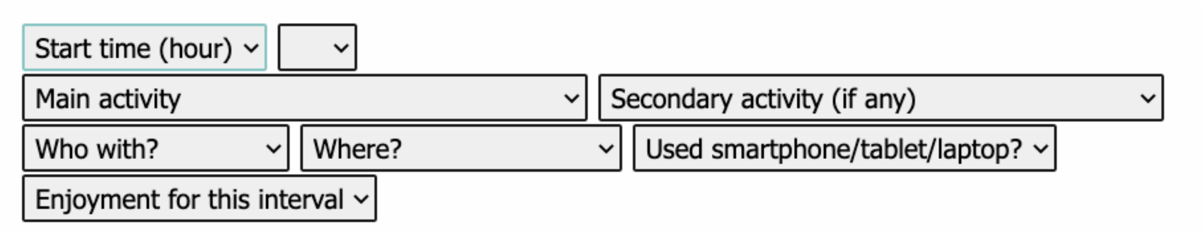

8. Questions about your employment situation

8.1 (Only shown to respondents who reported doing at least one day of paid work before the first lockdown) Please select the option that best applies to your working arrangements before the first national lockdown.

- ◯ Worked entirely from home
- ◯ Mix of home and on-site work
- ◯ Worked entirely on-site

8.2 (Only shown to respondents who reported doing at least one day of paid work during the first lockdown) Please select the option that best applies to your working arrangements during the first national lockdown.

- ◯ Worked entirely from home
- ◯ Mix of home and on-site work
- ◯ Worked entirely on-site

(Note: For the Wave 2 survey, Q8.1 and 8.2 were replaced with a single question, asking 'Please select the option that best applies to your working arrangements during the third national lockdown', containing the same options).

Think about your main job (if you have more than one job, your main job provides the majority of your monthly income).

8.3 Which of the following best describes the type of contract that you have for this job?

- ◯ Self-employed (1)
- ◯ Fixed monthly salary (with or without bonus) (2)
- ◯ Hourly wage (3)
- ◯ Zero hours contract (no minimum working hours) (4)

8.4 Which of the following occupational categories best describes your role in this job?
Please select one option.

[Drop-down menu options: Corporate manager/director, Other manager/proprietor, Science/Research/Engineering/Technology professional, Science/Research/Engineering/Technology associate professional, Health professional, Health and social care associate professional, Teaching/Educational professional, Business/Media/Public service professional, Business/Media/Public service associate professional, Protective service occupations, Culture/Media/Sports occupations, Administrative occupations, Secretarial and related occupations, Agricultural and related trades, Metal/Electrical/Electronic trades, Construction/Building trades, Textiles/Printing trades, Caring/Caretaking occupations, Leisure/Travel occupations, Sales occupations, Customer service occupations, Process/Plant/Machine operatives,

Transport/Mobile machine drivers and operatives, Elementary trades and related occupations, Elementary administration and service occupations]

8.5 What is your monthly before-tax income from this job?
Please select one option.

[Drop-down menu options: 0 GBP, 1-500 GBP, 501-1000 GBP, 1001-1500 GBP, 1501-2000 GBP, 2001-2500 GBP, 2501-3000 GBP, 3001-3500 GBP, 3501-4000 GBP, 4001-4500 GBP, 4501-5000 GBP, More than 5000 GBP]
8.6 In the last 7 days, how many hours did you spend working for this job?
Please select one option.

[Drop-down menu options: 0 hours, 1-5 hours, 6-10 hours, 11-20 hours, 21-30 hours, 31-40 hours, 41-50 hours, 51 hours or more]

You have reached the end of the survey. Thank you for taking the time to complete it!
Click the 'Next' button to return to Prolific.
