# Supplementary material for: Changes in the quantity and quality of time use during the COVID-19 lockdowns in the UK: Who is the most affected?
Source: PLoS One. 2021 Nov 3;16(11):e0258917. doi: 10.1371/journal.pone.0258917 (PMC8565783; doi:10.1371/journal.pone.0258917)
Supplement: S1 File — (ZIP) [file pone.0258917.s003.zip › Replication/_ReadMe.docx]

**Changes in the quantity and quality of time use during the COVID-19 lockdowns in the UK: Who is the most affected?**

*Replication files*

This document guides researchers to replicate the figures and corresponding tables in *“Changes in the quantity and quality of time use during the COVID-19 lockdowns in the UK: Who is the most affected?”* by Ines Lee ([il300@cam.ac.uk](mailto:il300@cam.ac.uk)) and Eileen Tipoe ([e.tipoe@qmul.ac.uk](mailto:e.tipoe@qmul.ac.uk)).

# Data Files

The *Data* folder consists of .dta files required to generate the figures and corresponding tables in our main analysis:

- panel_wide_rep.dta is the main dataset in wide format
- panel_long_rep.dta is the main dataset in long format

panel_wide_rep.dta contains more variables than panel_long_rep.dta since the former is used to run regressions with additional individual-level controls.

# Replication Files

**Running the replication code**

The statistical analysis in this paper uses Stata version 16.1. The *Replication_main.do* file generates the main figures (and corresponding tables) found in the main paper.

To run the replication .do file, the user first needs to change the root path to be the path where the replication folder is stored (e.g. “your-path/Replication”). All other directories used in the .do files are defined relative to the root path.

**Installing ADO files**

Some of our code use community-provided commands that may not be installed by default in the user’s Stata software.

Examples of ado files that the code uses but may not be installed on the user’s Stata software include:

- The **coefplot** command by Ben Jann (<http://repec.sowi.unibe.ch/stata/coefplot/index.html>)
- The **esttab** command by Ben Jann (<http://repec.sowi.unibe.ch/stata/estout/>)
- The **grc1leg** command
- The **putexcel** command (<https://www.stata.com/manuals/rptputexcel.pdf>)

If these commands are not recognized by your Stata software, please download the relevant ado files. This can be done by typing -findit <command name>- or -ssc install <command name>- into the Stata command line.

If you already have the relevant commands installed but still have problems running the code, you may wish to update the command. You can do this by tying -ssc install <command name>, replace-.
