# Supplementary figures and images for: Changes in the quantity and quality of time use during the COVID-19 lockdowns in the UK: Who is the most affected?
Source: PLoS One. 2021 Nov 3;16(11):e0258917. doi: 10.1371/journal.pone.0258917 (PMC8565783; doi:10.1371/journal.pone.0258917)

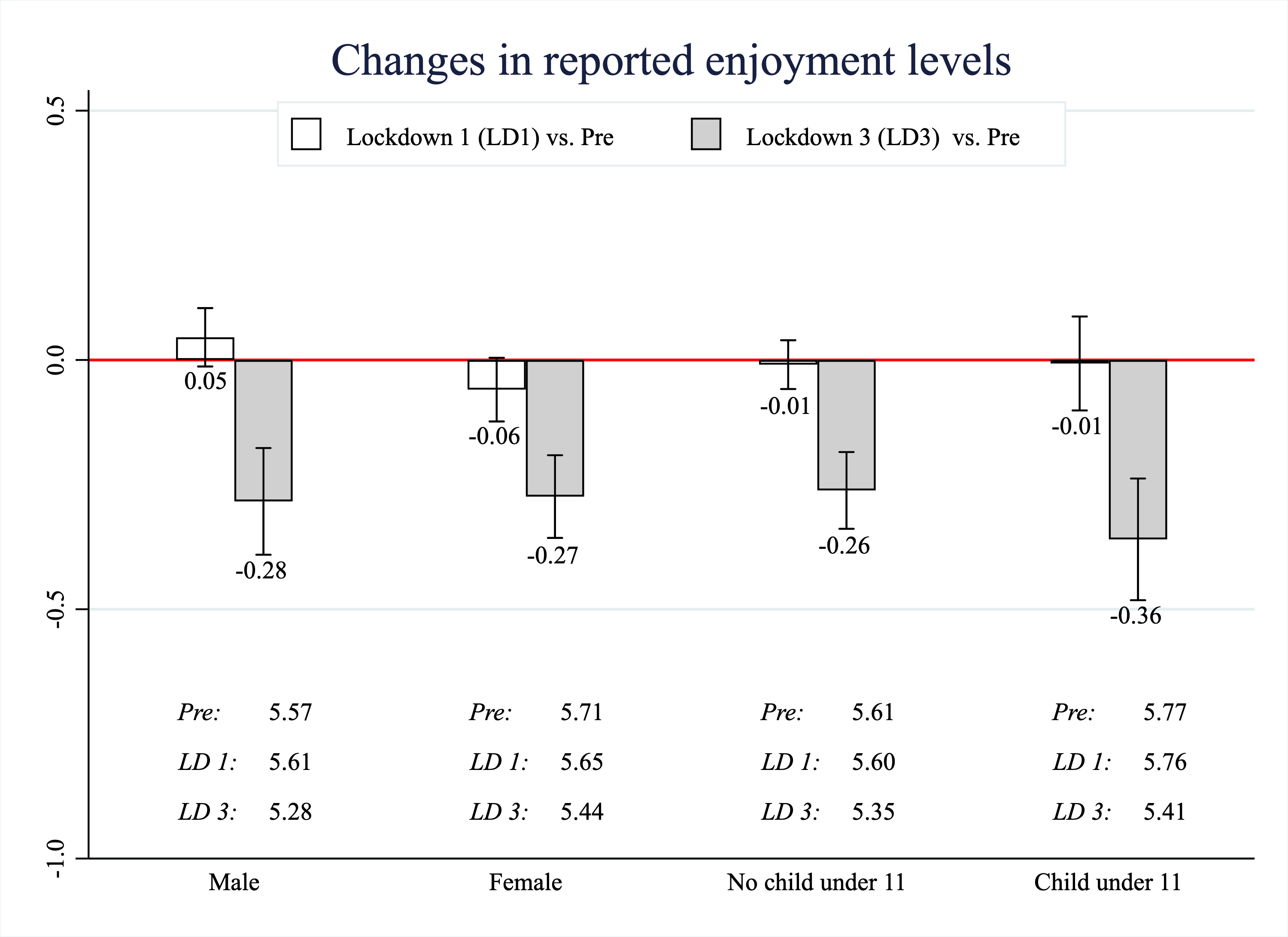

Supplement: S1 File — (ZIP) [file pone.0258917.s003.zip › Replication/Output/Figure4.tif]

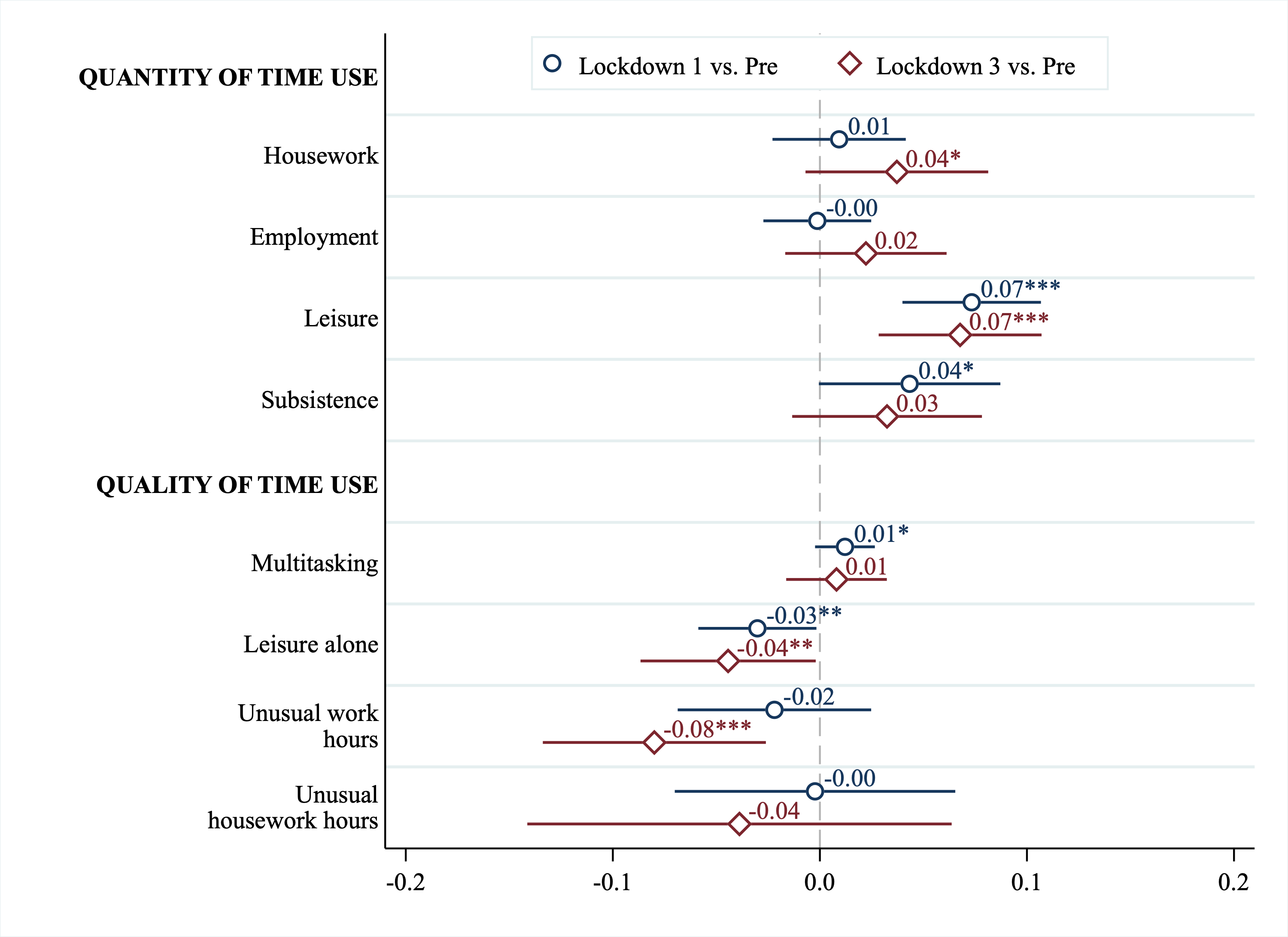

Supplement: S1 File — (ZIP) [file pone.0258917.s003.zip › Replication/Output/Figure5.tif]

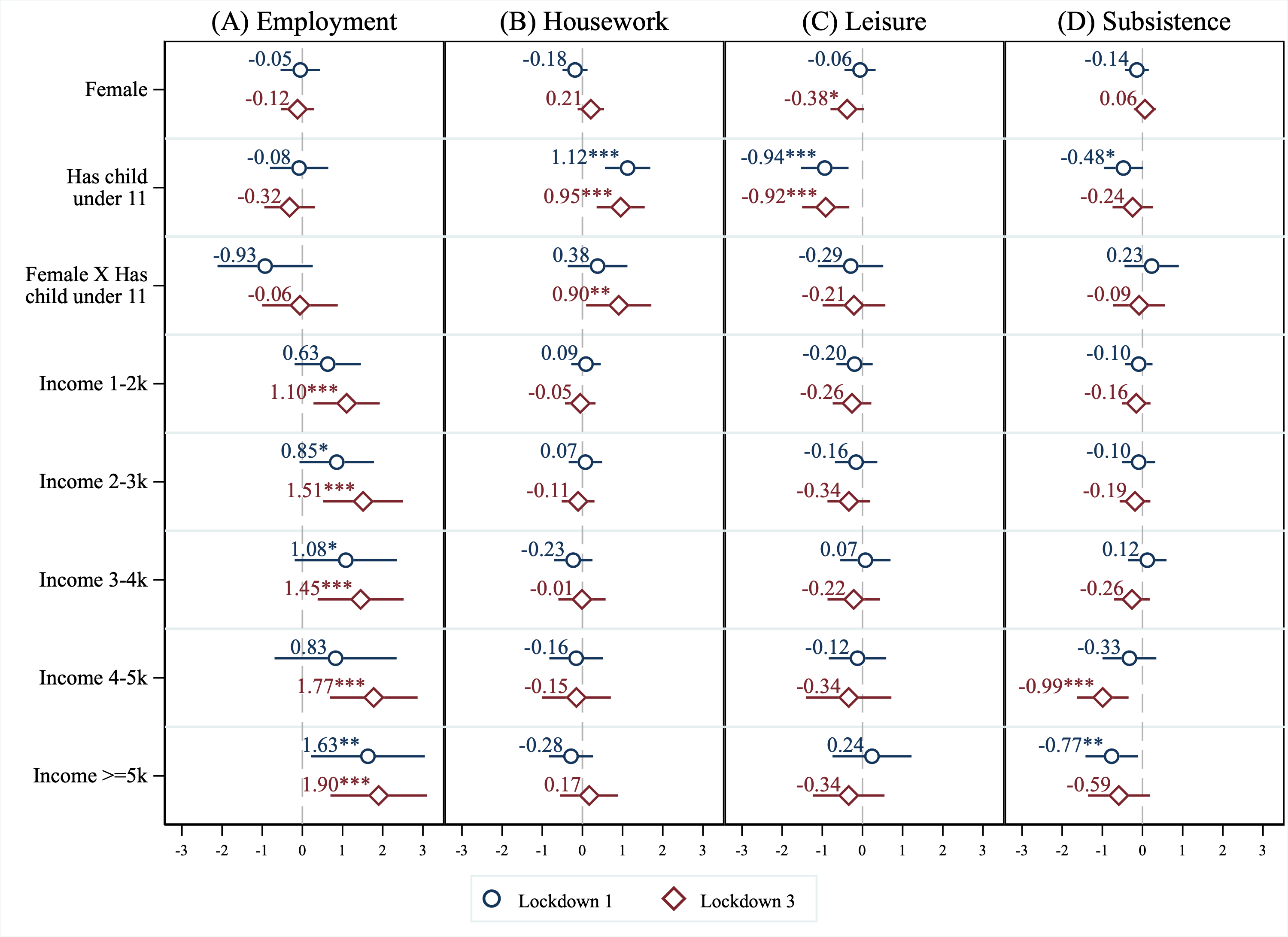

Supplement: S1 File — (ZIP) [file pone.0258917.s003.zip › Replication/Output/Figure2.tif]

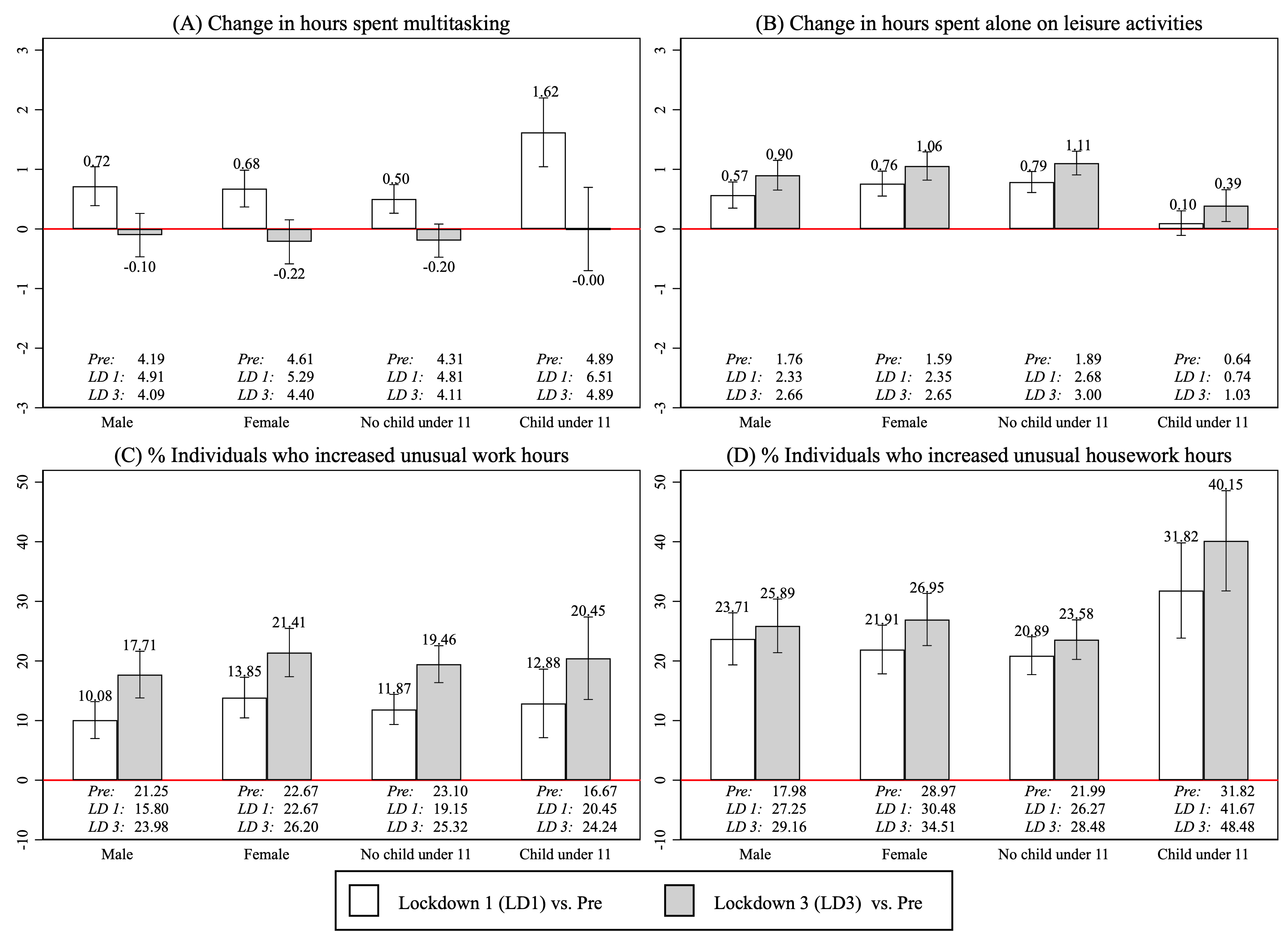

Supplement: S1 File — (ZIP) [file pone.0258917.s003.zip › Replication/Output/Figure3.tif]

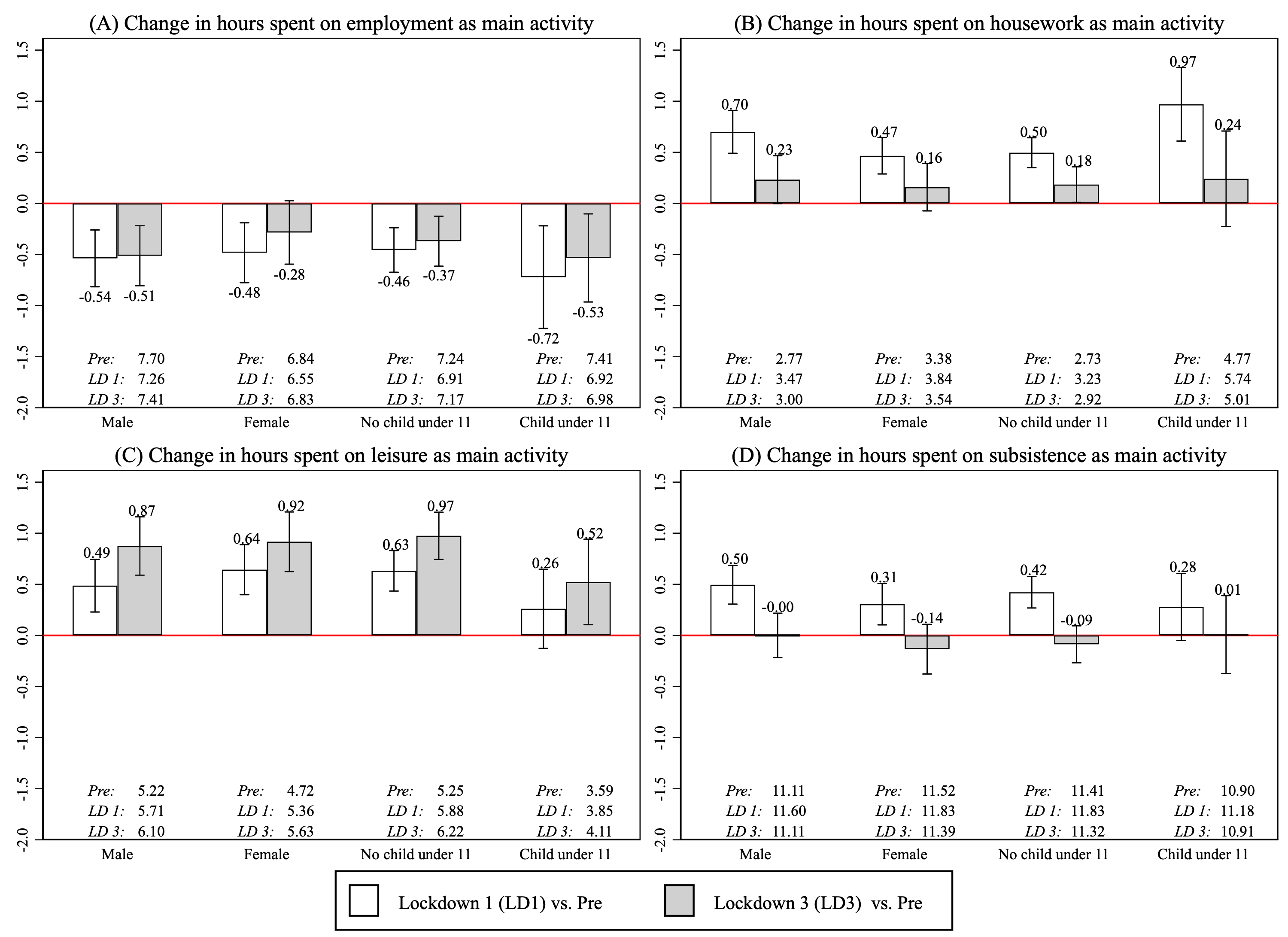

Supplement: S1 File — (ZIP) [file pone.0258917.s003.zip › Replication/Output/Figure1.tif]
